# A muscle-enriched mitochondrial protein regulates postnatal heart growth through mitonuclear ubiquinone–PPAR signaling

**DOI:** 10.1073/pnas.2619615123

**Published:** 2026-10-16

**Authors:** Kentaro Hirose, Xiaoxin Chen, Chakrapani Kalyanaraman, Mark T. Waddingham, Makoto Nakamura, Feng Zhang, Christina Chen, Shun Yao, Steven Chang, Hongyao Yu, Jiajia Wang, Emanuele Barca, Haruko Nakano, Austin Hsu, Sheamin Khyeam, Sara Gonzalez Ortega, Kazuki Tajima, Takeshi Yoneshiro, Hao Zhang, Arun Padmanabhan, Shingo Kajimura, Saptarsi Haldar, Atsushi Nakano, Masatoshi Murai, Yoshitaka Fujihara, Kinya Otsu, Catarina M. Quinzii, Matthew P. Jacobson, Guang Hu, James T. Pearson, Guo N. Huang

**Affiliations:** ^a^ Cardiovascular Research Institute, University of California, San Francisco, CA 94158; ^b^ Department of Physiology, University of California, San Francisco, CA 94158; ^c^ Eli and Edythe Broad Center for Regeneration Medicine and Stem Cell Research, University of California, San Francisco, CA 94158; ^d^ Bakar Aging Research Institute, University of California, San Francisco, CA 94158; ^e^ Department of Cardiac Physiology, National Cerebral and Cardiovascular Center Research Institute, Suita 564-8565, Osaka, Japan; ^f^ Cambridge Stem Cell Institute, University of Cambridge, Cambridge CB2 0AW, United Kingdom; ^g^ Department of Pharmaceutical Chemistry, University of California, San Francisco, CA 94158; ^h^Nine Square Therapeutics, Inc., South San Francisco, CA 94080; ^i^ Epigenetics and Stem Cell Biology Laboratory, National Institute of Environmental Health Sciences, Research Triangle Park, NC 27709; ^j^ Department of Neurology, Columbia University Medical Center, New York, NY 10032; ^k^ Department of Molecular, Cell, and Developmental Biology, University of California, Los Angeles, CA 90095; ^l^ Gladstone Institutes, San Francisco, CA 94158; ^m^ University of California, San Francisco Diabetes Center, San Francisco, CA 94143; ^n^ Department of Medicine, Division of Cardiology, University of California, San Francisco, CA 94158; ^o^ Department of Medicine, Beth Israel Deaconess Medical Center, Boston, MA 02215; ^p^ Division of Applied Life Sciences, Graduate School of Agriculture, Kyoto University, Kyoto 606-8502, Japan; ^q^ Department of Advanced Medical Technologies, National Cerebral and Cardiovascular Center, Suita, Osaka 564-8565, Japan; ^r^ Office of the President, National Cerebral and Cardiovascular Center, Suita, Osaka 564-8565, Japan

**Keywords:** cardiac hypertrophy, mitochondria, Coq10a, ubiquinone, PPAR signaling

## Abstract

Increased mitochondrial biogenesis and aerobic respiration in postnatal mammalian organs are essential for adaptation to extrauterine life, yet the functions of many tissue-enriched, developmentally regulated mitochondrial proteins remain poorly defined. By integrating mitochondrial proteome annotations with mouse and human transcriptomic datasets, we identified Coenzyme Q10a (Coq10a), a striated muscle-enriched mitochondrial protein, as a regulator of postnatal cardiac growth. Coq10a, the mammalian homolog of the yeast ubiquinone-binding protein Coq10p, is highly induced in the heart after birth through thyroid hormone-, Nkx2.5-, and Tbx5-dependent regulation. To define its physiological function, we generated three independent *Coq10a*-deficient mouse models. Both global and cardiomyocyte-specific loss of *Coq10a* promoted postnatal cardiac hypertrophy without impairing basal cardiac function or mitochondrial respiration. Instead, *Coq10a* deficiency altered intracellular ubiquinone partitioning, resulting in increased ubiquinone accumulation in a microsomal membrane fraction. Transcriptomic analyses revealed enrichment of PPAR-associated metabolic gene sets and induction of established PPAR-responsive genes in *Coq10a*-deficient hearts. Pharmacological inhibition of PPARα and PPARγ, but not PPARδ, attenuated the hypertrophic phenotype. Molecular dynamics simulations, docking analyses, and mutagenesis identified PPARα residues that contribute to differential responsiveness to ubiquinone. Although *Coq10a*-deficient hearts maintained near-normal function under basal conditions, they exhibited increased susceptibility to pressure-overload stress. Together, these findings establish Coq10a as a regulator of intracellular ubiquinone partitioning and support a model linking altered mitochondrial lipid distribution and PPAR-associated signaling to postnatal cardiac growth. More broadly, they demonstrate how tissue-enriched mitochondrial proteins can mediate organ-specific physiological functions beyond energy production.

## Significance

Although mitochondria are recognized for their role in energy production, many mitochondrial proteins show tissue-specific expression patterns and their physiological functions remain unknown. Using a systematic analysis of tissue-enriched mitochondrial proteins, we identify Coenzyme Q10a (Coq10a) as a regulator of postnatal cardiac growth. *Coq10a* deficiency promotes postnatal cardiac hypertrophic growth, alters intracellular ubiquinone distribution without impairing mitochondrial respiration, and increases PPAR-responsive gene expression; pharmacological inhibition of PPARα/γ signaling attenuates this phenotype. Computational modeling and mutagenesis identify PPARα residues that contribute to differential responsiveness to ubiquinone. These findings support a model in which altered intracellular ubiquinone partitioning influences developmental heart growth through PPAR-associated signaling and demonstrate how tissue-specific mitochondrial proteins can regulate organ physiology through signaling functions independent of energy production.

Traditionally, mitochondria were primarily viewed as the powerhouse of the cell. However, it has been clear that mitochondria also function as intracellular signaling platforms because mitochondrial metabolites, lipids, and proteins play essential roles in regulating cellular responses and organ physiology (1). Although most mitochondrial components in energy production are ubiquitously expressed, many mitochondrial proteins exhibit tissue-specific expression and may have unique functions in particular organs (2). Here we systematically analyzed mouse and human organ-specific mitochondrial proteins with profound elevation of expression after birth during mitochondrial biogenesis and tissue maturation.

This analysis identified a previously unrecognized component in the ubiquinone (UQ, also known as coenzyme Q) pathway and its unexpected role in vivo. UQ was initially isolated from mitochondria in beef heart (3), and the heart contains one of the highest levels of UQ (4). As the evolutionarily conserved electron carrier in all eukaryotes, the lipid UQ plays a pivotal role in the electron transport chain by carrying electrons from mitochondrial respiratory chain complexes I and II to complex III (5–7). The synthesis and transport of UQ require orchestrated actions of a complex of enzymes and ancillary proteins (6, 7). Recent studies also suggest that UQs can modulate protein function through direct binding to specific target proteins including PPARα and PPARγ (8). In yeast, the genes encoding UQ-biosynthase proteins (*COQ1*-*11*) have been identified from mutants with reduced UQ levels and defective mitochondrial respiration, and some of these mutant phenotypes can be rescued by supplementation of UQ (5). Among these Coq proteins, Coq1p-Coq9p proteins are known to be involved in UQ synthesis (5). The enzymatic functions of these Coq proteins are preserved in mammals (2).

The yeast COQ10 gene encodes Coq10p, a START (StAR-related lipid-transfer)-domain protein involved in UQ handling. Like other yeast coq family mutants, *coq10Δ* mutants have deficient mitochondrial respiration (9). However, unlike UQ biosynthesis mutants that lack UQ, yeast coq10Δ mutants retain nearly normal UQ levels at stationary phase (9, 10). Biochemical studies have shown that yeast Coq10p binds UQ (9, 11). This led to the hypothesis that Coq10p helps position or retain UQ at its proper location within mitochondria. Mammalian COQ10A is a functional co-ortholog of yeast Coq10p and contains a conserved START domain that is predicted to mediate UQ binding and can rescue mitochondrial respiration defects in yeast *coq10Δ* (9, 12, 13). However, its physiological role in mammalian tissues in vivo has not been established.

Patients or rodent models with mutations in various mitochondrial genes including Coq biosynthesis genes often develop cardiomyopathy (14). It has been reported that human patients carrying mutations in UQ biosynthesis genes (*PDSS1*, *PDSS2, COQ2*, *COQ4*, *COQ7*, *COQ8B*, *COQ9*) can develop cardiac defects including hypertrophic cardiomyopathy, dilated cardiomyopathy, and UQ deficiency (6, 15). Analyses of 5,000 knockout mouse lines by the International Mouse Phenotype Consortium revealed that knockouts of the mouse homolog of *COQ2*, *COQ4*, *COQ6*, *COQ8B*, *PDSS1,* or *PDSS2* in mice are homozygous lethal (16), highlighting the essential role of UQ synthesis in development. A mouse model *Coq9^R239X^* harboring a premature stop codon found in a human patient presents widespread tissue deficiency of UQ and develops signs of cardiac fibrosis (17). *COQ10A* has not been established as a human Mendelian disease gene.

Here we show that rodent Coenzyme Q10a (Coq10a), a homolog of human COQ10A, is predominantly expressed in cardiac mitochondria. The mRNA level of *Coq10a* is positively regulated by thyroid hormone signaling, Nkx2.5, and Tbx5 transcription factors in postnatal mouse hearts and isolated cardiomyocytes. To understand the physiological function of *Coq10a*, we generated three independent *Coq10a* knockout mouse models using the CRISPR/Cas9-based genome editing technique. *Coq10a* mutant mice developed cardiac hypertrophy during the first 2 wk of postnatal life, while maintaining preserved cardiac function and intact mitochondrial respiration in adulthood under basal physiological conditions. Loss of Coq10a also increased UQ abundance in the microsomal fraction, suggesting that Coq10a regulates intracellular UQ distribution. Given that PPAR family members are lipid-sensing transcription factors that play central roles in cardiac metabolic regulation (18), and that UQ has been reported to bind to and activate PPARα and PPARγ (8), we asked whether altered UQ partitioning might engage PPAR signaling in the postnatal heart. Our findings support a model in which Coq10a-dependent UQ partitioning influences PPAR-associated signaling during postnatal cardiac growth.

## Results

### Identification of Uncharacterized Mitochondrial Proteins that Are Tissue-Enriched and Postnatally Upregulated.

**Fig. 1.**
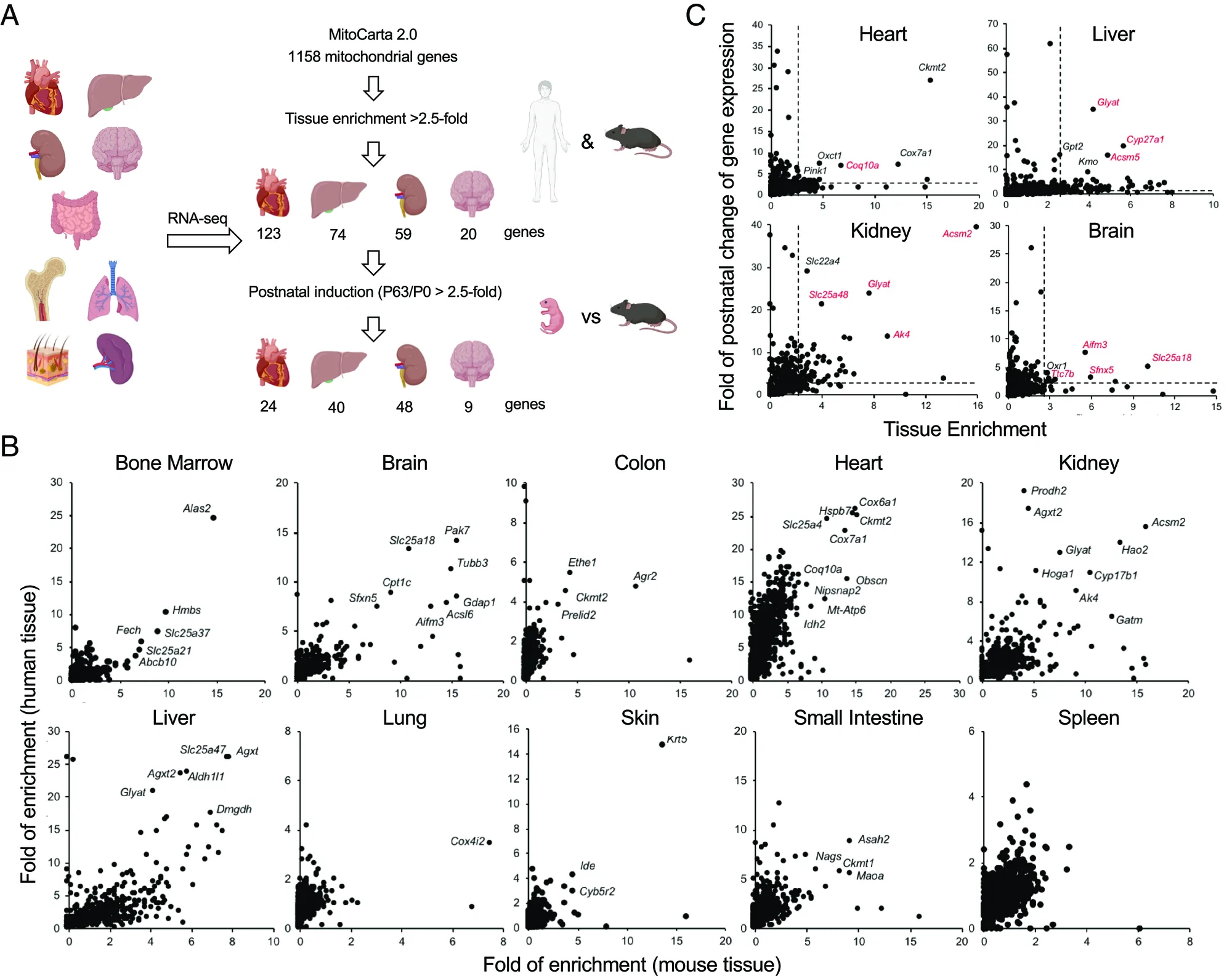
Identification of human and mouse organ-enriched, postnatally upregulated genes encoding mitochondrial proteins. (*A*) Strategy for identifying organ-enriched mitochondrial protein-encoding genes with poorly characterized physiological functions. (*B*) Mitochondrial protein-encoding gene expression across ten mouse and human organs, expressed relative to the mean across organs. (*C*) Postnatal expression changes in tissue-enriched mitochondrial protein-encoding genes. The five most upregulated tissue-enriched mitochondrial genes for each organ are labeled. The genes without reported knockout mice are shown in red. Dashed lines denote 2.5-fold cutoffs. Tissue enrichment represents the lower enrichment score between mouse and human, and postnatal change represents expression at postnatal day 63 relative to postnatal day 0.

To identify mammalian tissue-specific mitochondrial proteins whose physiological functions have not been reported in knockout mice, we first analyzed all 1,158 genes in MitoCarta2.0 that encode proteins with strong evidence of mitochondrial localization (19). The tissue enrichment score was next determined by analyzing gene expression across 10 organs (bone marrow, brain, colon, heart, kidney, liver, lung, skin, small intestine, and spleen) in both mice and humans using two previous RNA-seq analyses (20, 21) (Fig. 1*A* and Dataset S1). These analyses revealed the heart, liver, kidney, and brain as the top four organs with the highest numbers of tissue-enriched mitochondrial protein-encoding genes (123, 74, 59, 20 genes respectively if a 2.5-fold enrichment was taken as a cutoff for both mouse and human tissues), followed by the small intestine, bone marrow, colon, skin, lung, and spleen with 11, 10, 4, 4, 1, and 0 tissue-enriched mitochondrial genes, respectively (Fig. 1*B*).

Many mammalian organs increase mitochondrial gene expression during postnatal mitochondrial biogenesis. Thus, we analyzed the postnatal expression changes of tissue-enriched genes in the mouse heart, liver, kidney, and brain (22), and found 24, 40, 48, and 9 genes whose expression levels are at least 2.5-fold enriched and 2.5-fold upregulated after birth in these four organs, respectively (Fig. 1*C*). At the time of our initial screen, 52 of these genes lacked reported knockout mouse phenotypes or had poorly defined physiological functions in vivo (Dataset S2). *Coq10a* was the top candidate in the heart, with 6.8-fold tissue enrichment and 6.9-fold postnatal increase (Fig. 1*C*).

### Coq10a Is a Striated Muscle-Enriched Mitochondrial Protein Induced During Postnatal Heart Maturation.

**Fig. 2.**
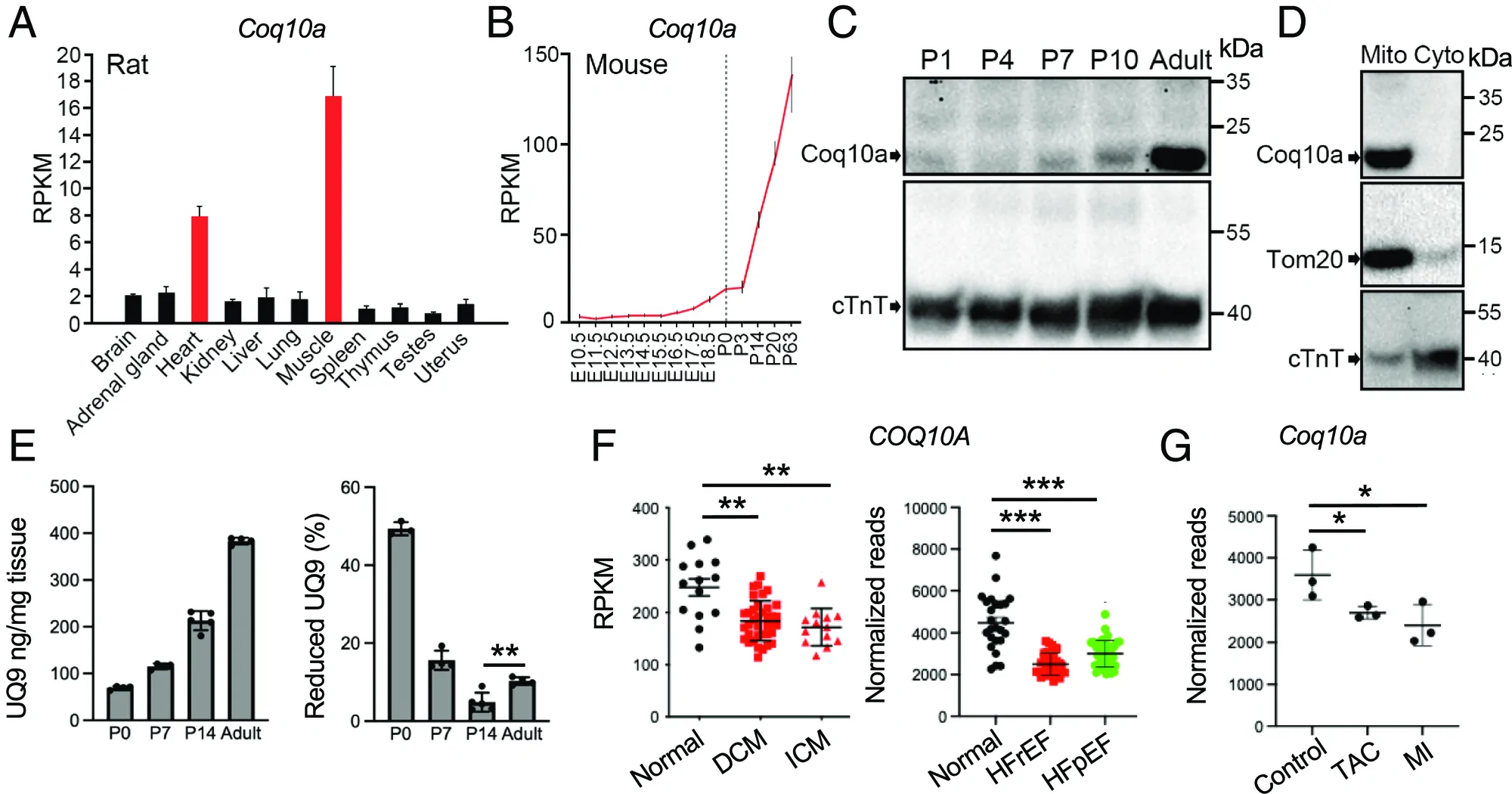
Coq10a is enriched in postnatal striated muscles and downregulated in heart failure. (*A*) *Coq10a*expression across adult rat organs based on the transcriptomic BodyMap (n = 8). (*B*) Temporal *Coq10a* expression from embryonic day (E) 10.5 to postnatal day (P) 63 in the mouse heart (n = 4). (*C*) Coq10a protein expression in P1, P4, P7, P10, and adult mouse hearts. Cardiac troponin T (cTnT) was used as a loading control. (*D*) Localization of Coq10a protein in the mitochondrial (Mito) but not cytosolic (Cyto) fraction of adult mouse heart lysates. Tom20 was used as a mitochondrial marker, and cTnT was used as a cytoplasmic fraction marker. (*E*) Total Ubiquinone-9 (UQ9) level (*P* < 0.0001 by one-way ANOVA) and the percentage of reduced UQ9 level (*P* < 0.0001 by one-way ANOVA) per total UQ9 in P0, P7, P14, and adult ventricles. (*F*) *COQ10A* expression in human pathological hearts including dilated cardiomyopathy (DCM), ischemic cardiomyopathy (ICM) (normal, n = 14; DCM, n = 37; ICM, n = 13), heart failure with reduced ejection fraction (HFrEF) and heart failure with preserved ejection fraction (HFpEF) (normal, n = 25; HFrEF, n = 33; HFpEF n = 41). (*G*) *Coq10a* expression in mouse models of heart failure including transverse aortic constriction (TAC) and myocardial infarction (MI) (n = 3). Values are reported as mean ± SEM. *P < 0.05, **P < 0.01, ***P < 0.001.

To investigate the expression, regulation, and function of *Coq10a* comprehensively, we first analyzed a rat RNA-seq transcriptomic BodyMap across 11 organs (23) and revealed that *Coq10a* is predominantly expressed in the skeletal muscle and heart at 21 wk of age (Fig. 2*A*) while its paralog *Coq10b* is highly expressed in the adrenal gland but present in most other organs at a low level (*SI Appendix*, Fig. S1*A*). In addition, a recent detailed analysis of gene expression across mouse organ development showed that cardiac expression of *Coq10a* begins around E15.5, rapidly increases postnatally and keeps increasing by postnatal day (P) 63 (Fig. 2*B*); *Coq10b* expression follows a similar temporal pattern in the heart (*SI Appendix*, Fig. S1*B*) (22). In the adult mouse heart, *Coq10a* expression is four- to fivefold higher than *Coq10b* (Fig. 2*B* and *SI Appendix*, Fig. S1*B*). Consistently, we found that Coq10a protein levels increase in the mouse heart during postnatal development (Fig. 2*C*). Because yeast Coq10p protein localizes to the inner mitochondrial membrane (9, 11), we investigated whether mouse Coq10a is also a mitochondrial protein. Subcellular fractionation of the adult mouse ventricular tissue indeed showed that Coq10a is highly enriched in the mitochondrial fraction rather than the cytosolic fraction (Fig. 2*D*). Altogether, these results support Coq10a as a striated-muscle enriched mitochondrial protein. To further characterize postnatal changes in the ubiquinone pool in the heart, we quantified ventricular ubiquinone-9 (UQ9), a predominant form of UQ in rodents, by high-performance liquid chromatography (HPLC) at P0, P7, P14, and adulthood (Fig. 2*E*). Total UQ9 abundance increased progressively during postnatal development, whereas the proportion of reduced UQ9 relative to total UQ9 decreased from P0 to P14 and then partially increased in adulthood (Fig. 2*E*). Notably, these developmental changes in cardiac UQ9 coincided with the postnatal increase in *Coq10a* gene expression and protein levels (Fig. 2 *B* and *C*), raising the possibility that Coq10a contributes to the regulation of UQ homeostasis during postnatal heart maturation. We next examined whether *Coq10a* expression is altered in pathological hearts. In a publicly available human left ventricular RNA-seq dataset from the Gene Expression Omnibus (GEO; GSE116250), *COQ10A* expression was significantly reduced in diseased hearts, including those with dilated cardiomyopathy (DCM), ischemic cardiomyopathy (ICM), heart failure with reduced ejection fraction (HFrEF), and heart failure with preserved ejection fraction (HFpEF) (Fig. 2*F*). Similarly, reanalysis of dataset GSE96566 indicated that *Coq10a* expression was significantly decreased in mouse pathological hearts following transverse aortic constriction (TAC) or myocardial infarction (MI) (Fig. 2*G*). Collectively, these findings indicate that *Coq10a* is induced during normal postnatal heart maturation but downregulated in pathological cardiac remodeling.

### *Coq10a* Gene Expression Is Activated by Thyroid Hormone Receptor, Nkx2.5, and Tbx5.

**Fig. 3.**
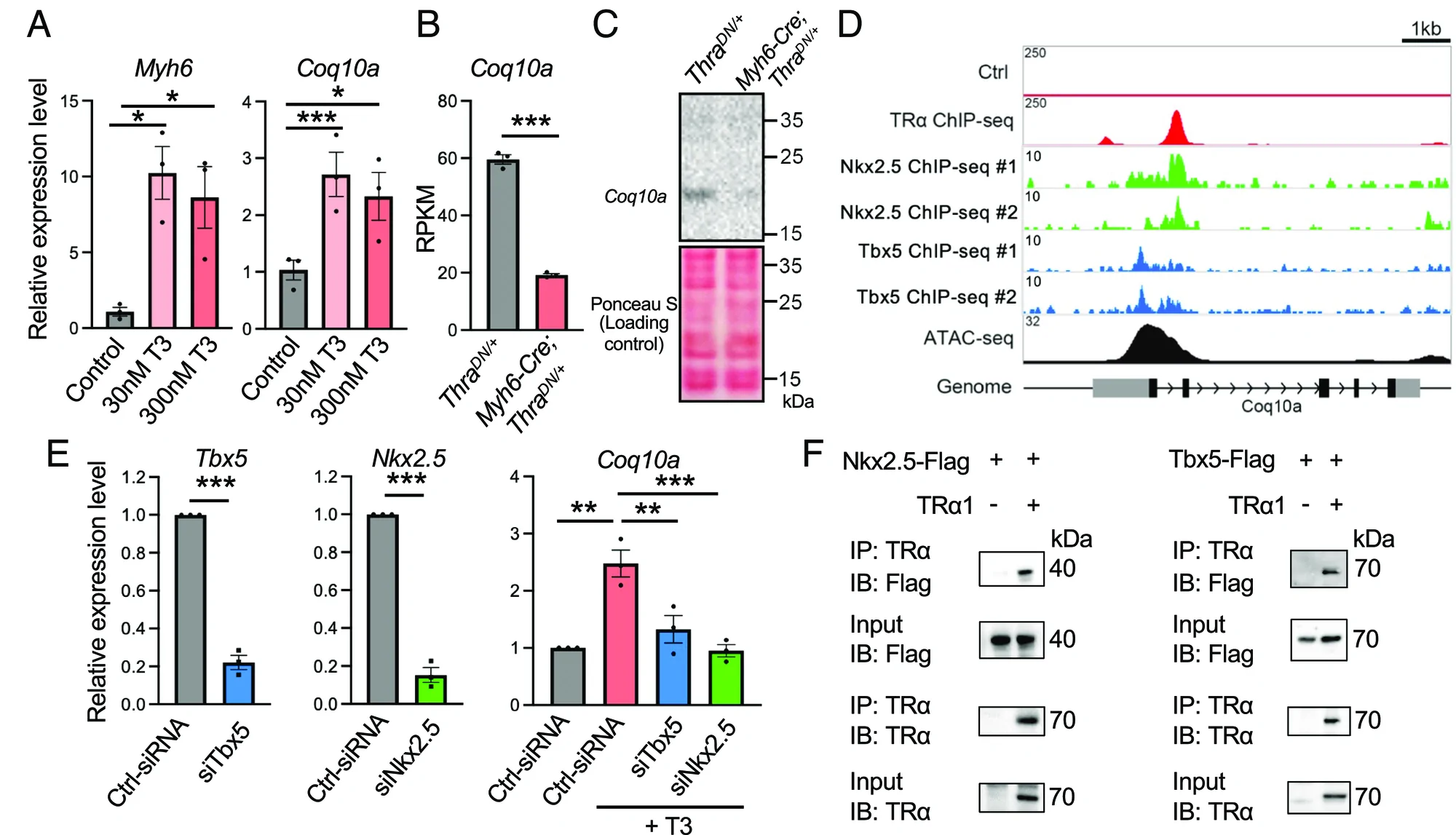
*Coq10a* expression is regulated by thyroid hormone receptor, Nkx2.5, and Tbx5 in the heart. (*A*) Reverse transcription coupled quantitative PCR (RT-qPCR) analysis of *Coq10a* and *Myh6* expression in triiodothyronine (T3)-treated neonatal mouse cardiomyocytes in culture (n = 3). (*B*) RNA-seq analysis of *Coq10a* gene expression in the postnatal day (P) 14 hearts of cardiac-specific thyroid hormone receptor α dominant negative mutant (*Myh6-Cre;Thra^DN/+^*) and littermate control (*Thra^DN/+^*) mice (n = 3). (*C*) Coq10a protein in *Myh6-Cre; Thra^DN/+^* and control hearts at P14. (*D*) TRα, Nkx2.5, and Tbx5 binding sites based on chromatin immunoprecipitation sequencing (ChIP-seq), together with open chromatin regions identified by assay for transposase-accessible chromatin sequencing (ATAC-seq). (*E*) RT-qPCR analysis of T3-induced *Coq10a* expression following *Nkx2.5* and *Tbx5* knockdown in neonatal mouse cardiomyocytes (n = 3). Data were analyzed by one-way ANOVA followed by Sidak’s multiple comparisons test for selected comparisons. (*F*) Coimmunoprecipitation of TRα with Nkx2.5 and Tbx5. Values are reported as mean ± SEM. *P < 0.05, **P < 0.01, ***P < 0.001.

Because the temporal pattern of *Coq10a* expression matches that of circulating thyroid hormones (24) and expression of downstream target genes such as *Myh6* (25), we hypothesized that *Coq10a* gene expression is controlled by thyroid hormone signaling in the heart. Thyroid hormone receptors are transcription factors that bind DNA directly and regulate the expression of target genes. We first tested whether increase of thyroid hormones is sufficient to activate *Coq10a* expression in primary mouse neonatal cardiomyocytes. Supplementation of culture media with triiodothyronine (T3) enhanced the gene expression of *Coq10a* by ~2.5-fold in primary cardiomyocytes, similar to the T3-induced upregulation of *Myh6* (Fig. 3*A*). Thus, these results suggest that activation of thyroid hormone signaling induces *Coq10a* gene expression. To test whether thyroid hormone signaling is necessary for *Coq10a* expression in the heart, we analyzed its transcript levels in mice that express a dominant negative form of thyroid hormone receptor alpha (*Thra^DN^*) to suppress thyroid hormone signaling specifically in cardiomyocytes (*Myh6-Cre;Thra^DN/+^*) (26). RNA-seq analyses of *Myh6-Cre;Thra^DN/+^* hearts at P14 demonstrated that the gene expression of *Coq10a* was reduced to one-third of that in control hearts (Fig. 3*B*). Consistently, Coq10a protein was also reduced in *Myh6-Cre;Thra^DN/+^* hearts at P14, compared to the littermate control (Fig. 3*C*). Furthermore, our chromatin immunoprecipitation-sequencing (ChIP-seq) analysis revealed that thyroid hormone receptor α binding sites localize on the first intron of *Coq10a* (26), which closely overlap with open chromatin region based on Assay for Transposase-Accessible Chromatin sequencing (ATAC-seq) analysis of adult mouse hearts (Fig. 3*D*) (27), providing strong support for *Coq10a* as a direct target gene of thyroid hormone receptor α.

Since thyroid hormone receptor expression is quite ubiquitous, why is *Coq10a* only expressed in striated muscles at high levels? To identify cardiac transcription factors that may control the cardiac expression of *Coq10a*, we identified in a previously published ChIP-seq analysis that Nkx2.5 and Tbx5 bind adjacently to the thyroid hormone receptor α binding site in the HL-1 myocyte cell line (28) (Fig. 3*D*). To test whether Nkx2.5 and Tbx5 are also involved in the regulation of *Coq10a* expression, we knocked down these two genes individually in neonatal mouse cardiomyocytes in culture (Fig. 3*E*). Consequently, the upregulation of the *Coq10a* gene expression by thyroid hormone treatment was mitigated by *Nkx2.5* and *Tbx5* depletion (Fig. 3*E*), suggesting Nkx2.5 and Tbx5 are required for the control of *Coq10a* expression in response to thyroid hormone stimulation. To further examine the relationship between these transcription factors and TRα, we performed coimmunoprecipitation analysis and found that TRα physically associates with both Nkx2.5 and Tbx5 (Fig. 3*F*), supporting a model in which cardiac-enriched transcription factors, Nkx2.5 and Tbx5, cooperate with thyroid hormone signaling to drive *Coq10a* expression.

### Mice Lacking *Coq10a* Develop Cardiac Hypertrophy During the First 2 wk of Postnatal Life.

**Fig. 4.**
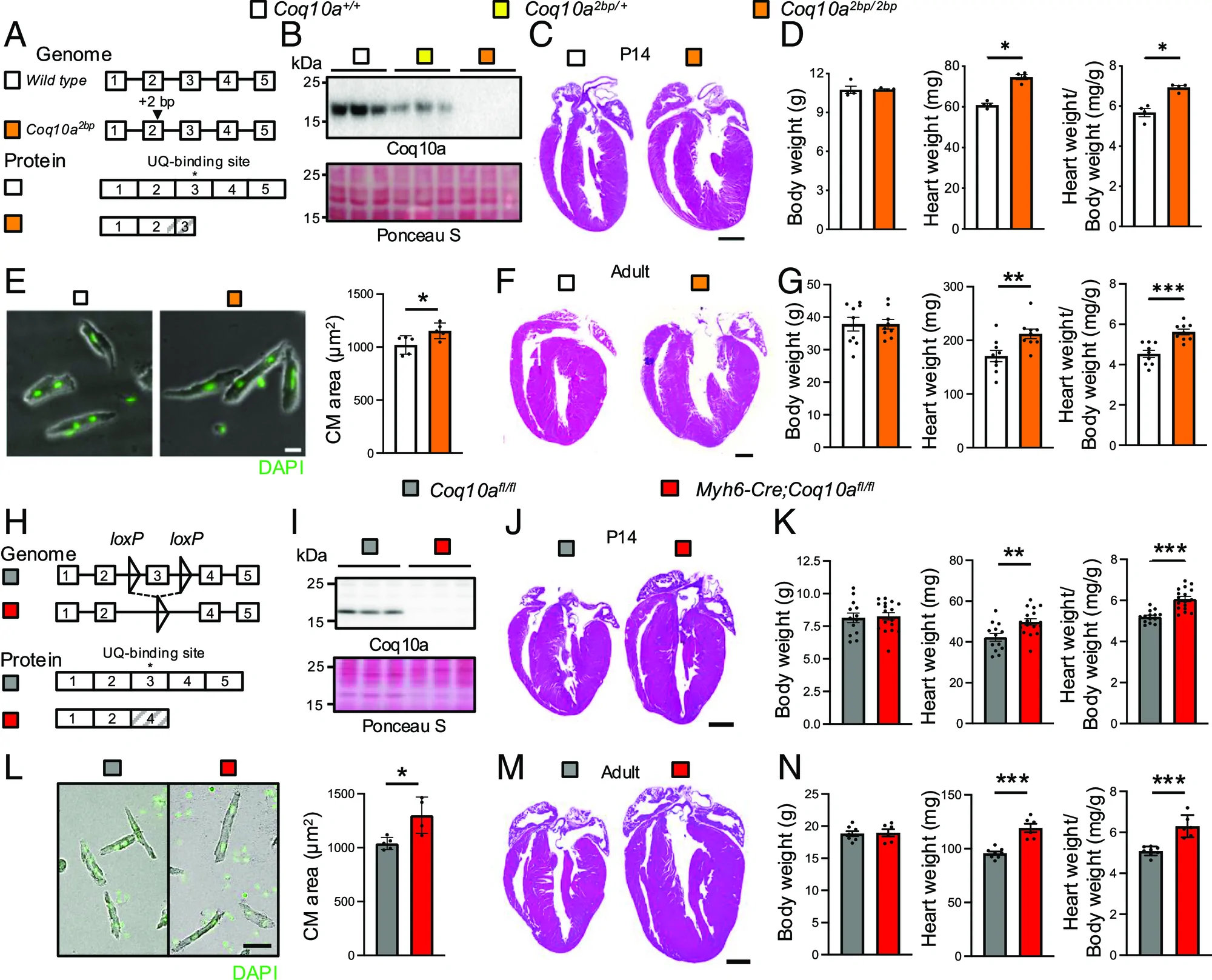
Global and cardiomyocyte-specific *Coq10a* knockout mice exhibit cardiac hypertrophy. (*A*) Schematic for generating *Coq10a^2bp/2bp^* knockout mice. The sgRNA was designed to target a sequence in the second exon, and the mutant mice carry a two-base pair (2 bp) insertion that results in a frameshift mutation in the ICR mouse strain background. The 2-bp insertion is predicted to disrupt the *Coq10a* coding sequence upstream of the UQ-binding site. (*B*) Western blot analysis of Coq10a protein in adult *Coq10a^+/+^*, *Coq10a^2bp/+^*, and *Coq10a^2bp/2bp^* hearts. (*C*) Hematoxylin-eosin (H&E)-stained postnatal day (P) 14 *Coq10a^+/+^* and *Coq10a^2bp/2bp^* hearts. (*D*) Body weight (BW), heart weight (HW), and HW/BW at P14 (n = 4 animals). (*E*) Isolated P14 cardiomyocytes (CMs) and cell area (n = 5 animals). (*F*) H&E-stained adult *Coq10a^+/+^* and *Coq10a^2bp/2bp^* hearts. (*G*) BW, HW, and HW/BW in adults (n = 9 animals). (*H*) *Coq10a* conditional allele and Cre-mediated exon 3 deletion. (*I*) Western blot analysis of Coq10a protein in adult *Coq10a^fl/fl^* and *Myh6-Cre;Coq10a^fl/fl^* hearts (n = 3 animals). (*J*) H&E-stained P14 *Coq10a^fl/fl^* and *Myh6-Cre;Coq10a^fl/fl^* hearts. (*K*) BW, HW, and HW/BW at P14 (*Coq10a^fl/fl^*, n = 13 animals; *Myh6-Cre;Coq10a^fl/fl^*, n = 17 animals). (*L*) Isolated CMs from P14 hearts and cell area (*Coq10a^fl/fl^*, n = 5 animals and *Myh6-Cre;Coq10a^fl/fl^*, n = 4 animals). (*M*) H&E-stained adult *Coq10a^fl/fl^* and *Myh6-Cre;Coq10a^fl/fl^* hearts. (*N*) BW, HW, and HW/BW in adults (*Coq10a^fl/fl^*, n = 8 animals; *Myh6-Cre;Coq10a^fl/fl^,* n = 6 animals). Values are reported as mean ± SEM. *P < 0.05, **P < 0.01, ***P < 0.001. [Scale bars, 1 mm (*C*, *F*, *J*, and *M*), 20 μm (*E*), 50 μm (*L*).]

To study the function of *Coq10a* in vivo, we generated *Coq10a* knockout mice on a CD1 genetic background. In fission yeast Coq10p, Phe39-Lys45 have been mapped as key UQ-binding residues (*SI Appendix*, Fig. S2*A*) (12). In this sequence, Phe39 and Pro41 are two highly conserved amino acids from bacteria to humans (*SI Appendix*, Fig. S2*A*) and have been shown by site-directed mutagenesis to be essential for UQ interaction (12). Thus, we exploited the CRISPR/Cas9-based genome-editing technique and designed a single-guide RNA (sgRNA) targeting a site upstream of the conserved ubiquinone-binding site (Fig. 4*A*). Sequencing of one stable line of mutant mice at the F2 generation revealed an insertion of two nucleotides at the targeted site, resulting in frameshift mutations, which produced a truncated Coq10a protein lacking the UQ-binding site (Fig. 4*A*). Coq10a protein expression was decreased in the *Coq10a^+/2bp^* heterozygous heart and was undetectable in the heart of adult *Coq10a^2bp/2bp^* homozygous mutant mice (Fig. 4*B* and *SI Appendix*, Fig. S2*B*).

*Coq10a^2bp/2bp^* mice are born at Mendelian ratio with normal gross morphology and fertility. We first evaluated heart phenotypes at P14 when most cardiomyocytes have withdrawn from the cell cycle. *Coq10a^2bp/2bp^* mice exhibited a 22% increase in the heart weight-to-body weight ratio despite comparable body weight (Fig. 4 *C* and *D*). This phenotype was further confirmed in seven F0 *Coq10a* homozygous mutant mice at P14 (*SI Appendix*, Fig. S2*C*). Although gene editing using the CRISPR/Cas9 system might cause off-target effects, the highly reproducible cardiomegaly observed in F2 and multiple F0 mutant animals suggests that this phenotype is a specific consequence of *Coq10a* loss of function. In addition, a similar but less pronounced increase was also observed in *Coq10a^+/2bp^* heterozygous mice, which displayed an approximately 13% elevation in the heart weight-to-body weight ratio relative to littermate controls (*SI Appendix*, Fig. S2*D*). To define the developmental timing of this hypertrophic growth, we measured body weight, heart weight, and the heart weight-to-body weight ratio at P1, P5, and P10 (*SI Appendix*, Fig. S3), in addition to P14 and adulthood (Fig. 4 *D*, *F*, and *G*). No differences were detected at P1 or P5 (*SI Appendix*, Fig. S3 *A* and *B*). By contrast, the heart weight-to-body weight ratio was increased by 16% at P10 and further rose to 22% at P14 and 25% in adulthood (Fig. 4 *D* and *G* and *SI Appendix*, Fig. S3C). Similar cardiac phenotypes were observed when heart size was normalized to tibia length at P14 and adulthood (*SI Appendix*, Fig. S3 *D* and *E*). These findings indicate that cardiac hypertrophy emerges during early postnatal development, within the first 2 wk after birth.

To determine whether the increased heart size is due to cardiomyocyte hypertrophy or hyperplasia, we first assessed the size of individual cardiomyocytes isolated from ventricles of P14 *Coq10a^2bp/2bp^* mice. Cardiomyocyte size was significantly increased in *Coq10a^2bp/2bp^* mice (Fig. 4*E*). Consistently, cardiomyocyte cross-sectional area was also increased in mutant hearts (*SI Appendix*, Fig. S4*A*). Next, to evaluate a possible contribution of hyperplasia to the cardiomegaly phenotype, we evaluated cardiomyocyte number, nucleation, proliferation, and apoptosis in *Coq10a^+/+^* and *Coq10a^2bp/2bp^* mice at P14. Analyses of *Coq10a^+/+^* and *Coq10a^2bp/2bp^* mice showed comparable total cardiomyocyte numbers in the ventricle (*SI Appendix*, Fig. S4*B*) and cardiomyocyte nucleation distribution (*SI Appendix*, Fig. S4*C*). Proliferating cardiomyocytes were assessed by immunostaining against cardiomyocyte nuclear marker pericentriolar material 1 (PCM1) and a proliferation marker Ki67. However, we observed no difference in proliferating cardiomyocytes between *Coq10a^+/+^* and *Coq10a^2bp/2bp^* mice (*SI Appendix*, Fig. S4*D*). To assess the extent of cell apoptosis, terminal deoxynucleotidyl transferase-mediated deoxyuridine triphosphate nick end labeling (TUNEL) assays were performed on histological sections and no difference in the number of TUNEL-positive cardiomyocytes was observed between *Coq10a^+/+^* and *Coq10a^2bp/2bp^* mice (*SI Appendix*, Fig. S4*E*). Therefore, our investigation suggests that the cardiomegaly is primarily due to an increase in cardiomyocyte size, which is indicative of cardiac hypertrophy. To further characterize the nature of this hypertrophic response at P14, we examined markers associated with pathological cardiac remodeling by RT-qPCR. *Nppa* expression was reduced, whereas *Nppb, Myh7, Myh6, Acta1, Col1a1, and Col3a1* expression was unchanged (*SI Appendix*, Fig. S4*F*). These findings do not support activation of a canonical pathological hypertrophic gene program at P14.

To minimize allele-specific effects, we generated an independent whole-open reading frame (ORF) *Coq10a* deletion line on C57BL/6N background (*SI Appendix*, Fig. S5*A*). Coq10a protein was undetectable in knockout hearts (*SI Appendix*, Fig. S5*B*). We refer to this strain as the *Coq10a^ORFdel/ORFdel^* line. At P14, while *Coq10a^ORFdel/ORFdel^* pups exhibited a reduction in body weight, the heart weight-to-body weight ratio was increased by 29% (*SI Appendix*, Fig. S5*C*). In adulthood, the growth delay was no longer apparent, and body weight had caught up to controls. However, the heart weight-to-body weight ratio remained elevated by 22% (*SI Appendix*, Fig. S5 *D* and *E*). Measurement of cardiomyocyte cross-sectional area also showed increased cardiomyocyte size (*SI Appendix*, Fig. S5*F*), whereas Sirius Red staining showed no increase in fibrosis in adult *Coq10a^ORFdel/ORFdel^* hearts (*SI Appendix*, Fig. S5*G*). Overall, these findings indicate persistent cardiac hypertrophy without overt fibrosis, comparable to the phenotype observed in the *Coq10a^2bp/2bp^* line (Fig. 4 *A*–*G*).

To further validate the cardiac contribution of *Coq10a* deletion, we generated a postnatal, cardiomyocyte-specific knockout model by crossing *Coq10a^fl/fl^* mice, which had been generated in B6D2 fertilized eggs and subsequently backcrossed onto the C57BL/6N background, with the *Myh6-Cre* driver (*Myh6-Cre;Coq10a^fl/fl^*). The conditional allele was designed such that Cre-mediated recombination excised exon 3 (Fig. 4*H*). Coq10a protein and mRNA were undetectable in the heart (Fig. 4*I* and *SI Appendix*, Fig. S6). At P14, *Myh6-Cre;Coq10a^fl/fl^* mice did not exhibit growth retardation, and the heart weight-to-body weight ratio was increased by 17% (Fig. 4 *J* and *K*), accompanied by an increase in isolated cardiomyocyte size (Fig. 4*L*). This increase persisted into adulthood, with a 24% elevation in the heart weight-to-body weight ratio (Fig. 4 *M* and *N*), supporting the conclusion that loss of *Coq10a* in cardiomyocytes contributes to the cardiac hypertrophy phenotype.

To test whether *Coq10a* deficiency is sufficient to induce cardiac hypertrophy in adulthood when physiological postnatal cardiac growth is largely completed, we conditionally deleted *Coq10a* in adult *Coq10a^fl/fl^* mice by systemic delivery of AAV9-Tnnt2-Cre-EGFP (*SI Appendix*, Fig. S7*A*). As a positive control for this AAV-mediated approach during the early postnatal period, we delivered the same virus at P1 and analyzed the hearts at P14. In this setting, Cre-EGFP expression was readily detected in the heart (*SI Appendix*, Fig. S7*B*), and the heart size, heart weight-to-body weight ratio, and the heart weight-to-tibia length ratio were increased (*SI Appendix*, Fig. S7 *C* and *D*), consistent with the genetic cardiomyocyte-specific knockout model (Fig. 4*K*). We then analyzed adult-injected mice 2 wk after viral delivery (*SI Appendix*, Fig. S7*E*). Cre-EGFP expression was robust in adult hearts (*SI Appendix*, Fig. S7*F*), and *Coq10a* mRNA levels were reduced by 76% as assessed by RT-qPCR (*SI Appendix*, Fig. S7*G*). However, in contrast to deletion during early postnatal development, adult-onset *Coq10a* deletion did not increase the heart weight-to-body weight ratio or the heart weight-to-tibia length ratio (*SI Appendix*, Fig. S7*H*). These data indicate that the cardiac hypertrophic response to *Coq10a* loss is pronounced during the early postnatal period and not in the adult.

### *Coq10a* Regulates Intracellular UQ Partitioning.

**Fig. 5.**
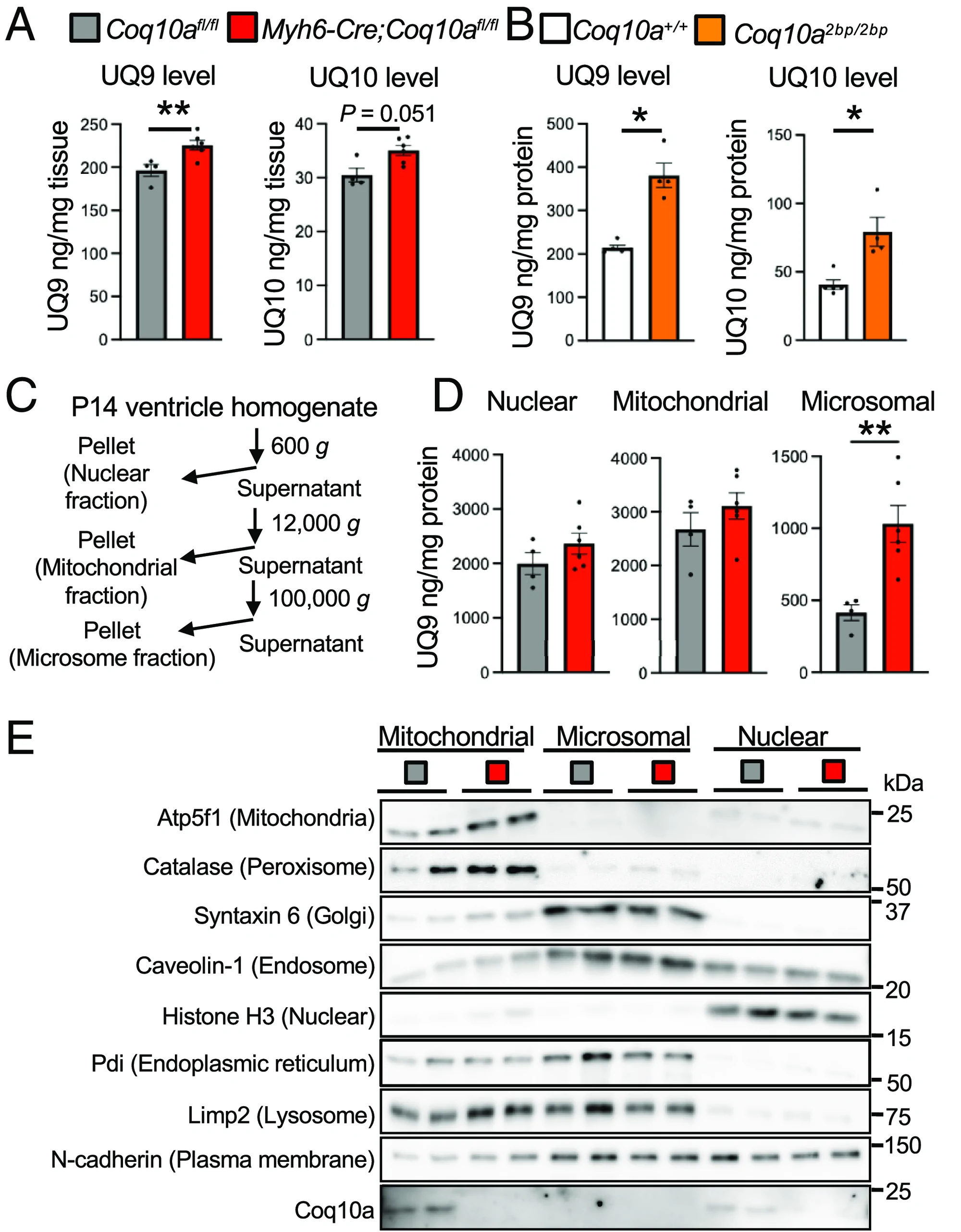
Coq10a controls subcellular ubiquinone distribution in the heart. (*A*) Quantitation of ubiquinone-9 (UQ9) and ubiquinone-10 (UQ10) levels in Cre-negative littermate control and *Myh6-Cre;Coq10a^fl/fl^* ventricles at postnatal day (P) 14 (*Coq10a^fl/fl^*, n = 4 animals; *Myh6-Cre;Coq10a^fl/fl^*, n = 6 animals). (*B*) UQ9 and UQ10 levels in *Coq10a^+/+^* and *Coq10a^2bp/2bp^* ventricles at P14 (*Coq10a^+/+^*, n = 5 animals; *Coq10a^2bp/2bp^*, n = 4 animals). (*C*) Heart fractionation workflow. (*D*) HPLC-based measurement of UQ9 levels in nuclear, mitochondrial, and microsomal fractions (*Coq10a^fl/fl^*, n = 4 animals; *Myh6-Cre;Coq10a^fl/fl^*, n = 6 animals). (*E*) Immunoblot analysis of organelle marker in subcellular fractions. Values are reported as mean ± SEM. *P < 0.05, **P < 0.01.

In yeast, Coq10p is a UQ-binding protein and has been proposed to facilitate UQ utilization, potentially by influencing its localization within the cell. We therefore asked whether *Coq10a* loss alters UQ abundance or distribution in the mammalian heart. We first quantified total UQ9 in the ventricular tissue of *Myh6-Cre;Coq10a^fl/fl^* and *Coq10a^fl/fl^* littermate control hearts. We found that UQ9 and UQ10 abundance was increased in KO ventricles (Fig. 5*A*). The same trend was observed in *Coq10a^2bp/2bp^* ventricles (Fig. 5*B*). In addition, the fractions of reduced UQ9 and UQ10 (ubiquinol) were also elevated in KO hearts (*SI Appendix*, Fig. S8). Notably, this increase was even more pronounced in isolated mitochondria (*SI Appendix*, Fig. S8). To determine whether *Coq10a* loss alters the intracellular distribution of UQ in cardiomyocytes, we fractionated ventricular homogenates from *Coq10a^fl/fl^* and *Myh6-Cre;Coq10a^fl/fl^* ventricles by differential centrifugation into three fractions (mitochondrial, microsomal, and nuclear) (Fig. 5*C*) and measured UQ levels in each fraction (Fig. 5*D*). The composition of each fraction was assessed by immunoblotting using ATP5F1 (mitochondria), Histone H3 (nucleus), and organelle markers including Catalase (peroxisome), Syntaxin 6 (Golgi), Caveolin-1 (endosome), PDI (endoplasmic reticulum, ER), Limp2 (lysosome), and N-cadherin (plasma membrane) (Fig. 5*E*). These markers showed comparable fractionation patterns between *Coq10a^fl/fl^* and *Myh6-Cre;Coq10a^fl/fl^* ventricles, indicating that *Coq10a* deletion did not grossly alter the organelle composition of the fractions. Coq10a protein was enriched in the mitochondrial fraction of *Coq10a^fl/fl^* ventricles and was absent from *Myh6-Cre;Coq10a^fl/fl^* ventricles. Notably, UQ9 was increased in the microsomal fraction, a heterogeneous membrane fraction containing markers of the Golgi, endoplasmic reticulum, endosomes, and other nonmitochondrial membrane compartments, whereas UQ9 levels in the mitochondrial and nuclear fractions were not significantly altered (Fig. 5*D*). To independently assess whether Coq10a influences subcellular UQ localization in a cell-based system, we used H9C2 cells, a rat ventricular cardiomyoblast line derived from embryonic heart tissue, which were expected to provide a lower endogenous Coq10a background than postnatal cardiomyocytes. We compared *mCoq10a* overexpression with shRNA-mediated *Coq10a* knockdown (*SI Appendix*, Fig. S9*A*) and monitored the intracellular localization using a detectable artificial UQ probe following uptake (*SI Appendix*, Fig. S9 *B* and *C*) (29). *mCoq10a* overexpression promoted strong mitochondrial enrichment of the probe, whereas *Coq10a* knockdown redistributed the signal toward the nonmitochondrial regions (*SI Appendix*, Fig. S9*C*). Collectively, these results indicate that Coq10a contributes to intracellular UQ partitioning and localization, consistent with proposed chaperone-like roles of START-domain Coq10 proteins in UQ handling.

### *Coq10a* Deficiency Induces a PPAR-Responsive Metabolic Program, and PPARα/γ Inhibition Attenuates Cardiac Hypertrophy.

**Fig. 6.**
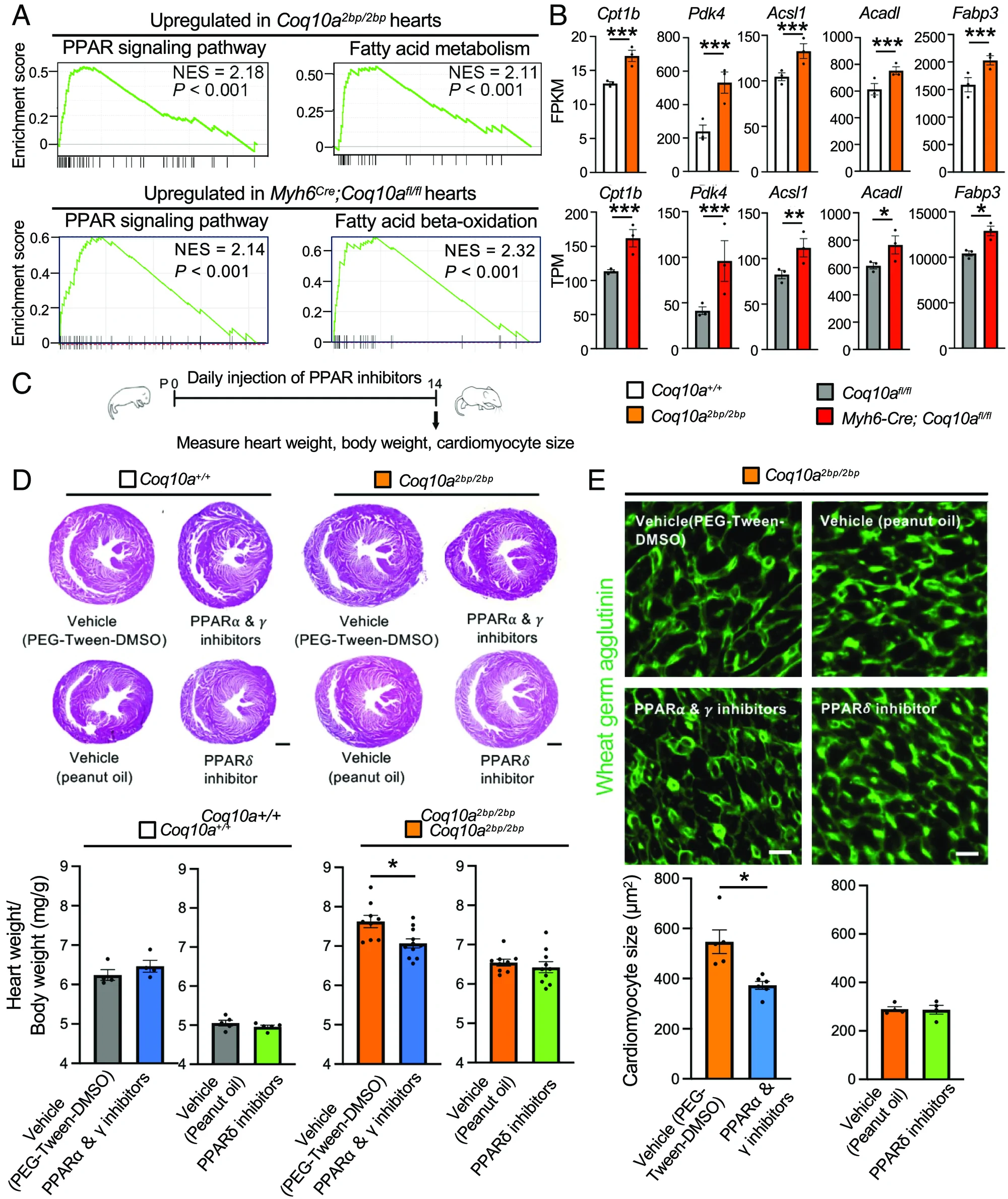
PPAR activation contributes to postnatal cardiac hypertrophy in *Coq10a*-deficient hearts. (*A*) RNA-seq pathway analysis of postnatal day (P) 14 *Coq10a^2bp/2bp^* and *Myh6-Cre;Coq10a^fl/fl^* hearts showing enrichment of PPAR signaling and fatty acid metabolism/β-oxidation (n = 3 animals). (*B*) Expression of PPAR-responsive genes in both models (n = 3 animals). Values are FPKM and TPM, respectively. (*C*) Experimental design for PPAR inhibition from P0 to P13 with analysis at P14. (*D*) Heart weight-to-body weight ratio and heart cross-sections following PPARα&γ or PPARδ inhibition (*Coq10a^+/+^*, n = 4 to 5 animals; *Coq10a^2bp/2bp^*, n = 9 to 11animals). (*E*) Wheat germ agglutinin (WGA) staining and cardiomyocyte cross-sectional area in *Coq10a^2bp/2bp^* hearts following treatment with the indicated PPAR inhibitors at P14. (PPARα&γ inhibitors, n = 5 to 6 animals; PPARδ inhibitors, n = 4). Values are reported as mean ± SEM. *P < 0.05, **P < 0.01. ***P < 0.001.

RNA-seq analysis of P14 *Coq10a*^2bp/2bp^ and *Myh6-Cre;Coq10a*^fl/fl^ hearts revealed broad transcriptional changes in metabolic and cellular pathways (*SI Appendix*, Figs. S10 and S11 and Datasets S3 and S4). Gene ontology (GO analysis) identified multiple altered biological processes, while gene set enrichment analysis (GSEA) revealed PPAR signaling as a shared positively enriched pathway in both models, accompanied by enrichment of fatty acid metabolism or β-oxidation (Fig. 6*A*). The shared leading-edge genes underlying the PPAR signaling enrichment in both models were *Cpt1b, Pdk4, Acsl1, Acadl,* and *Fabp3*, which are PPAR-responsive genes involved in fatty acid handling and oxidative metabolism (30). None of these shared leading-edge genes is a canonical hypertrophic regulator. These genes were significantly increased in both *Coq10a^2bp/2bp^* and *Myh6-Cre;Coq10a^fl/fl^* hearts (Fig. 6*B* and *SI Appendix*, Figs. S10*C* and S11*C*). To further examine the developmental timing of this response, we performed RT-qPCR analysis of PPARα target genes in *Coq10a^+/+^* and *Coq10a^2bp/2bp^* hearts at P1, P5, P10, and adulthood, with *Coq10a* included as a positive control (*SI Appendix*, Fig. S10*D*). Among the genes examined, *Pdk4, Cpt1b, Cd36*, and *Acox1* were upregulated in *Coq10a^2bp/2bp^* hearts at P10, whereas comparable induction was not observed at P1, P5, or adulthood. These results suggest that increased expression of PPAR-responsive metabolic genes in *Coq10a*-deficient hearts is most evident during the early postnatal developmental window. Because PPARs regulate transcriptional programs involved in fatty acid uptake and β-oxidation, the concordant enrichment of PPAR signaling and the induction of established PPAR-responsive genes in both *Coq10a^2bp/2bp^* and *Myh6-Cre;Coq10a^fl/^*^fl^ hearts are consistent with enhanced PPAR-associated transcriptional activity following *Coq10a* loss. However, these transcriptomic changes do not identify the downstream growth-signaling mechanism responsible for cardiomyocyte enlargement.

Because UQ10 has been reported to modulate PI3K/AKT/mTOR signaling in a context-dependent manner (31, 32), we examined whether canonical mTORC1 signaling was altered in P14 *Coq10a^2bp/2bp^* hearts. Phosphorylation of p70S6K at Thr389, S6 at Ser240/244, and 4E-BP1 at Thr37/46 was assessed by western blotting. None of these mTORC1 readouts was increased. Rather, the phospho-p70S6K/total p70S6K ratio was significantly reduced, whereas the phospho-S6/total S6 and phospho-4E-BP1/total 4E-BP1 ratios were unchanged (*SI Appendix*, Fig. S12). These findings do not support activation of canonical mTORC1 signaling in P14 *Coq10a^2bp/2bp^* hearts.

Because we found that Coq10a expression is regulated by thyroid hormone signaling (Fig. 3), and thyroid hormone signaling promotes postnatal cardiomyocyte maturation and growth, we then examined whether *Coq10a* loss broadly altered thyroid hormone-dependent gene expression. We assessed a panel of thyroid hormone-responsive genes, including *Myh6, Atp2a2, Cpt2, Thrb, Art1, Itgb6, Hcn2, Csdc2, Ryr2, Kcnd2, Hr,* and *Klf9,* in both *Coq10a^2bp/2bp^* and *Myh6-Cre;Coq10a^fl/fl^* RNA-seq datasets. Although several genes showed altered expression in *Coq10a^2bp/2bp^* hearts, these changes were not consistently observed in *Myh6-Cre;Coq10a^fl/fl^* hearts (*SI Appendix*, Fig. S13). These data do not support a consistent alteration of thyroid hormone-dependent transcriptional programs as a shared response to *Coq10a* loss.

We also assessed the expression of UQ synthesis family genes in RNA-seq datasets from both *Coq10a^2bp/2bp^* and *Myh6-Cre;Coq10a^fl/fl^* hearts. *Coq3* and *Coq6* showed mild upregulation in *Coq10a^2bp/2bp^* hearts, whereas no significant changes were detected in the expression of the other genes (*SI Appendix*, Fig. S14). *Coq10b* expression was also unchanged, suggesting that compensatory rescue is unlikely to occur through transcriptional upregulation of the paralogue. These results suggest that neither the increase in ventricular UQ abundance nor the mild cardiac phenotype is likely to be explained by broad transcriptional activation of the UQ synthesis pathway or by transcriptional compensation through Coq10b.

Recently, UQ and its synthetic analogue idebenone were shown to physically bind PPAR proteins (α and γ but not δ) and function as a natural ligand and partial agonist (8). Intriguingly, the PPAR pathway is activated during physiological hypertrophy and inhibition of PPAR blocks Insulin-like growth factor I (IGF1)-induced cardiomyocyte hypertrophy (33). We therefore first examined the cardiac expression of all three PPAR members and found they were expressed at comparable levels (*SI Appendix*, Fig. S15*A*). In *Coq10a*-deficient hearts, UQ9 accumulated in the microsomal fraction (Fig. 5*D*). This raised the possibility that redistribution of UQ to a nonmitochondrial membrane-enriched fraction may contribute to the aberrant PPARα/γ signaling in vivo. To test this idea, we administered PPAR inhibitors daily from P0 through P13 to *Coq10a^+/+^* and *Coq10a^2bp/2bp^* mice and analyzed at P14 (Fig. 6*C*). Combined administration of the PPARα inhibitor GW6471 and the PPARγ inhibitor T0070907, but not the PPARδ inhibitor GSK3787, significantly reduced the heart weight-to-body weight ratio (Fig. 6*D*) and cardiomyocyte size (Fig. 6*E*). Together, these results support a functional role for PPARα/γ signaling in postnatal cardiac hypertrophic growth in *Coq10a*-deficient hearts, potentially in association with altered intracellular UQ partitioning.

### Structural Modeling and Mutagenesis Identify Determinants of Differential UQ Responsiveness Among PPAR Isoforms.

**Fig. 7.**
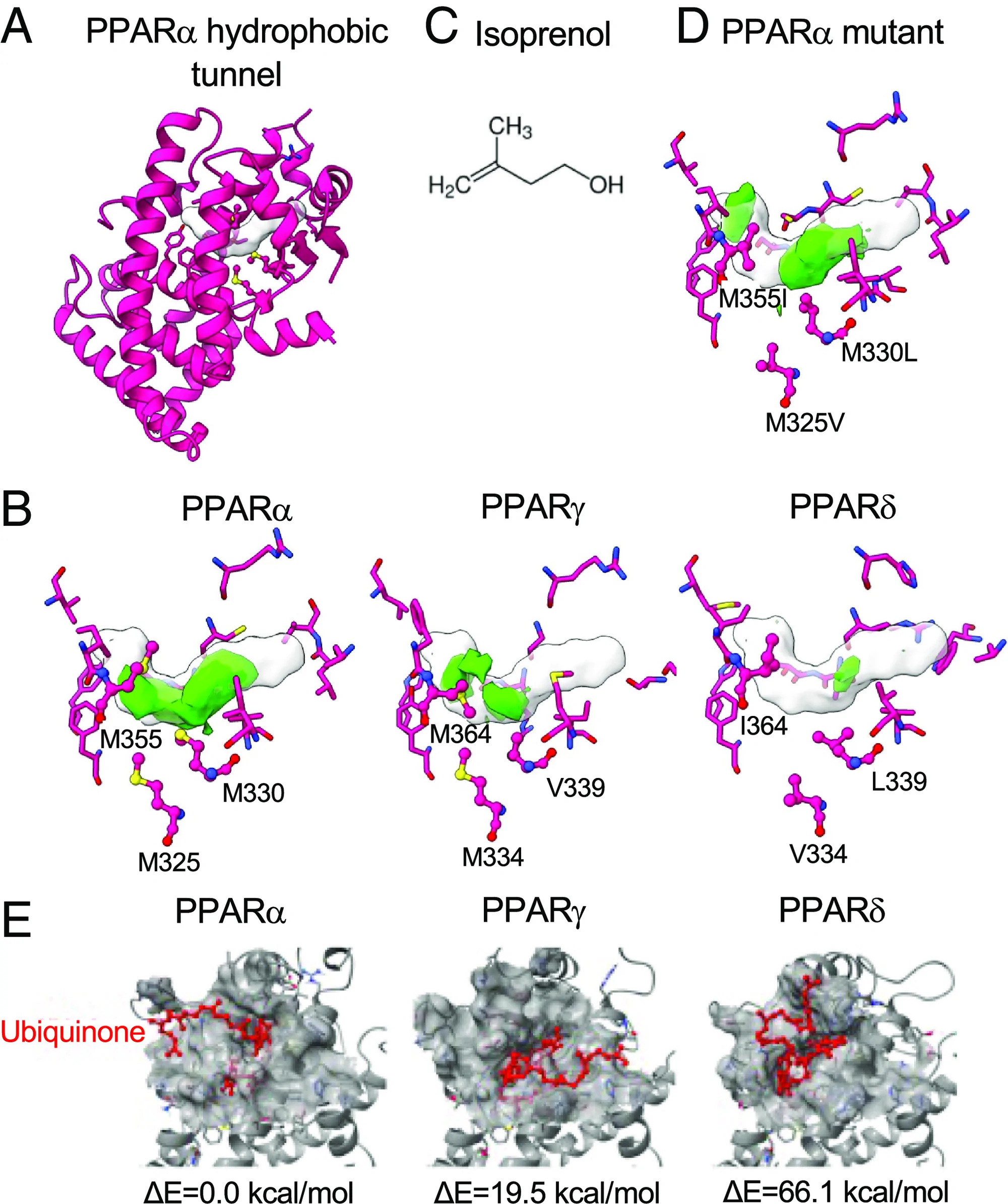
Structural modeling of differential ubiquinone responsiveness among PPAR isoforms. (*A*) Hydrophobic ligand-binding tunnel of the oleic acid-bound PPARα structure. The hydrophobic tunnel is shown as a light gray surface. (*B*) Isoprenol density maps from mixed-solvent molecular dynamics simulations of PPARα, PPARγ, and PPARδ. Residues differing among the three isoforms are shown, with isoprenol density represented as a green surface. (*C*) Structure of isoprenol used as the cosolvent probe. (*D*) Isoprenol density map of the PPARα M325V/M330L/M355I mutant. (*E*) Predicted UQ10 docking poses for PPARα, PPARγ, and PPARδ, and relative binding scores (ΔE) are indicated.

To investigate structural features that may contribute to differential UQ responsiveness among PPAR isoforms, we compared their ligand-binding pockets. PPARγ and PPARδ structures (PDB IDs: 5U5L and 2GWX, respectively) were aligned to the oleic acid-bound PPARα structure (PDB ID: 6LX8), revealing several amino acid differences surrounding the hydrophobic tunnel that accommodates fatty acid tails (Fig. 7 *A* and *B*). Mixed-solvent molecular dynamics simulations, a well-established method to characterize binding sites in proteins (34), using isoprenol as a probe of the UQ isoprenoid chain showed greater cosolvent occupancy within the hydrophobic tunnels of PPARα and PPARγ than PPARδ (Fig. 7 *B* and *C*).

Structure-based comparison identified M325, M330, and M355 in PPARα as corresponding to V334, L339, and I364 in PPARδ, whereas only the position corresponding to M330 differed in PPARγ (Fig. 7*B*). To test whether these substitutions contribute to differential isoprenoid-chain accommodation, we generated an in silico PPARα M325V/M330L/M355I mutant and repeated the isoprenol simulation. Isoprenol occupancy within the hydrophobic tunnel was reduced in the mutant, particularly near the position corresponding to M355 (Fig. 7*D*), suggesting that these hydrophobic residues contribute to differences in ligand accommodation among PPAR isoforms.

We next performed molecular docking to evaluate UQ10 binding. Ligand-binding sites were defined using p-benzoquinone density maps (*SI Appendix*, Fig. S15*B*), and UQ10 was docked to PPARα, PPARγ, and PPARδ (Fig. 7*E*). The predicted UQ10 binding modes in PPARα and PPARγ placed the benzoquinone head group within more solvent-sequestered pockets and the isoprenoid chain along hydrophobic channels, whereas the predicted PPARδ binding mode was more solvent-exposed. Consistently, relative binding scores were more favorable for PPARα and PPARγ than for PPARδ (Fig. 7*E*). Together, these analyses identify structural differences in the PPAR ligand-binding pockets that may contribute to differential responsiveness to UQ10.

**Fig. 8.**
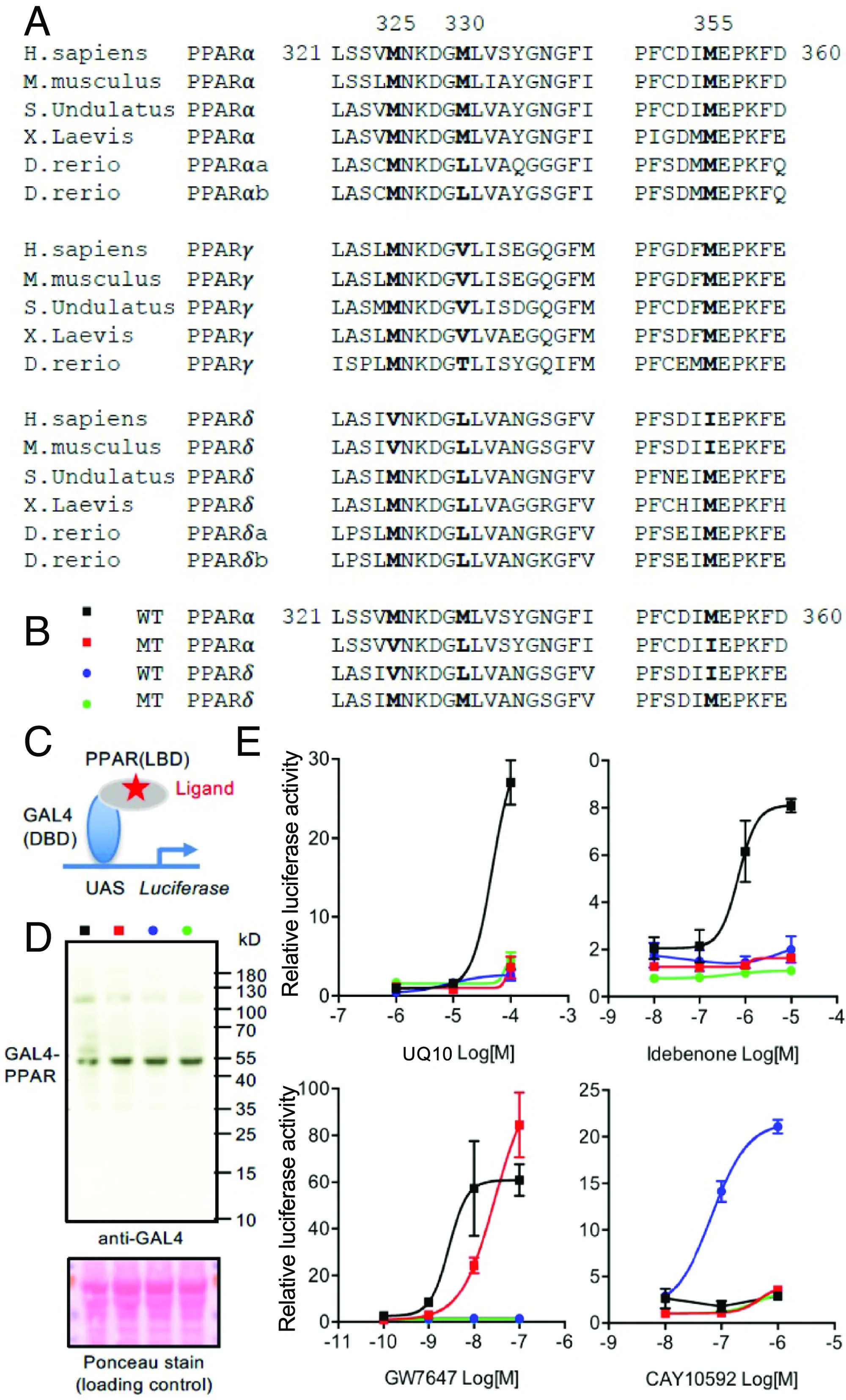
Three methionine residues account for the differing abilities of PPARα and PPARδ in UQ10- and idebenone-induced activation. (*A*) Sequence alignment of vertebrate PPARs. (*B*) WT and mutant (MT) PPAR constructs. (*C*) Functional examination using fusion proteins between Gal4 DNA-binding domain (DBD) and PPAR ligand binding domain as well as a UAS-luciferase reporter in HEK293T cells. (*D*) Confirmation of comparable expression of each fusion protein. (*E*) Dose–response curves of luciferase activity after treatment of different ligands for 24 h (n = 3). Values are reported as mean ± SEM.

The three PPARα residues implicated by structural modeling (Met325, Met330, and Met355) correspond to Val334, Leu339, and Ile364 in PPARδ (Fig. 7*B*). Sequence alignment revealed that vertebrate PPARα and PPARγ retain at least two methionine at these positions, whereas mammalian PPARδ lacks methionine at all three (Fig. 8*A*). Moreover, reptile, amphibian, and fish PPARδ proteins actually have two methionine residues in these three positions, suggesting that divergence between PPARδ and PPARα/γ at these three positions occurred later in evolution and could be a mammal-specific event. To test the functional importance of these three methionine residues, we first generated a PPARα mutant (MMM-VLI) as well as a complementary PPARδ mutant (VLI-MMM) with the amino acids at these three locations swapped (Fig. 8*B*). Next, we fused the ligand binding domain from wild-type (WT) and mutant PPARα and PPARδ with the DNA-binding domain of GAL4, which allowed us to determine PPAR ligand-induced UAS-driven luciferase reporter expression in HEK293T cells (Fig. 8*C*). All WT and mutant fusion proteins expressed at comparable levels (Fig. 8*D*). Using a range of doses of four PPAR agonists UQ10, idebenone, GW7647 (selective PPARα agonist), and CAY10592 (selective PPARδ agonist), we observed that the PPARα MMM-VLI mutant could not be activated by UQ10 or idebenone while it could still be activated by GW7647 (Fig. 8*E*). However, the PPARδ VLI-MMM mutant did not acquire responsiveness to UQ10 and idebenone (Fig. 8*E*). The VLI-MMM mutations also caused the loss of response to CAY10592 even though the mutant PPARδ was expressed at the same level as WT PPARδ (Fig. 8*E*), suggesting that the VLI three amino acids in PPARδ may be involved in CAY10592 binding. Nevertheless, these mutagenesis analyses provide strong support for our molecular docking model in which the specific amino acids in these three locations contribute to the difference between PPARα and PPARδ in UQ10 binding.

Intriguingly, in the mammalian heart, PPARδ differs from PPARα by targeting a different set of downstream genes and promoting myocardial glucose utilization (35). Our structural and mutagenesis analyses raise the possibility that UQ may differentially engage PPAR isoforms and thereby influence distinct metabolic transcriptional programs. Taken together, these results suggest that abnormal intracellular redistribution of UQ in *Coq10a* knockout hearts may trigger aberrant PPAR activation and thereby contribute to the observed cardiac hypertrophy.

### *Coq10a* Knockout Mice Maintain Cardiac Hypertrophy with Preserved Cardiac Function.

We subsequently examined whether the persistent cardiac hypertrophy in *Coq10a^2bp/2bp^* mice was accompanied by impairment of baseline cardiac function. We first analyzed cardiac phenotypes of *Coq10a^2bp/2bp^* mice in adulthood. Echocardiography revealed increased left ventricular wall thickness in *Coq10a^2bp/2bp^* hearts, whereas ejection fraction and fractional shortening were comparable to *Coq10a^+/+^* (*SI Appendix*, Fig. S16 *A* and *B*). Consistently, systolic blood pressure and heart rate did not differ between *Coq10a^+/+^*and *Coq10a^2bp/2bp^* mice (*SI Appendix*, Fig. S16*C*). Preserved cardiac function was maintained for up to 12 mo (*SI Appendix*, Fig. S16*D*). Sirius Red staining of diastole-fixed hearts showed no increase in fibrosis (*SI Appendix*, Fig. S16*E*). We next examined whether the absence of a canonical pathological remodeling program was maintained in adulthood. Although *Col3a1* expression was increased, *Myh7* expression was reduced rather than increased, and the remaining markers examined were unchanged (*SI Appendix*, Fig. S16*F*). Together with preserved cardiac function and the absence of histological fibrosis, these findings do not support activation of a canonical pathological hypertrophic program in adult *Coq10a^2bp/2bp^* hearts. Similarly, *Myh6-Cre;Coq10a^fl/fl^* hearts exhibited increased left ventricular wall thickness with preserved ejection fraction, fractional shortening, and stroke volume (*SI Appendix*, Fig. S17 *A* and *B*). Fibrosis was not increased (*SI Appendix*, Fig. S17*C*), consistent with *Coq10a^2bp/2bp^* mice. Consistently, the oxidative stress marker 4-HNE was unchanged in ventricular lysates from *Myh6-Cre;Coq10a^fl/fl^* hearts compared with *Coq10a^fl/fl^* (*SI Appendix*, Fig. S17*D*). To further assess ventricular function, we performed invasive left ventricular pressure–volume loop analysis in *Myh6-Cre; Coq10a^fl/fl^* mice, which did not reveal overt abnormalities in systolic or diastolic performance compared with *Coq10a^fl/fl^* mice (*SI Appendix*, Fig. S17*E*). We next assessed mitochondrial respiratory activity, because mitochondrial dysfunction can itself promote hypertrophic remodeling. However, oxygen consumption rate measurements in isolated mitochondria and cardiomyocytes revealed no significant differences between adult *Coq10a^+/+^*and *Coq10a^2bp/2bp^* mice (*SI Appendix*, Fig. S18 *A* and *B*). Thus, although *Coq10a* loss alters intracellular UQ partitioning, it is not sufficient to impair mitochondrial respiration in the murine heart. Taken together, *Coq10a*-deficient hearts develop mild cardiac hypertrophy but do not exhibit impaired cardiac function under basal physiological conditions.

### *Coq10a* Knockout Hearts Are Vulnerable to Pressure Overload.

We next asked whether *Coq10a*-deficient hearts are more resistant or sensitive to pathological remodeling. To address this, we subjected adult *Coq10a^2bp/2bp^* mice to TAC and monitored cardiac phenotypes over time (*SI Appendix*, Fig. S19*A*). At 8 wk after TAC, whole-mount analysis showed that *Coq10a^2bp/2bp^* hearts underwent more pronounced cardiac enlargement than *Coq10a^+/+^* hearts (*SI Appendix*, Fig. S19*B*). This was accompanied by a marked increase in the heart weight-to-body weight ratio in the knockout group after TAC (*SI Appendix*, Fig. S19*C*). Serial echocardiography performed at 3, 5, and 8 wk after TAC revealed that cardiac function in *Coq10a^2bp/2bp^* mice declined from 3 wk onward, whereas *Coq10a^+/+^* mice did not show obvious reduction in heart function (*SI Appendix*, Fig. S19*D*). These findings indicate that, although *Coq10a*-deficient hearts maintain near-normal function under basal conditions, loss of *Coq10a* increases susceptibility to pressure overload-induced cardiac remodeling and dysfunction.

## Discussion

Our study identifies Coq10a as a postnatally induced, striated muscle-enriched mitochondrial protein that restrains cardiac growth during a defined developmental window. Although yeast Coq10p has long been implicated in UQ handling, the physiological role of its mammalian homolog has remained unclear. Here, loss-of-function analyses in mice show that Coq10a limits the magnitude of postnatal cardiac hypertrophic growth. This conclusion is supported by two independent germline knockout lines and by a cardiomyocyte-specific conditional model, indicating that Coq10a acts, at least in large part, within cardiomyocytes in the postnatal heart. The severity of the phenotype varied across alleles or background, with the *Coq10a^ORF^* line showing growth retardation, but the shared cardiac phenotype across models argues that enhanced postnatal hypertrophic growth is a consistent consequence of *Coq10a* deficiency.

This phenotype differs from the severe cardiomyopathy commonly associated with primary defects in the UQ biosynthetic pathway. Pathogenic variants in several UQ biosynthetic genes, including *COQ2*, *COQ4*, *COQ7*, *COQ8B*, and *COQ9*, have been linked to infantile or neonatal cardiomyopathy in humans. By contrast, *Coq10a* loss did not produce overt fibrosis, increased apoptosis, or baseline systolic dysfunction, and the hypertrophic response arose within the first 2 wk after birth, the same period during which the mammalian heart normally undergoes physiologic postnatal growth. Deletion of *Coq10a* in the adult did not lead to the hypertrophic phenotype. Together, these findings place Coq10a closer to a regulator of developmental cardiac growth than to a core determinant of basal cardiac performance.

A central mechanistic conclusion of this study is that Coq10a regulates intracellular UQ partitioning. In *Coq10a*-deficient hearts, UQ was redistributed toward the microsomal fraction without a significant increase in the mitochondrial fraction. Because the microsomal fraction contains multiple nonmitochondrial membrane compartments, this fractionation approach does not identify the specific organelle in which UQ accumulates. Future studies using organelle-resolved lipidomic or targeted imaging approaches will be required to determine the precise site of UQ redistribution. Recent studies have highlighted extramitochondrial roles of UQ, including functions at the plasma membrane in suppressing ferroptosis, supporting the idea that subcellular localization of UQ is functionally important (36, 37). In this respect, the mouse data are consistent with the proposed lipid-chaperone function of yeast Coq10p (9). At the same time, our results also reveal an important species difference. In *Saccharomyces cerevisiae*, *coq10* mutants retain near-normal mitochondrial UQ levels yet exhibit a clear respiratory defect, whereas in the mouse heart, altered UQ partitioning and UQ redox imbalance were not accompanied by impaired baseline mitochondrial respiration. One possible explanation is that, in the murine heart, the increase in total UQ is sufficient to preserve a functionally competent respiratory UQ pool despite abnormal intracellular distribution. Thus, although Coq10a is required for proper intracellular UQ handling, its loss alone is not sufficient to compromise mitochondrial respiration in the mammalian heart, at least under physiological conditions.

The basis of this buffering remains unresolved. *Coq10b* expression was unchanged, whereas *Coq3* and *Coq6* mRNA levels increased in *Coq10a^2bp/2bp^* hearts, consistent with a compensatory response within the UQ biosynthetic machinery. A prior study showed that PPARα-dependent signaling can increase UQ biosynthesis in vivo (38), although that work was performed in a different tissue and does not directly explain the present cardiac phenotype. More broadly, major questions remain about how UQ is distributed, retained, and turned over within mammalian cells. For that reason, the mechanism underlying the increase in total UQ in *Coq10a*-deficient hearts cannot yet be assigned to enhanced synthesis alone. Additional work will be needed to distinguish altered synthesis from altered trafficking, retention, or clearance.

Our findings further suggest that the main consequence of *Coq10a* loss in the postnatal heart is not a failure of oxidative phosphorylation at baseline but altered UQ signaling. Microsomal UQ increased selectively in *Coq10a*-deficient hearts. This observation is mechanistically plausible because, although PPARα is classically viewed as a nuclear receptor, PPARα and PPARγ can undergo dynamic nucleo-cytoplasmic shuttling (39), and in the heart, PPARα has been reported to show increased cytoplasmic localization in settings of MEK1 activation, including voluntary exercise (40). PPAR family members are also widely recognized as lipid-sensing transcription factors whose activity is modulated by endogenous and exogenous lipid ligands, and UQ10/idebenone have been reported to bind and activate PPARα/γ (8). In the heart, PPAR family members regulate programs of fatty acid and glucose metabolism (18), and our pharmacologic data indicate combined inhibition of PPARα and PPARγ, but not inhibition of PPARδ, attenuates the hypertrophic phenotype caused by *Coq10a* loss. Because the PPARα and PPARγ inhibitors were coadministered, these experiments do not establish whether PPARα alone is necessary or sufficient for the hypertrophic phenotype. The effects of PPARα activation on cardiac growth appear to be context dependent. Systemic treatment with gemfibrozil, a PPARα agonist, attenuated pressure overload-induced hypertrophy in adult rats (41), whereas cardiomyocyte-restricted PPARα overexpression caused cardiac hypertrophy and dysfunction in mice (42). Differences in developmental stage, disease context, mode and magnitude of receptor activation, and systemic vs. cardiomyocyte-autonomous effects may account for these divergent outcomes. Together with the computational and mutagenesis analyses, these findings support a model in which altered UQ partitioning may influence PPARα/γ-associated signaling in the postnatal heart. However, we did not directly measure binding of endogenous UQ to PPAR proteins or PPAR chromatin occupancy in cardiac tissue. The methionine residues identified in PPARα contributed to UQ-responsive reporter activity in HEK293T cells, but they were not sufficient to confer UQ responsiveness on PPARδ and therefore do not fully account for PPAR isoform selectivity. Because the present study did not test UQ10 or idebenone supplementation in vivo, the potential therapeutic implications of ubiquinone-related interventions remain speculative.

An additional implication of this work is that Coq10a appears to be more important for cardiac resilience than for baseline function. Under physiological conditions, *Coq10a*-deficient hearts showed mild hypertrophy with preserved systolic function and mitochondrial respiration. Under pressure overload, however, the phenotype became markedly worse, with exaggerated cardiac enlargement and reduced function after TAC. These findings suggest that Coq10a is not required for the maintenance of resting cardiac performance, but becomes important when the heart is subjected to stress. Why *Coq10a*-deficient hearts are vulnerable to pressure overload remains unknown. Recent work has implicated reduced ubiquinone in reverse electron transport and oxidative stress under pathological conditions (43), raising the possibility that altered UQ redox state or distribution contributes to this stress sensitivity. Alternatively, increased UQ abundance in *Coq10a*-deficient hearts fails to confer benefit under TAC because PPARα signaling is itself suppressed during pressure-overload remodeling, including through reduced expression and impaired nuclear activity (40, 44). In this setting, excess UQ may no longer be efficiently translated into receptor-dependent adaptive signaling. It will therefore be important to determine whether this stress sensitivity reflects limited metabolic reserve, including impaired capacity to meet increased energetic demand, abnormal UQ signaling, defective adaptive remodeling, or a combination of these mechanisms.

Although *COQ10A* has not been established as a Mendelian disease gene, its altered expression in human cardiac disease datasets raises the possibility that reduced COQ10A activity may modify susceptibility to cardiac stress (45, 46). Future studies should determine whether rare or regulatory CoQ10A variants contribute to unexplained cardiac remodeling. Beyond Coq10a, our analysis highlights a broader set of tissue-enriched, postnatally regulated mitochondrial proteins whose physiological functions remain largely unexplored, providing a resource for future studies of organ-specific mitochondrial biology.

## Materials and Methods

Detailed methods are provided in *SI Appendix*, including animals, western blot, neonatal mouse cardiomyocyte culture and treatment, RT-qPCR analysis, adult mouse cardiomyocyte isolation for cell size analysis, protein sequence alignment, histology, quantification of total cardiomyocyte number, AAV injection, RNA-seq analysis, measurement of cardiac function, pressure–volume loop analysis, subcellular fractionation of heart tissue, measurement of mitochondrial respiration, measurement of ubiquinone levels, *Coq10a* overexpression and artificial UQ localization analysis, luciferase reporter assay, mixed-solvent molecular dynamics simulation, molecular docking, minimally invasive TAC surgery, and statistical analysis.

## Supplementary Material

Appendix 01 (PDF)

Dataset S01 (XLSX)

Dataset S02 (XLSX)

Dataset S03 (XLSX)

Dataset S04 (XLSX)

## Data Availability

Bulk RNA-seq data generated from *Coq10a^2bp/2bp^*, *Myh6-Cre;Coq10a^fl/fl^,* and control mice have been deposited in the Gene Expression Omnibus (GEO) under accession number GSE342996 (47). All other data are included in the manuscript and/or supporting information.
