# Supplementary material for: A muscle-enriched mitochondrial protein regulates postnatal heart growth through mitonuclear ubiquinone–PPAR signaling
Source: Proc Natl Acad Sci U S A. 2026 Oct 16;123(42):e2619615123. doi: 10.1073/pnas.2619615123 (PMC13684178; doi:10.1073/pnas.2619615123)
Supplement: Supplementary file 1 [file pnas.2619615123.sapp.pdf]

PNAS

Supporting Information for

A muscle-enriched mitochondrial protein regulates postnatal heart growth through mitonuclear ubiquinone-PPAR signaling

Kentaro Hirose[1,2,3,4,5,17,19*], Xiaoxin Chen[1,2,3,4,19], Chakrapani Kalyanaraman[6,18], Mark T. Waddingham[5], Makoto Nakamura[1,2,3,4], Feng Zhang[1,2,3,4], Christina Chen[1,2,3,4], Shun Yao[1,2,3,4], Steven Chang[1,2,3,4], Hongyao Yu[7], Jiajia Wang[7], Emanuele Barca[8], Haruko Nakano[9], Austin Hsu[10], Sheamin Khyeam[1,2,3,4], Sara Gonzalez Ortega[1,2,3,4], Kazuki Tajima[3,11], Takeshi Yoneshiro[3,11], Hao Zhang[1,12], Arun Padmanabhan[1,12], Shingo Kajimura[3,11,13], Saptarsi Haldar[10,12], Atsushi Nakano[9], Masatoshi Murai[14], Yoshitaka Fujihara[15], Kinya Otsu[16], Catarina M. Quinzii[8], Matthew P. Jacobson[6], Guang Hu[7], James T Pearson[5], Guo N. Huang[1,2,3,4 *]

[1]Cardiovascular Research Institute, University of California, San Francisco, San Francisco, CA, 94158, USA.
[2]Department of Physiology, University of California, San Francisco, San Francisco, CA, 94158, USA.
[3]Eli and Edythe Broad Center for Regeneration Medicine and Stem Cell Research & Bakar Aging Research Institute, University of California, San Francisco, San Francisco, CA, 94158, USA.
[4]Bakar Aging Research Institute, University of California, San Francisco, San Francisco, CA, 94158, USA.
[5]Department of Cardiac Physiology, National Cerebral and Cardiovascular Center Research Institute, Suita, Osaka, 564-8565, Japan.
[6]Department of Pharmaceutical Chemistry, University of California, San Francisco, CA, 94158, USA.
[7]Epigenetics and Stem Cell Biology Laboratory, National Institute of Environmental Health Sciences, RTP, NC 27709, USA.
[8]Department of Neurology, Columbia University Medical Center, New York, NY, USA.
[9]Department of Molecular, Cell, and Developmental Biology, University of California, Los Angeles, Los Angeles, USA.
[10]Gladstone Institutes, San Francisco, CA, USA.
[11]UCSF Diabetes Center, San Francisco, CA, USA.
[12]Department of Medicine, Division of Cardiology, University of California, San Francisco, San Francisco, CA, USA.
[13]Department of Medicine, Beth Israel Deaconess Medical Center, Boston, MA, 02215, USA.
[14]Division of Applied Life Sciences, Graduate School of Agriculture, Kyoto University, Kyoto, Japan.
[15]Department of Advanced Medical Technologies, National Cerebral and Cardiovascular Center, Suita, Osaka, Japan.
[16]Office of the President, National Cerebral and Cardiovascular Center, Kishibe-Shimmachi, Suita, Osaka, Japan.
[17]Cambridge Stem Cell Institute, University of Cambridge, Cambridge, UK.
[18]Nine Square Therapeutics, Inc., 2 Tower Place, South San Francisco, CA 94080.
[19]Equal contribution

* Corresponding authors: Kentaro Hirose and Guo N. Huang
Email: kh817@cam.ac.uk (K. H.) and Guo.Huang@ucsf.edu (G. N. H.)

This PDF file includes:

- Supplementary Materials and Methods
- Supplementary References
- Supplementary Figures S1 to S19
- Legends for Dataset S1 to S4

Other supporting materials for this manuscript include the following:

- Dataset S1 to S4

Supplementary Materials and Methods

Animals

All animal procedures were performed in accordance with institutional guidelines and were approved by the relevant Institutional Animal Care and Use Committees. Experiments using the *Coq10a*^{2bp} knockout line, CD-1 mice, *Myh6-merCremer* mice, and *Myh6-Cre;Thra*$^{DN/+}$ mice were conducted at the University of California, San Francisco, approved by the Institutional Animal Care and Use Committee (IACUC) of the University of California, San Francisco, and performed in accordance with UCSF institutional guidelines. Experiments involving the *Coq10a*$^{ORFdel/ORFdel}$ and *Coq10a*$^{fl/fl}$ mouse lines were conducted at the National Cerebral and Cardiovascular Center, approved by the Institutional Animal Care and Use Committee of the National Cerebral and Cardiovascular Center, and performed in accordance with institutional guidelines for animal experiments.

CD-1 mice were obtained from Charles River. *Coq10a*^{2bp} knockout mice were generated at the UCSF CVRI Mouse Core Facility by injecting purified sgRNA targeting *Coq10a*, 5′-TTACTCGGAGCGTAGGATCA-3′, together with Cas9 protein into CD-1 fertilized oocytes. *Coq10a*^{2bp} knockout mice were maintained according to UCSF institutional guidelines. For genotyping of the *Coq10a*^{2bp} allele, PCR was performed using a forward primer, 5′-AGAATCGTGACGCTGCCTCTC-3′, and a reverse primer, 5′-ACAGGCTGTACTACCTAGTC-3′. The generation of *Myh6-merCremer* and *Nkx2.5*$^{f/f}$ mice has been described previously (1, 2). To induce recombination of floxed alleles, 1 mg tamoxifen dissolved in peanut oil was given to adult mice via intraperitoneal injection per day for four days. Generation of *Myh6-Cre;Thra*$^{DN/+}$ mice, RNA-seq analysis in *Myh6-Cre;Thra*$^{DN/+}$ mice and ChIP-seq analysis against *Thra-GFP* were described in our previous paper (3).

Coq10a$^{ORFdel/ORFdel}$ and cardiomyocyte-specific conditional knockout (*Myh6-Cre; Coq10a*$^{fl/fl}$) lines were generated at the National Cerebral and Cardiovascular Center. Specifically, *Coq10a*$^{ORFdel/ORFdel}$ and *Coq10a*$^{fl/fl}$ mouse lines were produced by CRISPR/Cas9-mediated genome editing in B6D2 fertilized eggs (Japan SLC, Shizuoka, Japan) and were subsequently backcrossed onto the C57BL/6N background. For the *Coq10a*$^{ORFdel/ORFdel}$ allele, sgRNAs were designed to disrupt the Coq10a coding region from the translation initiation region to the terminal coding region, using 5′-GCTCCCGGCCCCGACGCGCG-3′ and 5′-ACAATCCCTCGTCTCAAGTT-3′. F0

founders were identified by PCR-based genotyping and Sanger sequencing, and were intercrossed to establish stable lines. For genotyping of the *Coq10a*^{ORFdel} allele, PCR was performed using separate primer sets to detect the wild-type and knockout alleles. The wild-type allele was detected using a forward primer, 5′-CTACATCTCCCGACAGGCAGCG-3′, and a reverse primer, 5′-CGCGTATCTCCAGAACTCCATTCGAAC-3′. The knockout allele was detected using a forward primer, 5′-CTACATCTCCCGACAGGCAGCG-3′ and a reverse primer 5′-CACAGAGACGGTGACAGCCCTGG-3′. Heterozygous mice showed both wild-type and knockout allele bands, whereas homozygous *Coq10a*$^{ORFdel/ORFdel}$ mice showed only the knockout allele band.

To generate a cardiomyocyte-specific Coq10a knockout, a *Coq10a*$^{fl/fl}$ allele was engineered by CRISPR/Cas9-mediated homology-directed repair to enable Cre-dependent excision of exon 3. sgRNAs used for floxing were 5′-GTCTGTGTTCCTTGGTTAAT-3′ and 5′-GTAGTCTTTGACCGTGAAAC-3′, together with an ssDNA megamer donor containing loxP sites. For genotyping of the *Coq10a*^{fl} allele, PCR was performed using a forward primer, 5′-GCCATCTGGGTTTGTCTGTGTTCC-3′, and a reverse primer, 5′-TCCTAGGTTGGCCTAGCATGACTC-3′. *SpeI* digestion of the PCR product was used to detect the loxP-containing floxed allele. *Myh6-Cre;Coq10a*$^{fl/fl}$ mice were generated by crossing *Coq10a*$^{fl/fl}$ mice with the *Myh6-Cre* driver line, which was kindly provided by Dr Otsu (4).

Adult mice were 2–8 months old. For all other experiments, both male and female mice were used, and no sex-dependent difference was observed.

For PPAR inhibitor study *in vivo*, wild type and *Coq10a* knockout animals were injected with inhibitors at the dose of 0.5 mg/kg body weight daily from day 0 to day 13. PPARα inhibitor GW6471 (Sigma Cat#G5045) and PPARγ inhibitor T0070907 (Sigma Cat#T8703) were dissolved in 10% PEG400, 10% Tween80, 10% DMSO and 70% saline. PPARδ inhibitor GSK3787 (Sigma Cat#G7423) was dissolved in 90% peanut oil and 10% DMSO.

Western blot

Proteins were lysed in 1% SDS (reconstituted in PBS) supplemented with proteinase inhibitor (Fisher, PI88666) from frozen mouse heart ventricles. Protein concentration was measured using the BCA Protein Assay Kit (Pierce). 100 µg protein lysate was first loaded

to a NuPAGE 4-12% Bis-Tris Gel (Invitrogen) for separation, and then transferred to a PVDF membrane (BIO-RAD). Protein loading was assessed using Ponceau S staining. The blot was visualized using ECL Western Blotting substrate (Pierce). The following primary antibodies were used: rabbit anti-Coq10a (Proteintech, 17812-1-AP, 1:1000), mouse anti-Troponin T (Thermo Fisher Scientific, MS295P1, 1:1000), mouse anti-Tom20 (Santa Cruz Biotechnology, sc-17764, 1:1000), rabbit anti-4-HNE (1:1000), mouse anti-glutathione (Santa Cruz Biotechnology, clone D8, sc-52399), rabbit anti-ATP5F1 (Proteintech, 15999-1-AP, 1:2500), rabbit anti-PDI (Proteintech, 11245-1-AP, 1:2500), rabbit anti-N-cadherin (Proteintech, 22018-1-AP, 1:2500), rabbit anti-Syntaxin 6 (Proteintech, 10841-1-AP, 1:2500), rabbit anti-Catalase (Proteintech, 21260-1-AP, 1:2500), rabbit anti-LIMPII (Proteintech, 27102-1-AP, 1:2500), rabbit anti-Histone H3 (Cell Signaling Technology, 9715, 1:2500), Phospho-4EBP1(Thr37/46) (Cell signaling Technology, Cat#2855), 4EBP1 (Cell signaling Technology, Cat#9452), Phospho-p70 S6 Kinase (Thr389) (Cell signaling Technology, Cat#9234S), p70 S6 Kinase (Cell signaling Technology, Cat#34475), Phospho-S6 (Ser240/244) (Cell signaling Technology, Cat#2215), and S6 (Cell signaling Technology, Cat#2217).

Neonatal mouse cardiomyocyte culture and treatment

Mice were anesthetized on ice, and then the freshly isolated heart was cannulated onto a needle and perfused with 2 mg/mL collagenase II (Worthington) until the heart lost integrity. The isolated cardiomyocytes were plated on a gelatin-coated 96-well plate in 10% FBS in DMEM supplemented with 1 μM Ara-C and 100 μg/mL primocin. At 24 hours, the media was replaced with 0.05% BSA in DMEM supplemented with 100 μg/ml primocin, and 30 nM or 300 nM T3 were added. The cells were treated for another 72 h before harvesting for RT-qPCR analysis.

For knockdown of *Tbx5* and *Nkx2.5*, CMs were plated on a 96-well plate in DMEM medium with 10% FBS. At 24 h after plating, 1 pmol Nkx2.5 siRNA (Sigma, siRNA ID: SASI_Mm01_00138958) and Tbx5 siRNA (Sigma, siRNA ID: SASI_Mm01_00069158) were transfected into the cells using Lipofectamine RNAi MAX Transfection Reagent (Thermo Fisher Scientific) in accordance with the manufacturer's instructions. At 24 hours after transfection, the medium was replaced with DMEM containing 0.05% BSA with/without T3 (300 nM). The cells were further maintained for another 48 h before harvesting.

RT-qPCR analysis

At the end point, total RNA was isolated using TRIzol, and then cDNA was synthesized using iScript™ cDNA Synthesis Kit. qPCR was performed using the SYBR Select Master Mix (Applied Biosystems, 4472908) and the 7900HT Fast Real-Time PCR system or QuantStudio 5 Real-Time PCR system (Applied Biosystems). Fold changes in transcript levels were determined using the $_{\Delta\Delta}$Ct method. The following primers were used.

Primer name	Sequence (5' to 3')
Gapdh forward	AAG GTC ATC CCA GAG CTG AA
Gapdh *reverse*	CTG CTT CAC CAC CTT CTT GA
Tbx5 forward	ATGGCCGATACAGATGAGGG
Tbx5 reverse	TTCGTGGAACTTCAGCCACAG
Nkx2.5 forward	GACAAAGCCGAGACGGATGG
Nkx2.5 reverse	CTGTCGCTTGCACTTGTAGC
Nppa forward	GCTTCCAGGCCATATTGGAG
Nppa reverse	GGGGGCATGACCTCATCTT
Nppb forward	GAGGTCACTCCTATCCTCTGG
Nppb reverse	GCCATTTCCTCCGACTTTTCTC
Acta1 forward	CCCAAAGCTAACCGGGAGAAG
Acta1 reverse	CCAGAATCCAACACGATGCC
Myh7 forward	ACTGTCAACACTAAGAGGGTCA
Myh7 reverse	TTGGATGATTTGATCTTCCAGGG
Col1a1 forward	GCTCCTCTTAGGGGCCACT
Col1a1 reverse	CCACGTCTCACCATTGGGG
Col3a1 forward	CTGTAACATGGAAACTGGGGAAA
Col3a1 reverse	CCATAGCTGAACTGAAAACCACC
Myh6 forward	GCCCAGTACCTCCGAAAGTC
Myh6 reverse	GCCTTAACATACTCCTCCTTGTC
Pdk4 forward	AGGGAGGTCGAGCTGTTCTC
Pdk4 reverse	GGAGTGTTCACTAAGCGGTCA
Cpt1b forward	GCACACCAGGCAGTAGCTTT
Cpt1b reverse	CAGGAGTTGATTCCAGACAGGTA
Cd36 forward	ATGGGCTGTGATCGGAACTG
Cd36 reverse	GTCTTCCCAATAAGCATGTCTCC
Acox1 forward	TAACTTCCTCACTCGAAGCCA
Acox1 reverse	AGTTCCATGACCCATCTCTGTC
Coq10a forward	GAGTTCGTGCCGTGGTGTAA
Coq10a reverse	TGGAGACGGCAGAGGTGTAA
Actb forward	AGTGTGACGTTGACATCCGT
Actb reverse	TGCTAGGAGCCAGAGCAGTA

Adult mouse cardiomyocyte isolation for cell size analysis

Mice were injected with 6.67 mg/kg bodyweight of heparin (1000 IU/mL) intraperitoneally 30 minutes before anesthetizing the mice with 20% ethyl-carbamate. The hearts were rapidly excised, and the aorta was cannulated onto a Langendorff apparatus and perfused

with 2 mg/mL collagenase II (Worthington). Digestion was stopped after the hearts lost partial integrity. Hearts were then unmounted from the Langendorff apparatus, ventricles isolated, and gently triturated with forceps to further release cardiomyocytes. Isolated cardiomyocytes were then fixed in 2% PFA for 15 minutes at room temperature, spotted onto glass slides, and stained with DAPI to visualize nuclei. Cardiomyocyte size and nucleation were analyzed using ImageJ. At least 200 cells were used for the quantifications.

Protein sequence alignment

Protein sequences were obtained from NCBI. The following shows the protein accession numbers of the sequences in various species. These sequences were selected based on similarity to mouse *Coq10a* using BLAST. *S. cerevisiae* (NP_014635.1), *E. coli* (WP_137445723.1), *S. pombe* (NP_587994.1), *C. elegans* (NP_001040866.1), *D. melanogaster* (NP_724484.1), *D. rerio* (XP_009295305.1), *X. laevis* (XP_018105495.1), *P. muralis* (XP_028565875.1), *M. domestica* (XP_001379337.1), *M. musculus* (NP_001074509.1), Rattus norvegicus (NP_001102197.1), and *H. sapiens* (NP_653177.3). The sequence alignment was generated using Clustal Omega.

Histology

3.7% paraformaldehyde (PFA) fixed hearts were embedded in paraffin, and then sectioned at 5 μm thickness. For fibrosis staining, sections were soaked in Sirius Red for 25 min at room temperature. For eosin and hematoxylin staining, sections were stained with Eosin for 3 min and hematoxylin for 5 min at room temperature. Sections were imaged using Axio Scan.Z1 or NanoZoomer and fibrotic area was quantified using ImageJ.

Immunohistochemical staining

Methods for sectioning and immunostaining were described in our previous paper (3). Briefly, freshly dissected heart was embedded in O.C.T. Compound and flash frozen on metal box in liquid nitrogen. Embedded samples were then sectioned with a Leica CM3050S to 5 μm thickness. PFA-fixed sections were permeabilized in 0.2% Triton X-100 in PBS, blocked in 5% normal donkey serum (NDS) in PBST, and incubated with primary antibodies. The following primary antibodies were used: rat anti-Ki67 eFluor 570 (eBiosciences, 41-5698-80, 1:200), rabbit anti-PCM1 antibody (Sigma, HPA023370, 1:2000) and mouse anti-Troponin T (Thermo Fisher Scientific, MS295P1, 1:200) in PBST

overnight at 4°C. After washing with PBST, samples were incubated in secondary antibodies with fluorescence (1:500) for 2 hours at room temperature. Cell membrane was labeled with wheat germ agglutinin (1:200) and nuclei were stained with DAPI. TUNEL staining was performed according to the manufacturer's instructions.

Quantification of total cardiomyocyte number

Hearts were fixed in 3.7% formaldehyde for 1 hour. After removal of the atria, the ventricle was then cut into four pieces and digested in PBS containing 4 mg/mL collagenase II (Worthington) with gentle shaking at 37°C until complete digestion. The digestion buffer was changed daily. All dissociated cardiomyocytes were pooled and counted using a hemocytometer.

AAV injection

P1 and adult *Coq10a*$^{fl/fl}$ mice were injected with AAV9-Tnnt2-Cre-EGFP or AAV9-Tnnt2-nlsEGFP virus at a dose of 5 x 10^{11} and 3 x 10^{12} genomic copy per mouse via retro-orbital and subcutaneous route, respectively, using a 31G insulin needle. Hearts were harvested two-weeks after injection for analysis.

RNA-seq analysis

For *Coq10a*$^{2bp/2bp}$ hearts, RNA was isolated from heart samples using TRIzol Reagent (Thermo Fisher Scientific) according to the manufacturer's instructions and further pre-cleaned by Zymo RNA Clean & Concentrator 5. 1 µg of total RNA was used for library preparation with Illumina Tru-seq RNA Library Prep Kit V2. NEXTflex Unique Dual Index Barcodes (PerkinElmer) were used to minimize index hopping. Samples were normalized, pooled and sequenced on Illumina NovaSeq 6000 to obtain 50 bp paired-end reads.

For *Myh6-Cre;Coq10a*$^{fl/fl}$ hearts, total RNA was isolated from frozen P14 heart tissue using the NucleoSpin RNA kit (Macherey-Nagel) according to the manufacturer's instructions. RNA quality was assessed using TapeStation or BioAnalyzer. Libraries were prepared using the SMART-Seq v4 Ultra Low Input RNA kit, and sequencing was performed on the NovaSeq system to obtain 150-base paired-end reads. Gene- and transcript-level expression values were calculated as read counts and TPM values using the default parameters of the expression analysis pipeline.

Measurement of cardiac function

Left ventricular systolic function was evaluated by two-dimensional echocardiography with Visual Sonics Vevo 3100 equipped with a 40 MHz probe. Animals were anesthetized with 0.5-1.0% isoflurane and hair was removed over the measurement area. The mice were then placed in a supine position on a heating pad. To measure ejection fraction and fractional shortening, short axis images were acquired at the level of the papillary muscle with M-mode, and the left ventricle internal dimensions (LVIDs) were determined in both diastole and systole.

Blood pressure and heart rate were measured using BP-2000 Blood Pressure Analysis System™ (Visitec Systems). The values were determined by averaging measurements obtained over three days.

Pressure-volume loop analysis

Mice were anesthetized with isoflurane (3% for induction; 1.5% for maintenance) in oxygen-enriched air (~50% O_2) and once deep surgical anesthesia was confirmed by the absence of the pedal reflex, underwent tracheal intubation and then mechanically ventilated under pressure control (tracheal pressure ~12-15 cm H_2O, ~200 breaths/min; PhysioSuite®, Kent Scientific, Torrington, CT, USA). Body temperature was maintained at 37 ±1°C throughout the experiment with a rectal thermistor connected to a heating pad. Catheterization of the LV was performed via an open-chest approach, with a thoracotomy performed to fully expose the heart. A 1.4F miniaturized pressure-volume (PV) catheter (SPR-839, Millar Inc., TX, USA) was positioned parallel to the LV long-axis by the apical puncture approach (5). Mice were allowed to stabilize for ~10-15 min prior to PV loop data acquisition commencing. PV loops were recorded under steady-state conditions with ventilation suspended at end inspiration to avoid artefacts from catheter movement due to breathing. PV loop data was acquired with an MPVS-300 system (Millar Inc., TX, USA) connected to a PowerLab 16/30 (ADInstruments, NSW, Australia) and recorded in LabChart Pro software (v. 8.1.30, ADInstruments) at a sampling rate of 1,000 Hz. The volume signal of the PV catheter was calibrated against echocardiography-derived volumes for each mouse. PV loop data was analyzed off-line with the use of the PV Loop Module (v2.6, ADInstruments) within the LabChart Pro software.

Subcellular fractionation of heart tissue

Ventricular tissue was fractionated by differential centrifugation using the Mitochondria Isolation Kit for Tissue (Abcam, ab110168). Fresh hearts were excised, atria were

removed, and ventricles were washed, minced, and homogenized in ice-cold isolation buffer. Homogenates were centrifuged at 600 × g to pellet nuclei and debris (nuclear fraction). The post-nuclear supernatant was centrifuged at 12,000 × g to pellet mitochondria (mitochondrial fraction), and the resulting supernatant was centrifuged at 100,000 × g to collect the microsomal pellet. Fractions for immunoblotting were lysed in 1% SDS supplemented with protease inhibitor cocktail (Roche, 5892970001), and mitochondrial pellets were additionally washed in isolation buffer supplemented with inhibitors (Abcam, ab201111) prior to downstream assays. Protein concentration was determined by BCA assay.

Measurement of mitochondrial respiration

Mitochondrial respiration was measured using a Seahorse Bioscience XFe24 Extracellular Flux Analyzer. 5 µg of mouse heart mitochondria was added in each well of Seahorse XF24 24-well microplate, and 450 µL of 1X MAS containing 4% BSA, 10 mM pyruvate and 5 mM malate was added. Isolated mitochondria were treated with 2 µM rotenone, 10 mM succinate, 5 µM antimycin A and 100 µM N,N,N',N'-Tetramethyl-p-phenylenediamine (TMPD) with 10 mM ascorbate (Asc) at the indicated time points.

Adult cardiomyocytes were isolated from wild-type and Coq10a knockout mice and plated onto laminin-coated XFe96/XF Pro cell culture microplates (300 cells per well). Cells were incubated for 1 hour prior to measurement in sodium bicarbonate-free DMEM supplemented with 4 mM glutamine, 100 U/mL penicillin, 100 µg/ml streptomycin, 1 mM sodium pyruvate, and 1% insulin–transferrin–selenium (ITS). Oxygen consumption rate (OCR) was measured using an XFe96/XF Pro FluxPak under basal conditions and following sequential injections of 1 µM oligomycin, 0.5 µM FCCP, and 1 µM antimycin A + 1 µM rotenone. Five technical replicates per mouse were averaged, and each mouse was treated as one biological replicate (n = 4 per genotype).

Measurement of ubiquinone levels

For *Coq10a*$^{2bp/2bp}$ hearts, UQ9 and UQ10 were extracted from ventricular tissue of Coq10a knockout mice and littermate controls as described previously (6). Lipid extracts were separated by high-performance liquid chromatography (HPLC), and UQ concentrations were determined by comparison of peak areas with standard solutions of known concentration.

For *Myh6-Cre;Coq10a*$^{fl/fl}$ hearts, UQ species were quantified from ventricular homogenates, subcellular fractions, and isolated mitochondria. UQ species were extracted using a two-phase partition method with acidified methanol and hexane, dried under vacuum, and reconstituted in 2-propanol before HPLC analysis. UQ species were separated on a reversed-phase C18 column and detected by electrochemical detection at +600 mV. Oxidized and reduced UQ9 and UQ10 standards were used for retention time matching, and quantification was performed by external calibration using peak areas.

Coq10a overexpression and artificial UQ localization analysis

H9C2 cells were maintained in high-glucose DMEM supplemented with 10% fetal bovine serum and penicillin–streptomycin. Two lentiviral constructs were used: a mouse Coq10a overexpression vector encoding mCoq10a (NM_001081040.1) and a rat Coq10a knockdown vector expressing an shRNA targeting rCoq10a (target sequence: 5′-ACTACTCAGTTGATGAAATTT-3′). Plasmid construction and lentivirus production were outsourced to VectorBuilder. Cells were transduced at ~40–50% confluence (MOI 10) in the presence of polybrene (5 μg/mL). Medium was replaced the next day, and puromycin selection was initiated 72 h post-transduction. Knockdown and overexpression were verified at day 5 post-transduction by RT–qPCR using rCoq10a primers (*rCoq10a*-2F, 5′-TTCCCGCCTACCCTCGAACCTG-3′; *rCoq10a*-2R, 5′-GCGCTCAAAGGCAGCAACGTTC-3′) and *Coq10b* primers (*Coq10b*-F, 5′-CAGCTCGGACCAGTCAGAG-3′; *Coq10b*-R, 5′-TCCTCCATTCCTGATACTACGTC-3′). For artificial ubiquinone localization, cells were incubated with 10 μM UQ-3-yne, an artificial ubiquinone probe possessing a clickable alkyne tag that was synthesized as described previously (7), for 24 h. Cells were then fixed with 4% paraformaldehyde, and processed for copper-catalyzed azide-alkyne cycloaddition using the Click-iT Protein Reaction Buffer Kit (Thermo Fisher Scientific, C10276).

Mixed-solvent molecular dynamics simulation (MSMD)

For structural comparison of the PPAR ligand-binding pockets, PPARγ (Protein Data Bank (PDB) ID: 5U5L) and PPARδ (PDB ID: 2GWX) structures were aligned to the oleic acid-bound PPARα structure (PDB ID: 6LX8). Residues surrounding the fatty acid-binding pocket and hydrophobic tunnel were compared among the three PPAR isoforms.

For mixed-solvent molecular dynamics simulations, PPARα, PPARγ, and PPARδ structures (PDB IDs: 3VI8, 5U5L, and 2GWX, respectively)

were obtained from PDB. These structures were subjected to a protein preparation step (Schrodinger Inc). Prior to this step we removed water molecules, ions and small molecules that were not bound in the fatty acid binding pocket. During the protein preparation step, any missing side chains were built in, hydrogen atoms were added to the protein and the ligand, protonation states of titratable residues were adjusted, side chain orientation of Asn, Thr and Tyr residues were optimized, and finally the protein structure was energy minimized such that heavy atoms did not move beyond 0.3 Å from their starting positions. Following the protein preparation step, ligands were discarded, and the protein structures were subjected to MSMD simulation. We used isoprenol as a co-solvent in the MSMD simulation. Parameter files for isoprenol were prepared using Amber suite's Antechamber. We then used Packmol to randomly place co-solvent molecules surrounding the protein in a 10 x 10 x 10 $Å^3$ box. Protein and co-solvent mixture were placed in 100 x 100 x 100 $Å^3$ box and filled with TIP3P water molecules and counter ions to neutralize the charge. We calculated the number of co-solvent molecules to be added so that the final system would have a 1% co-solvent concentration relative to water. Once the system was prepared, we subjected it to energy minimization (2000 steps conjugate gradients and 2000 steps steepest descent), 20 ps restrained heating, 20 ps restrained pressure equilibration and 1 ns unrestrained equilibration. Subsequently, we ran 100 ns NVT molecular dynamics (MD) simulation using AMBER MD simulation package. MD simulation trajectories were saved every picosecond (ps). Co-solvent densities were calculated from these trajectories using MDAnalysis Python package.

Molecular docking

To understand how idebenone and UQ10 bind to PPAR-alpha and gamma and not bind to delta, we wanted to understand their binding mode against these proteins. We examined all available structures of these proteins to see if any of them were co-crystallized with a ligand that had p-benzoquinone moiety that was present in idebenone and UQ10. Unfortunately, none of the ligands in the available structures had the p-benzoquinone group. Therefore, we decided to find out hotspots for p-benzoquinone binding. In recent years, mixed-solvent molecular dynamics simulations followed by evaluation of co-solvent density is emerging as a robust method to identify potential ligand binding hotspots in a protein (8). For each of the proteins we chose a protein structure, removed any ligand, metal ion and water present in the structure and ran 100 ns mixed-

solvent molecular dynamics simulations using AMBER. PDB IDs of PPARα, γ and δ proteins chosen in our calculations are respectively 2p54, 4ema and 1gwx. Prior to running molecular dynamics simulations, we subjected the protein structures to a protein-preparation step using Protein-Preparation Wizard (Schrodinger Inc). Missing side chains and loops were built and optimized during this step. The resulting structure was minimized such that the heavy atoms did not move beyond 0.3 Å. We used 5% p-benzoquinone and 95% water in our simulations. From the 100 ns molecular dynamics simulations trajectories we calculated densities of p-benzoquinone heavy atoms. By visually inspecting isodensity contours of density maps, we chose p-benzoquinone binding hotspots that had volume similar to p-benzoquinone and they were reasonably sequestered from the solvent. We used residues surrounding these hotspots to define ligand binding site during docking calculations. The top-ranked p-benzoquinone density maps are shown in Figure S15B. Structures of UQ2, idebenone and UQ10 were prepared using Schrodinger's Maestro Edit/Build panel. These structures were subsequently energy minimized using LigPrep software (Schrodinger Inc). As stated above, we used the residues surrounding the p-benzoquinone binding hotspot to define the ligand binding site. We docked UQ2 using the software Glide (Schrodinger Inc) with extra-precision (XP) scoring function. The top scoring pose was merged with the protein structure and saved for subsequent PRIME docking calculation. Docking larger ligands such as UQ10 using standard docking programs like Glide is particularly challenging due to inadequate conformation sampling. Idebenone and UQ10 contain 13 and 31 rotatable bonds respectively. For instance, Glide failed to dock UQ10. Therefore, we used PRIME docking software to dock both idebenone and UQ10. In PRIME docking, we retain portions of the ligand that are already present in the protein binding site, build and optimize the remaining portions of the ligand. For example, we retain the p-benzoquinone moiety of the UQ2 in the protein binding site (obtained from the Glide docking results vide-supra), build and optimize idebenone and UQ10. The PRIME docking methodology has already been reported in the literature (9).

Luciferase reporter assay

For assays in HEK293T cells, 25 ng UAS-firefly luciferase plasmid, 25 ng CMV-Gal4(DBD)-PPAR(LBD) plasmid, 0.5 ng SV40-renilla luciferase plasmid, and 150 ng polyethylenimine were used to transfect a well of HEK293T cells plated in 96-well plates.

One day after transfection, cells were treated with different PPAR agonists for another twenty-four hours before they were lysed in 100 μL passive lysis buffer, 50 μL of which was used to measure firefly luciferase activity and another 50 μL for renilla luciferase activity.

Minimally invasive transverse aortic constriction (TAC) surgery

Minimally invasive TAC surgery was performed to induce pressure overload–mediated cardiac hypertrophy as previously described (10). Briefly, mice were anesthetized with a single intraperitoneal injection of ketamine (51.4 mg/kg) and xylazine (3.3 mg/kg). After confirming adequate anesthesia by the absence of the toe-pinch reflex, the neck and chest were shaved and disinfected with 70% ethanol. The animal was placed in a supine position, and a 10-mm longitudinal midline cervical incision was made from the suprasternal notch toward the mid-sternum to expose the sternum. A 3–4 mm upper partial sternotomy was performed using a bone nipper, and the sternal edges were retracted to expose the aortic arch under low magnification. The thymus and surrounding soft tissues were gently displaced to visualize the transverse aorta between the right innominate and left common carotid arteries. A 6-0 silk suture was passed under the aortic arch using a ligation aid and tied around the vessel over a blunted 27-gauge needle to standardize the constriction. The needle was then removed, resulting in approximately 0.4-mm luminal narrowing of the aorta. The sternum was closed with 6-0 polypropylene sutures, and the skin was closed with a continuous 5-0 polypropylene suture. Sham-operated mice underwent the same procedure without tightening the ligature around the aorta. Animals were allowed to recover under warming conditions and received postoperative analgesia with buprenorphine (0.1 mg/kg).

Statistical analysis

The number of samples for each experimental condition is listed in the corresponding figure legend. Statistical significance was determined using one-way ANOVA followed by Šídák's multiple comparisons test (Fig. 2E and 3E). Differential expression analysis of RNA-seq data was performed using DESeq2. P values were calculated using the Wald test (Fig. 6B, S13, and S14). The remaining figures were analyzed using Student's t test. Error bars are represented as standard error of the mean.

Supplementary References

1. D. S. Sohal, *et al.*, Temporally regulated and tissue-specific gene manipulations in the adult and embryonic heart using a tamoxifen-inducible Cre protein. *Circ. Res.* **89**, 20–25 (2001).

2. M. Pashmforoush, *et al.*, Nkx2-5 pathways and congenital heart disease. *Cell* **117**, 373–386 (2004).

3. K. Hirose, *et al.*, Evidence for hormonal control of heart regenerative capacity during endothermy acquisition. *Science* **364**, 184–188 (2019).

4. K. Nishida, *et al.*, p38α mitogen-activated protein kinase plays a critical role in cardiomyocyte survival but not in cardiac hypertrophic growth in response to pressure overload. *Mol. Cell. Biol.* **24**, 10611–10620 (2004).

5. D. Miranda-Silva, V. Sequeira, A. P. Lourenço, I. Falcão-Pires, Assessing rodent cardiac function *in vivo* using hemodynamic pressure-volume loops. *Front. Physiol.* **12,** 751326 (2022).

6. G. Kleiner, *et al.*, CoQ10 supplementation rescues nephrotic syndrome through normalization of H2S oxidation pathway. *Biochim. Biophys. Acta Mol. Basis Dis.* **1864**, 3708–3722 (2018).

7. M. Mizutani, *et al.*, Identification of proteins involved in intracellular ubiquinone trafficking in Saccharomyces cerevisiae using artificial ubiquinone probe. *Biochim. Biophys. Acta Bioenerg.* **1865**, 149147 (2024).

8. P. Ghanakota, H. A. Carlson, Driving structure-based drug discovery through cosolvent molecular dynamics. *J. Med. Chem.* **59**, 10383–10399 (2016).

9. C. Rapp, C. Kalyanaraman, A. Schiffmiller, E. L. Schoenbrun, M. P. Jacobson, A molecular mechanics approach to modeling protein–ligand interactions: relative binding affinities in congeneric series. *J. Chem. Inf. Model.* **51**, 2082–2089 (2011).

10. R. Tavakoli, S. Nemska, P. Jamshidi, M. Gassmann, N. Frossard, Technique of minimally invasive transverse aortic constriction in mice for induction of left ventricular hypertrophy. *J. Vis. Exp.* **127**, 56231 (2017).

Supplementary Figures

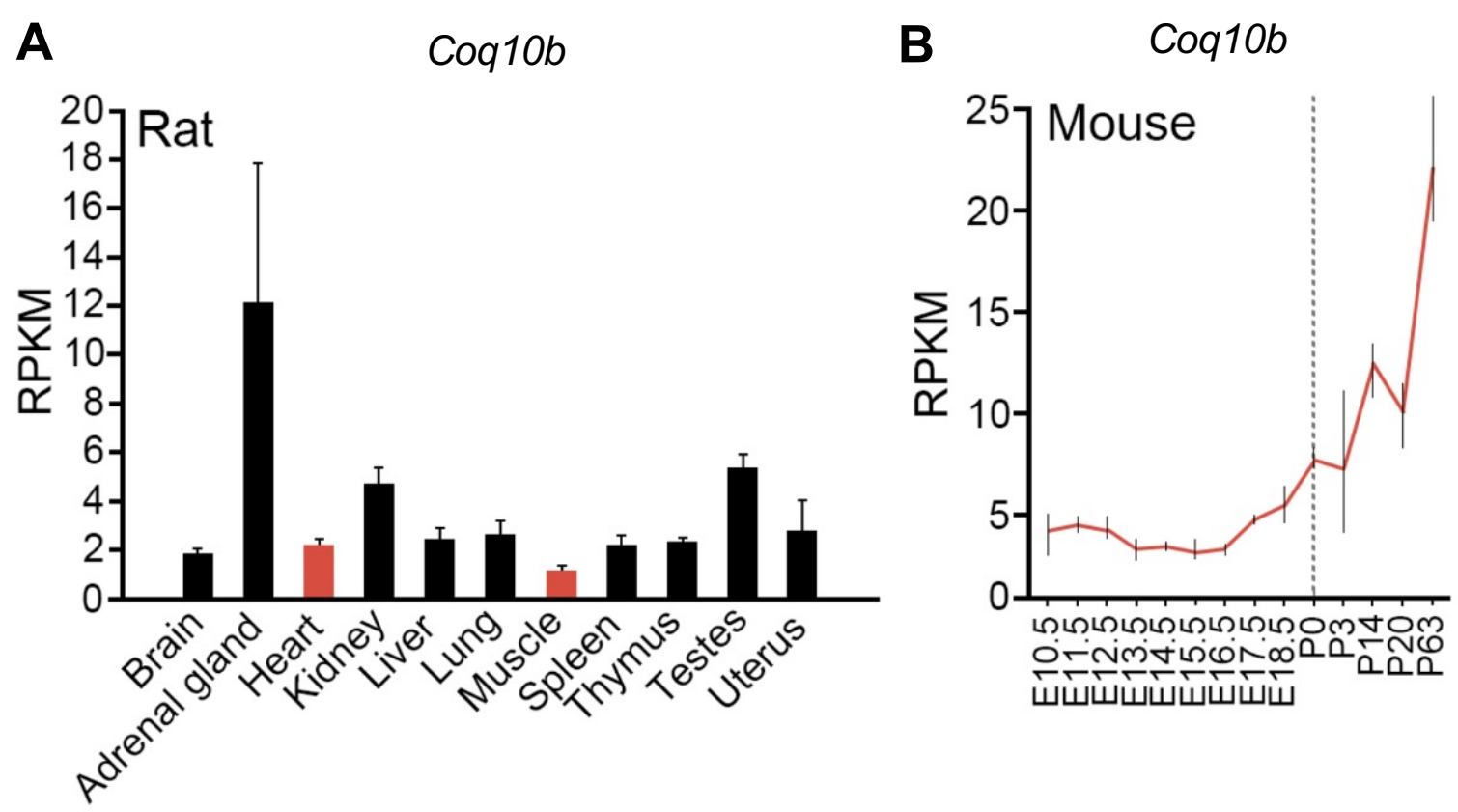

Figure S1. *Coq10b* expression.
(A) *Coq10b* expression in eleven adult rat organs (n=8).
(B) Temporal changes in *Coq10b* expression from embryonic day (E) 10.5 to postnatal day (P) 63 in the mouse heart (n=4).
Values are reported as mean ± SEM.

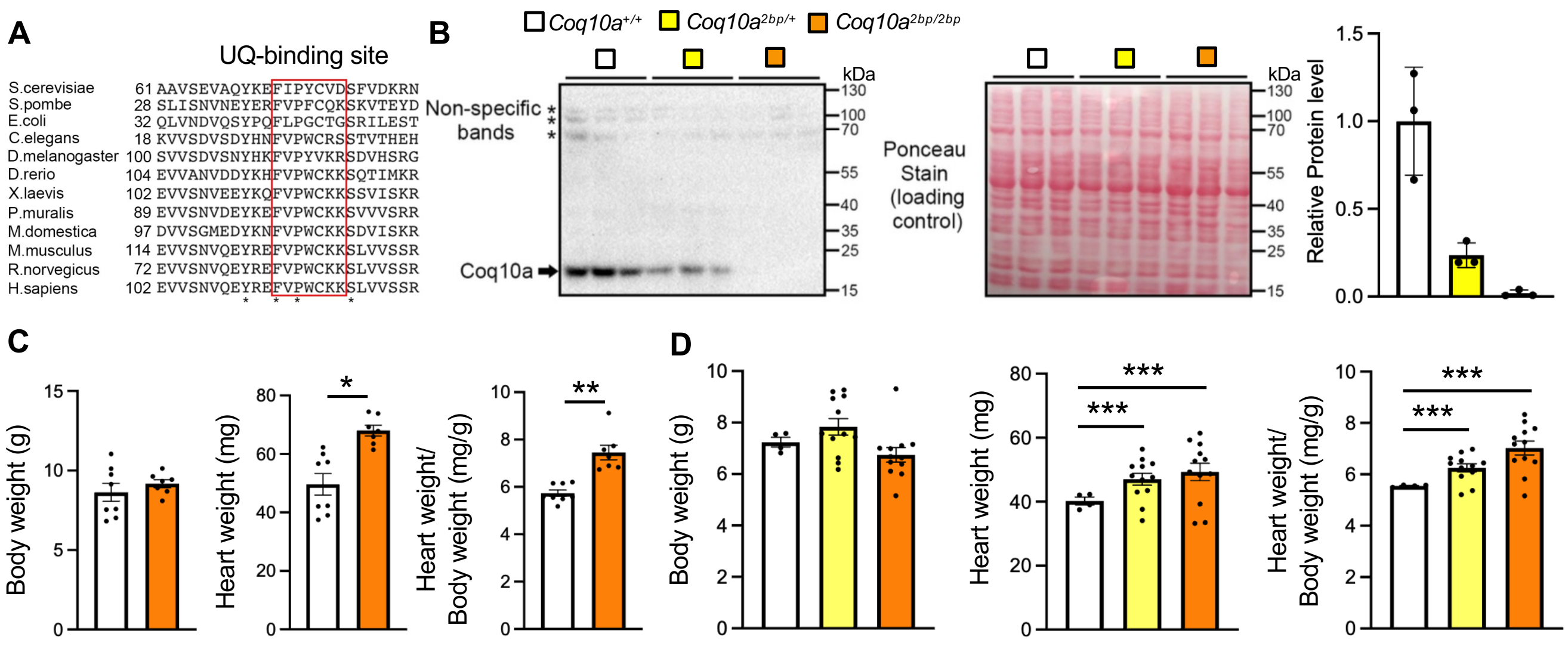

Figure S2. Additional validation and phenotypic analyses of *Coq10a$^{2bp/2bp}$* knockout mice.
(A) Alignment of bacteria, yeast, and vertebrate Coq10a protein sequences near the ubiquinone (UQ)-binding site. The red box marks the conserved UQ-binding site.
(B) Uncropped western blot images corresponding to Figure 4B (left). Coq10a protein levels were normalized to total protein loading assessed by Ponceau S staining (Right). (n=3 hearts per genotype).
(C) Measurements of the body weight (BW), heart weight (HW), and HW/BW ratio in the F0 generation of *Coq10a$^{+/+}$* wild-type and *Coq10a$^{2bp/2bp}$* homozygous mice at P14 (*Coq10a$^{+/+}$*; n=8 animals, *Coq10a$^{2bp/2bp}$*; n=7 animals)
(D) Measurements of the BW, HW, and HW/BW ratio in *Coq10a$^{+/+}$* wild-type, *Coq10a$^{+/2bp}$* heterozygous and *Coq10a$^{2bp/2bp}$* homozygous mice at P14 (*Coq10a$^{+/+}$*; n=4 hearts, *Coq10a$^{+/2bp}$*; n=12 hearts, *Coq10a$^{2bp/2bp}$*; n=12 hearts)
Values are reported as mean ± SEM. *P<0.05, **P<0.01, ***P<0.001

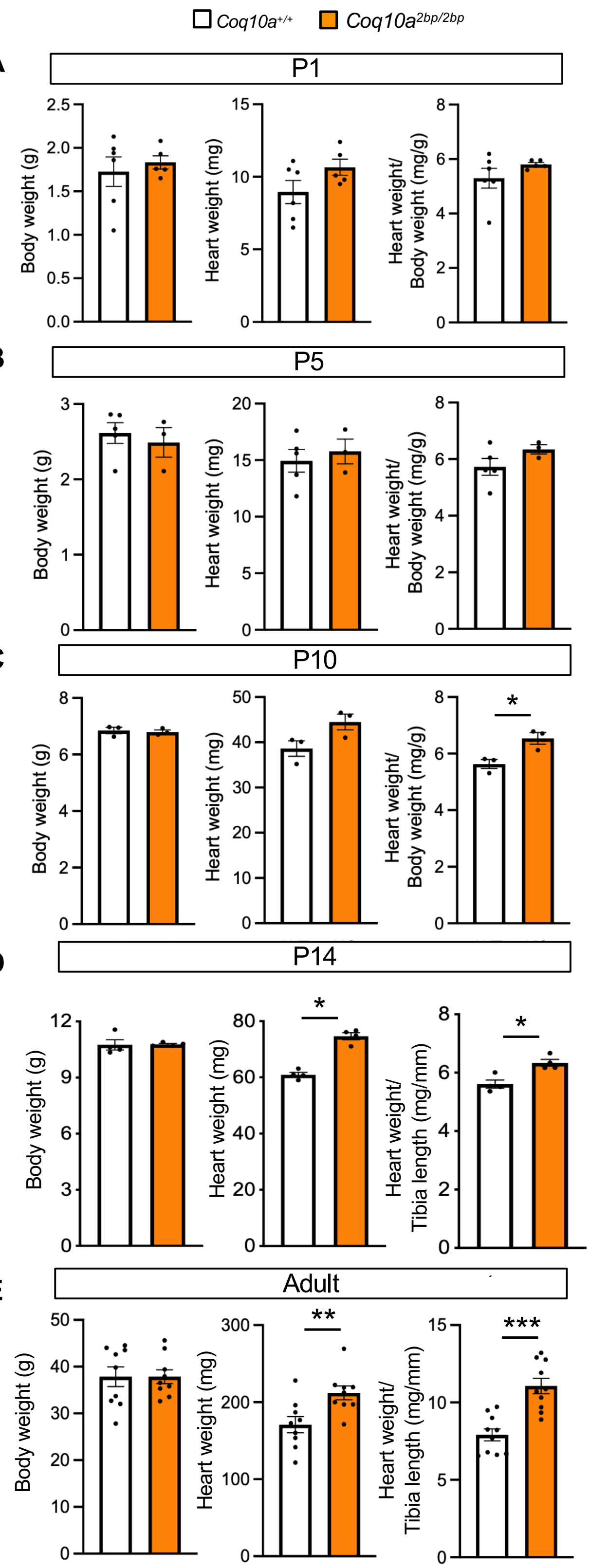

Figure S3. Time course of the cardiac hypertrophic phenotype in *Coq10a$^{2bp/2bp}$* knockout mice. (A-C) Measurements of body weight (BW), heart weight (HW), and heart weight-to-body weight ratio (HW/BW) of P1 (*Coq10a$^{+/+}$*, n = 3 animals; *Coq10a$^{2bp/2bp}$*, n = 4 animals), P5 (*Coq10a$^{+/+}$*, n = 5 animals; *Coq10a$^{2bp/2bp}$*, n = 3 animals), and P10 (*Coq10a$^{+/+}$*, n = 3 animals; *Coq10a$^{2bp/2bp}$*, n = 3 animals) mice. (D and E) Measurements of the BW, HW, and HW/Tibia length ratio in *Coq10a$^{+/+}$* and *Coq10a$^{2bp/2bp}$* mice at P14 (*Coq10a$^{+/+}$*; n=4 animals; *Coq10a$^{2bp/2bp}$*, n=4 animals) and in adulthood (*Coq10a$^{+/+}$,* n=9 animals; *Coq10a$^{2bp/2bp}$,* n=9 animals)
Values are reported as mean ± SEM. **P*<0.05, ***P*<0.01, ****P*<0.001

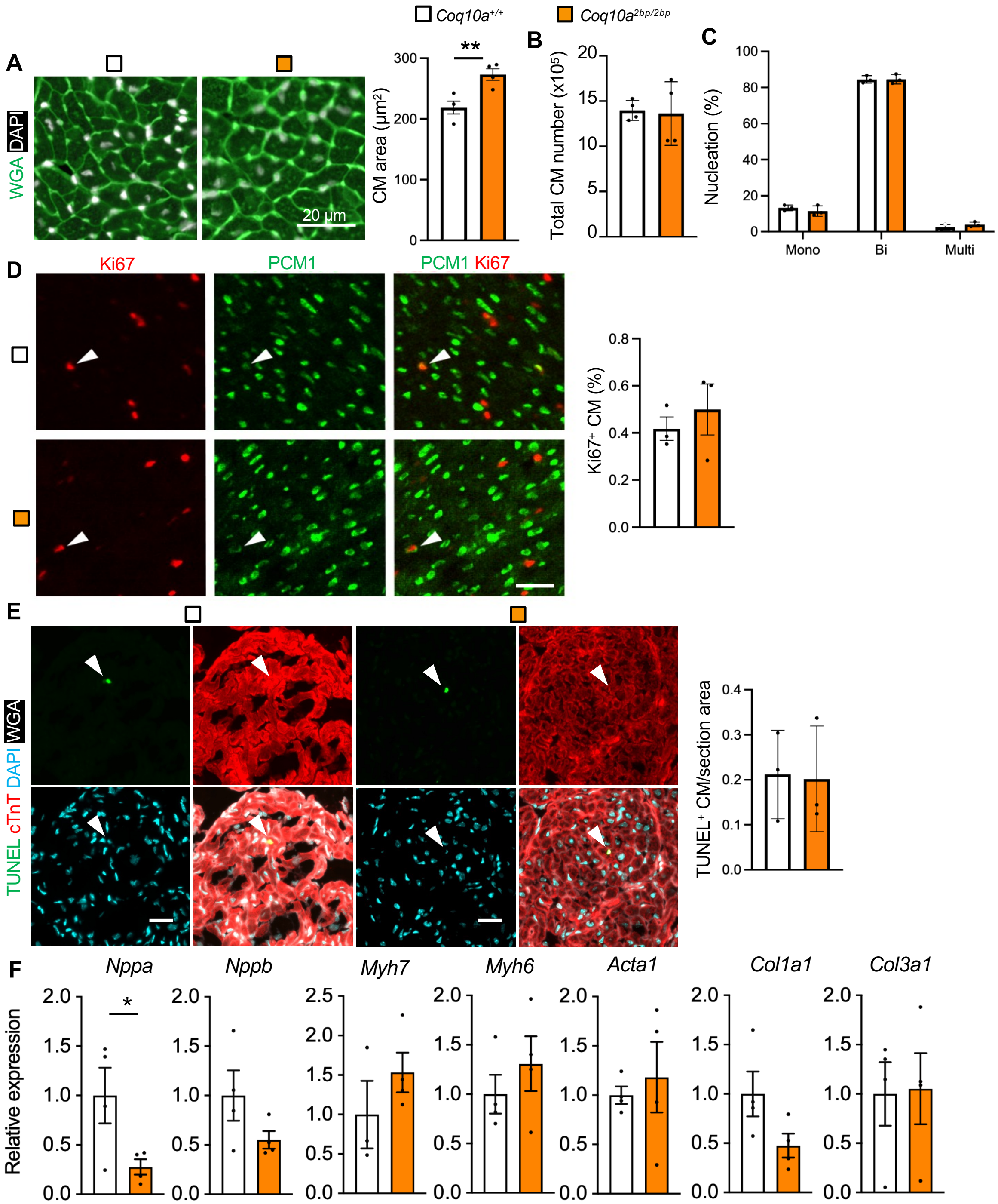

Figure S4. Cardiomyocyte number, nucleation, proliferation, and apoptosis in *Coq10a*$^{2bp/2bp}$ knockout mice at postnatal day 14.

(A) Representative WGA- and DAPI-stained P14 heart cross sections for CM area analysis. Quantified results shown on the right.

(B) Cardiomyocyte number analysis in P14 hearts (n=4 hearts each group).

(C) CM nucleation analysis of P14 hearts (n=3 hearts each group). Mono, Bi and Multi represent mononucleated, binucleated, and multinucleated CMs, respectively.

(D) Analysis of CM proliferative activity. Representative images of proliferating CMs (left). Arrowheads indicate Ki67+ CMs. Quantification of proliferating CMs that are double positive for Ki67 and PCM1 (n=3 hearts each group) (right).

(E) TUNEL staining and quantification results (n=4 hearts each group).

(F) RT-qPCR analysis of genes associated with pathological cardiac remodeling in heart tissue (n=4 hearts each group, except for *Myh7* panel, where *Coq10a*$^{+/+}$ hearts, n = 3, and *Coq10a*$^{2bo/2bp}$ hearts, n = 4). Values are reported as mean ± SEM. **P*<0.05, ***P*<0.01. Scale bar, 20 µm (A), 50 µm (D), and 20 µm (E).

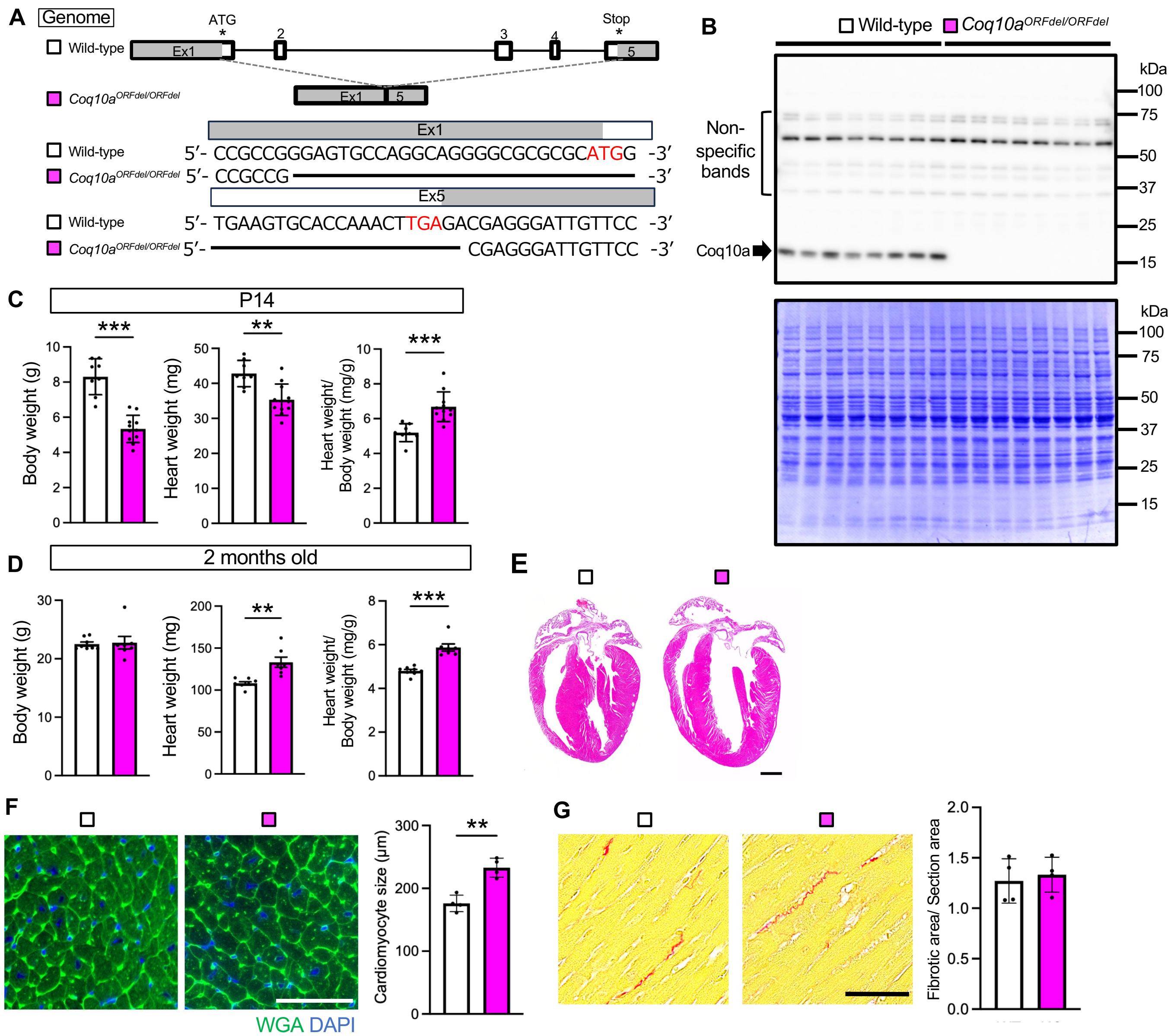

Figure S5. Cardiac phenotype of *Coq10a*$^{ORFdel/ORFdel}$ mice.
(A) Schematic for generating the open-reading frame deletion of the Coq10a gene. The CRISPR-Cas9 system targeted exon 1 and exon 5, leading to excision of the Coq10a open reading frame in a line generated in B6D2 fertilized eggs and subsequently backcrossed onto the C57BL/6N background.
(B) Western blot analysis of Coq10a protein in adult wildtype and *Coq10a*$^{ORF\ del/ORF\ del}$ hearts (Top) and the Coomassie blue staining for detection of loading protein (n=8 hearts per group). Coq10a protein is undetectable in *Coq10a*$^{ORFdel/ORFdel}$ hearts.
(C) Measurements of the body weight, heart weight, and ratio of heart weight to body weight at P14 (Wild-type; n=8 hearts, *Coq10a*$^{ORFdel/ORFdel}$; n=11 animals).
(D) Measurements of the body weight, heart weight, and ratio of heart weight to body weight at 2 months old (Wild-type; n=8 hearts, *Coq10a*$^{ORFdel/ORFdel}$; n=7 animals).
(E) Representative cross-section images (hematoxylin and eosin stained) of control wildtype and *Coq10a*$^{ORFdel/ORFdel}$ knockout hearts in adulthood.
(F) The Wheat Germ Agglutinin (WGA)-stained images for cardiomyocyte (CM) on cross-sections from adult hearts (left) and analysis of CM cross-section area (right) (n=4 hearts per group).
(G) Fibrosis analysis. Sirius red staining of heart sections (left). Quantification of fibrotic area per section area (right) (n=4 hearts per group). Values are reported as mean ± SEM. ***P*<0.01, ****P*<0.001, Scale bars, 1 mm (E), 50 μm (F) and 50 μm (G)

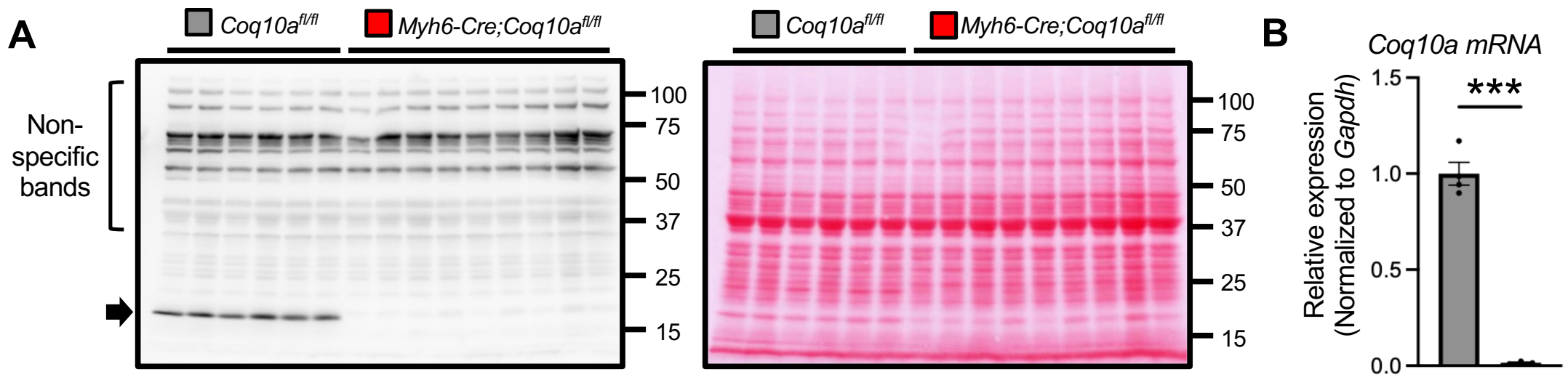

Figure S6. Uncropped western blot images and RT-qPCR data of *Myh6-Cre;Coq10a*$^{fl/fl}$ hearts.
(A) Uncropped western blot images corresponding to Figure 4I. (*Coq10a*$^{fl/fl}$, n=6 hearts, *Myh6-Cre;Coq10a*$^{fl/fl}$; n=9 hearts).
(B) Quantitative PCR analysis of Coq10a mRNA in *Coq10a*$^{fl/fl}$ and *Myh6-Cre;Coq10a*$^{fl/fl}$ ventricles at P14 (*Coq10a*$^{fl/fl}$, n=4 hearts, *Myh6-Cre;Coq10a*$^{fl/fl}$; n=3 hearts). One primer was designed within exon 3. Values are reported as mean ± SEM. ****P*<0.001.

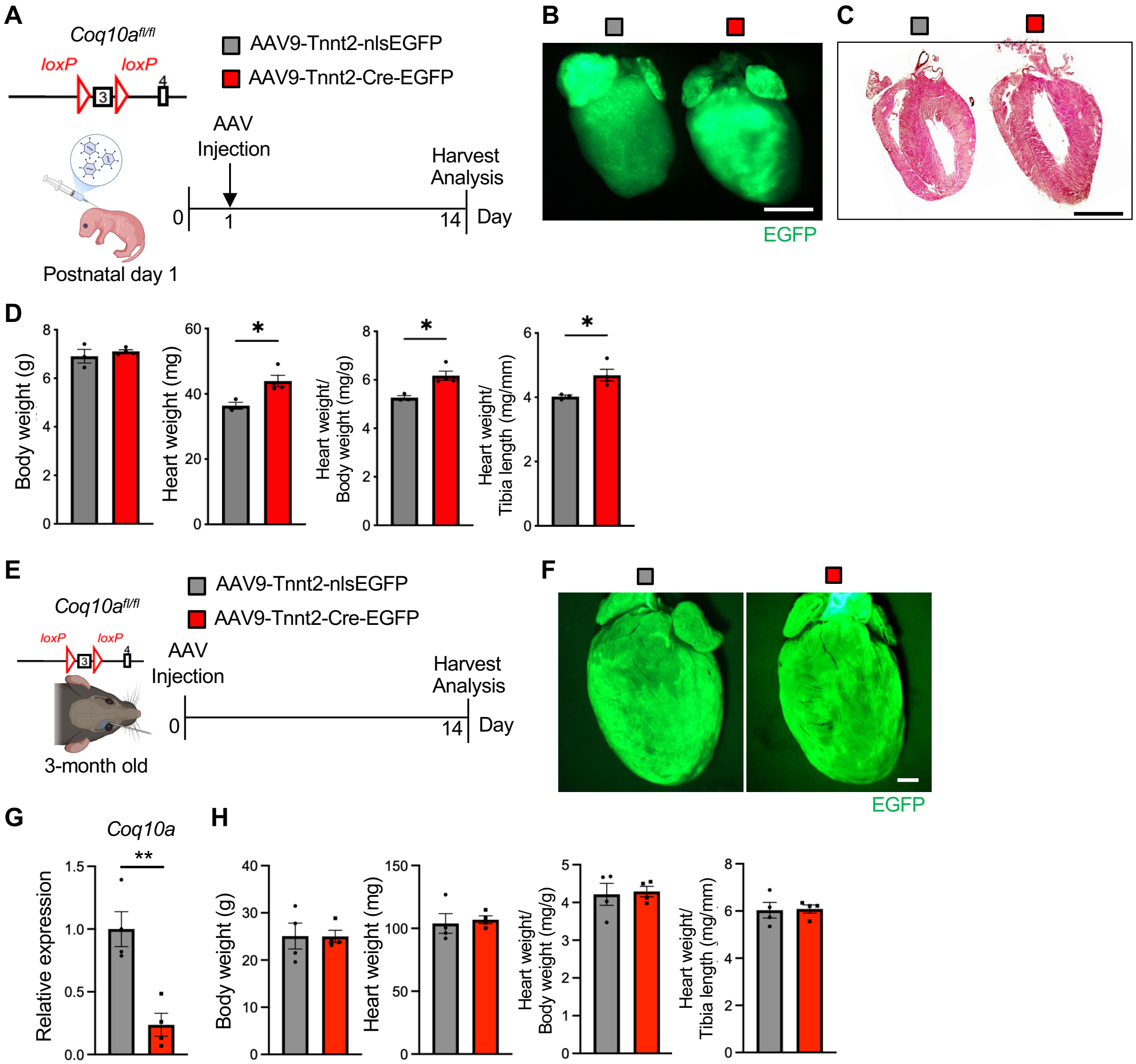

Figure S7. Cardiac phenotype of neonatal and adult *Coq10a*$^{fl/fl}$ mice two weeks after AAV9-Tnnt2-Cre-EGFP injection.

(A) Experimental design for conditional deletion of *Coq10a* in neonatal mice. *Coq10a*$^{fl/fl}$ pups were injected with AAV9-Tnnt2-Cre-EGFP or control AAV9-Tnnt2-nlsEGFP at postnatal day 1 (P1), and hearts were collected for analysis at P14.

(B) Representative whole-mount fluorescent images of P14 hearts.

(C) Representative hematoxylin and eosin (H&E) staining showing four-chamber views of the hearts.

(D) Quantification of body weight (BW), heart weight (HW), heart weight–to–body weight ratio (HW/BW), and heart weight–to–tibia length ratio (HW/TL) in neonatal mice (AAV9-Tnnt2-nlsEGFP, n= 3 animals; AAV9-Tnnt2-Cre-EGFP, n = 4 animals).

(E) Experimental design for conditional deletion of *Coq10a* in adult mice. Three-month-old *Coq10a*$^{fl/fl}$ mice were injected with AAV9-Tnnt2-Cre-EGFP or AAV9-Tnnt2-nlsEGFP and analyzed 14 days later.

(F) Representative whole-mount fluorescent images of adult hearts.

(G) RT–qPCR analysis of *Coq10a* expression in adult hearts (AAV9-Tnnt2-nlsEGFP, n = 4 hearts; AAV9-Tnnt2-Cre-EGFP, n = 4 hearts).

(H) Quantification of BW, HW, HW/BW, and HW/TL in adult mice (AAV9-Tnnt2-nlsEGFP, n = 4 animals; AAV9-Tnnt2-Cre-EGFP, n = 4 animals).

Values are presented as mean ± SEM. ns, not significant; **P* < 0.05, ***P* < 0.01. Scale bars, 2 mm (B, C, and F).

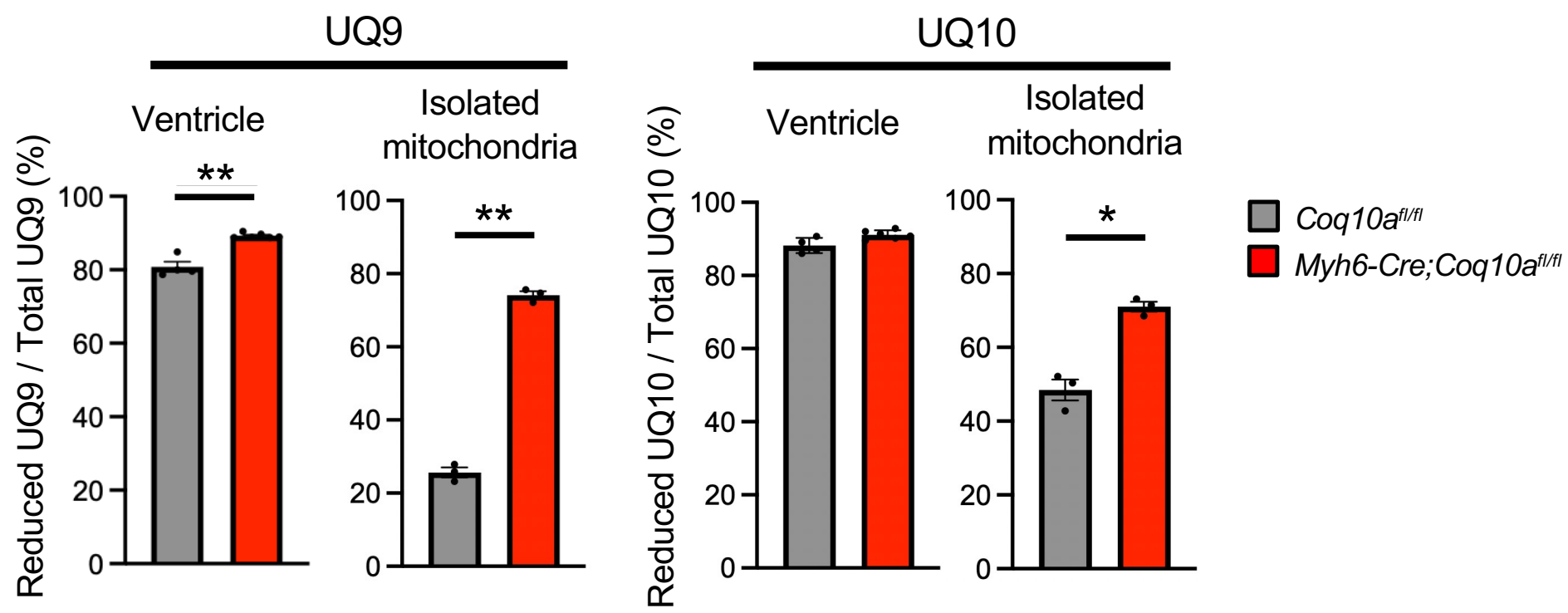

Figure S8. Percentage of reduced UQ9 and UQ10 in heart-specific Coq10a knockout mice. High-performance liquid chromatography (HPLC)-based detection of ubiquinone-9 (UQ9) and ubiquinone-10 (UQ10) in ventricle tissue (*Coq10a*$^{fl/fl}$, n=4 hearts; *Myh6-Cre;Coq10a*$^{fl/fl}$, n=6 hearts) and isolated mitochondria extracts from *Coq10a*$^{fl/fl}$ and *Myh6-Cre;Coq10a*$^{fl/fl}$ mice (n=3 hearts per group). Values are reported as mean ± SEM. **P*<0.05, ***P*<0.01.

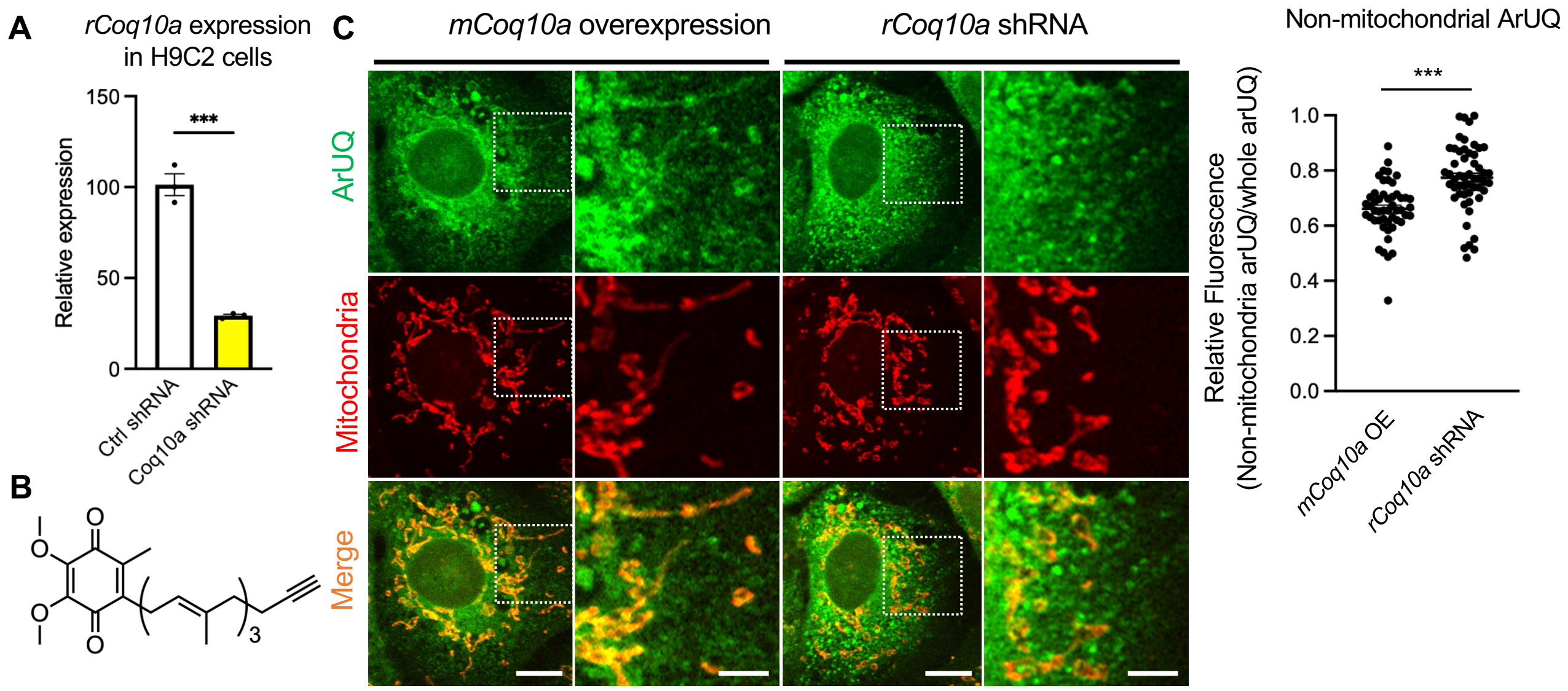

Figure S9. Detection of intracellular ubiquinone localization using artificial ubiquinone.
(A) Relative mRNA expression of rat *Coq10a* in control shRNA transfected and *Coq10a* shRNA-transfected H9C2 cells (n = 3).
(B) Chemical structure of artificial UQ probe.
(C) Visualization of subcellular UQ localization with artificial UQ probe in *Coq10a* shRNA-transfected and *Coq10a*-overexpressing H9C2 cells, and quantification of fluorescent signals of artificial UQ not overlapping with mitochondria (at least 100 cells counted).
Values are reported as mean ± SEM. ***$P<0.001$. Scale bars: 20 μm (C) and 4 μm (C, magnified images).

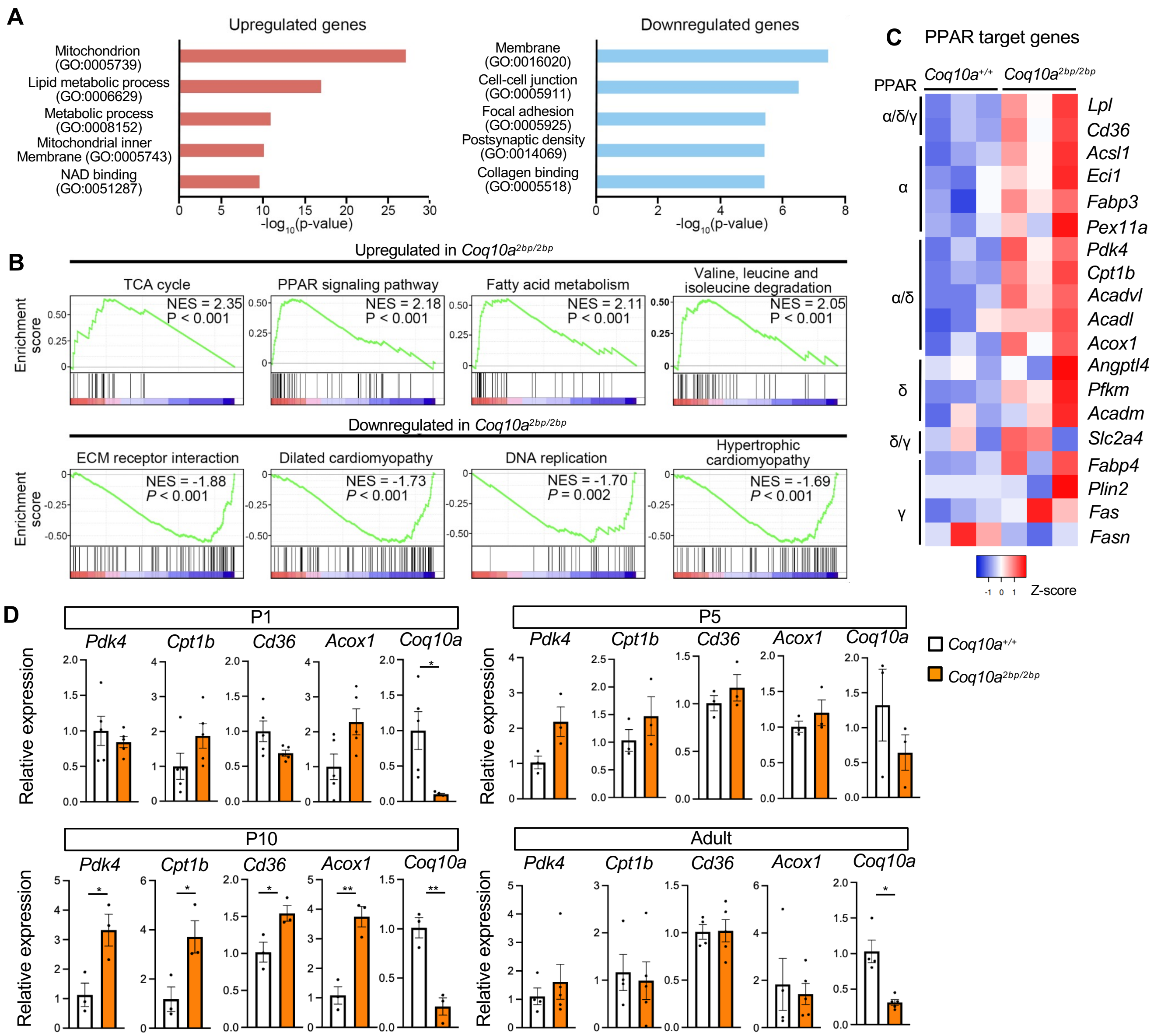

Figure S10. Transcriptomic analysis in *Coq10a*$^{2bp/2bp}$ hearts at P14.

(A) RNA-seq-based gene ontology analysis of upregulated and downregulated genes in the *Coq10a*$^{2bp/2bp}$ mutant heart.

(B) Gene set enrichment analysis of pathways altered in the *Coq10a*$^{2bp/2bp}$ heart.

(C) Heatmap of relative gene expressions of PPARα/γ/δ target genes in littermate control and *Coq10a*$^{2bp/2bp}$ hearts

(D) RT-qPCR analysis of PPARα target genes and *Coq10a* in wildtype control and *Coq10a*$^{2bp/2bp}$ hearts at P1, P5, P10, and adulthood (P1, n=4 hearts each group; P5, n = 3 hearts each group; P10, n = 3 hearts each group; Adult, n = 4 hearts each group).

Values are reported as mean ± SEM. **P*<0.05, ***P* <0.01.

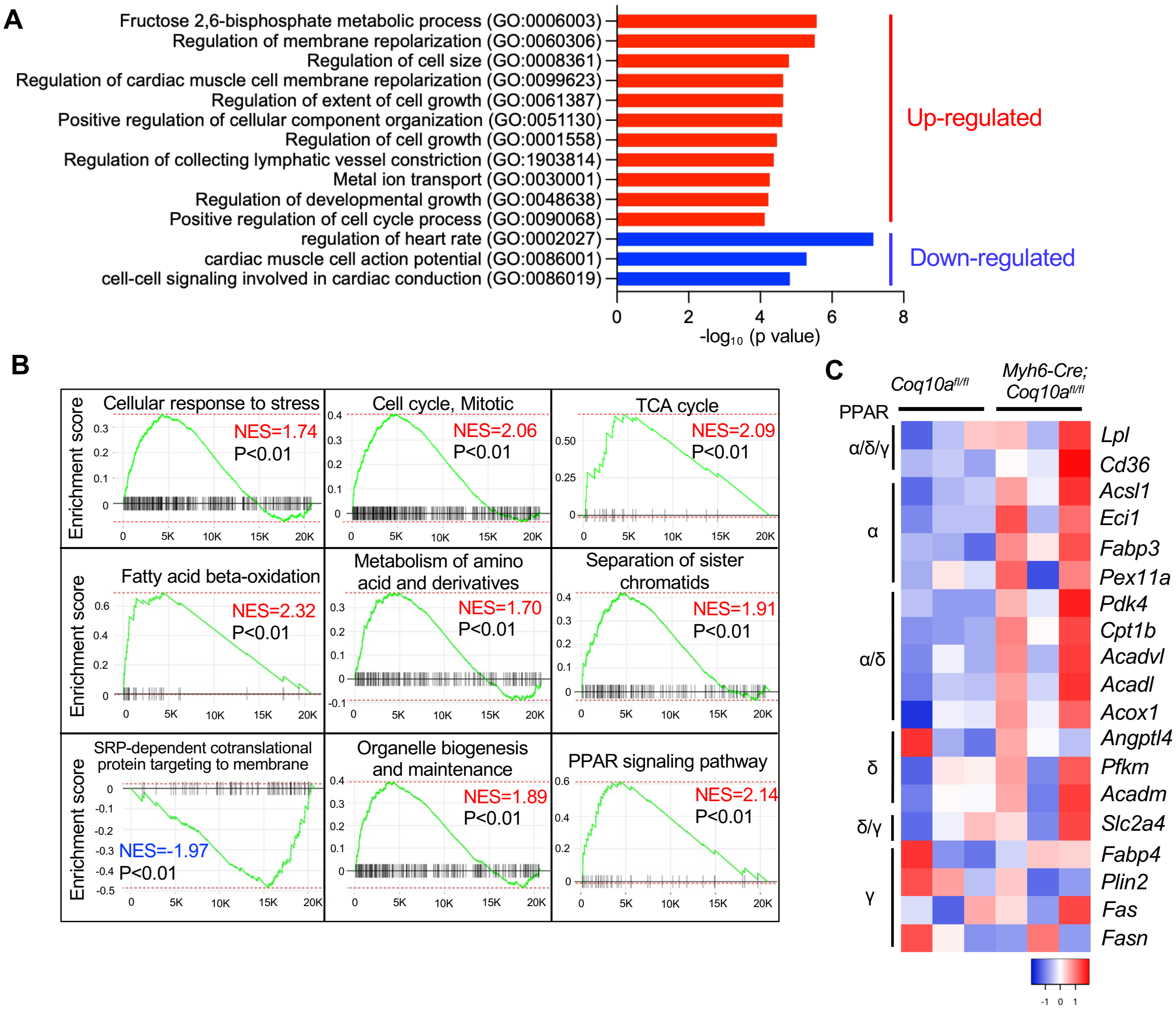

Figure S11. Transcriptomic analysis in *Myh6-Cre;Coq10a$^{fl/fl}$* hearts at P14.
(A) RNA-seq-based gene ontology analysis of upregulated and downregulated genes in *Myh6-Cre;Coq10a$^{fl/fl}$* hearts.
(B) Gene set enrichment analysis of pathways altered in *Myh6-Cre;Coq10a$^{fl/fl}$* hearts.
(C) Heatmap of relative gene expressions of PPARα/γ/δ target genes in littermate control and *Myh6-Cre;Coq10a$^{fl/fl}$* hearts. (n=3 hearts each group)

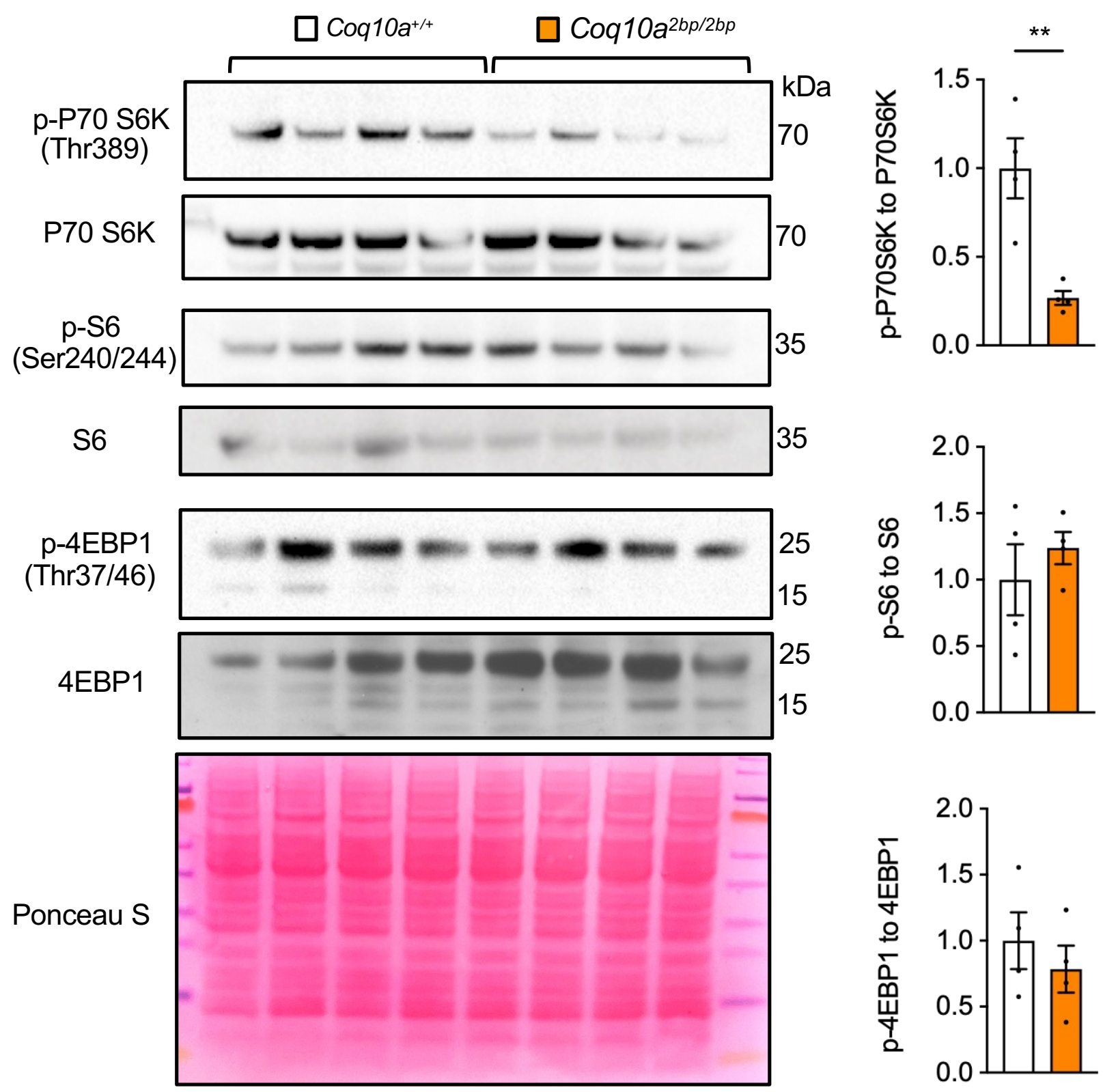

Figure S12. Canonical mTORC1 signaling is not activated in P14 *Coq10a$^{2bp/2bp}$* hearts. Representative immunoblots of phospho-p70S6K (Thr389), total p70S6K, phospho-S6 (Ser240/244), total S6, phospho-4E-BP1 (Thr37/46), and total 4E-BP1 in ventricular lysates from P14 *Coq10a$^{2bp/2bp}$* mice and wild-type controls. Ponceau S staining is shown as a loading control. Quantification of the phospho-p70S6K/total p70S6K, phospho-S6/total S6, and phospho-4E-BP1/total 4E-BP1 ratios (n=4 hearts each group). Data are presented as mean ± SEM. ***P*<0.01.

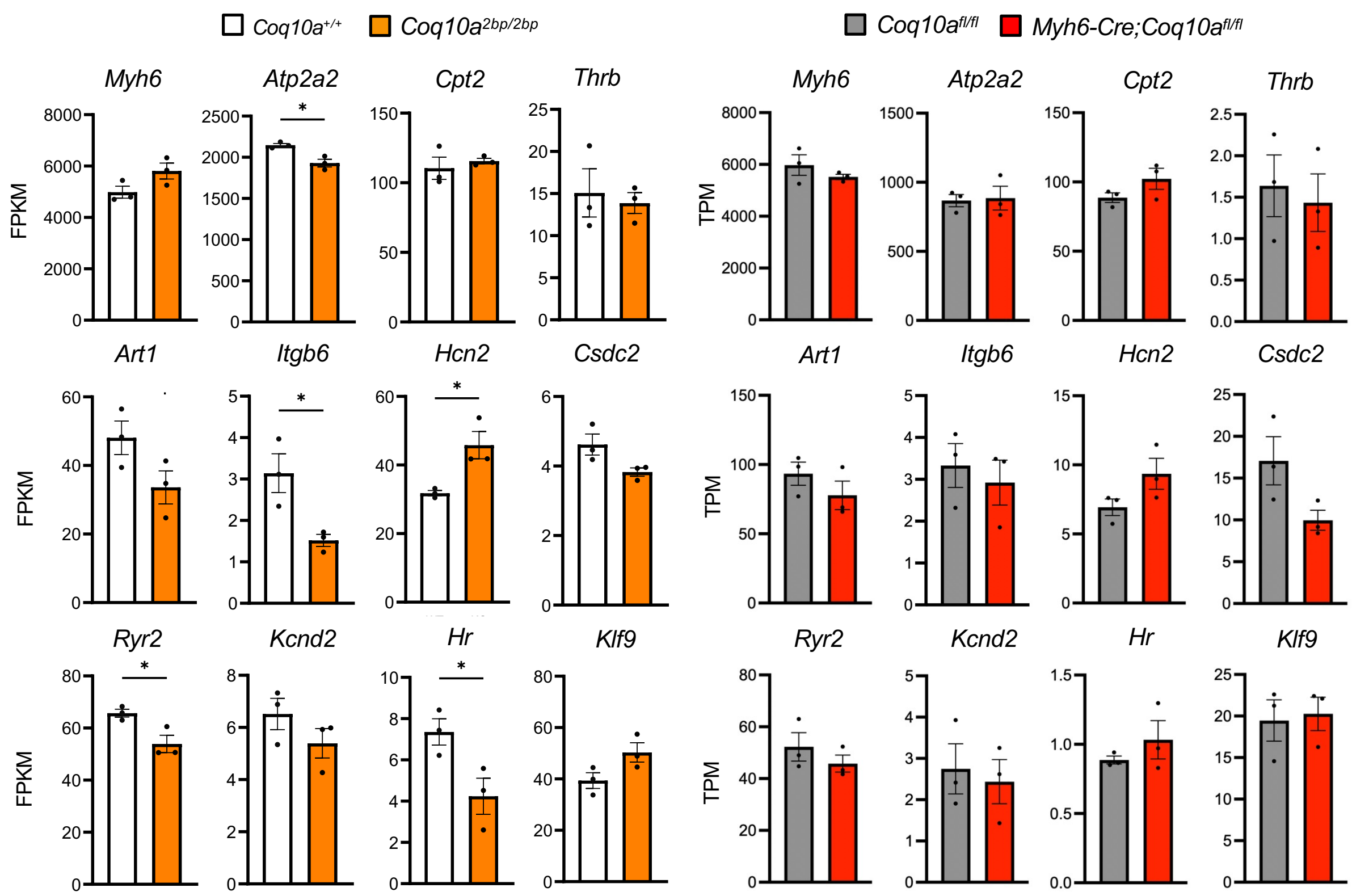

Figure S13. Expression of thyroid hormone-dependent genes in *Coq10a*-deleted hearts at P14. Expression of representative thyroid hormone-dependent genes in P14 *Coq10a*$^{2bp/2bp}$ hearts and cardiomyocyte-specific *Coq10a* knockout hearts (n=3 hearts each group). Expression values are shown as FPKM for the *Coq10a*$^{2bp/2bp}$ dataset and TPM for the *Myh6-Cre;Coq10a*$^{fl/fl}$ dataset. Values are reported as mean ± SEM (n=3 hearts each group). *P<0.05

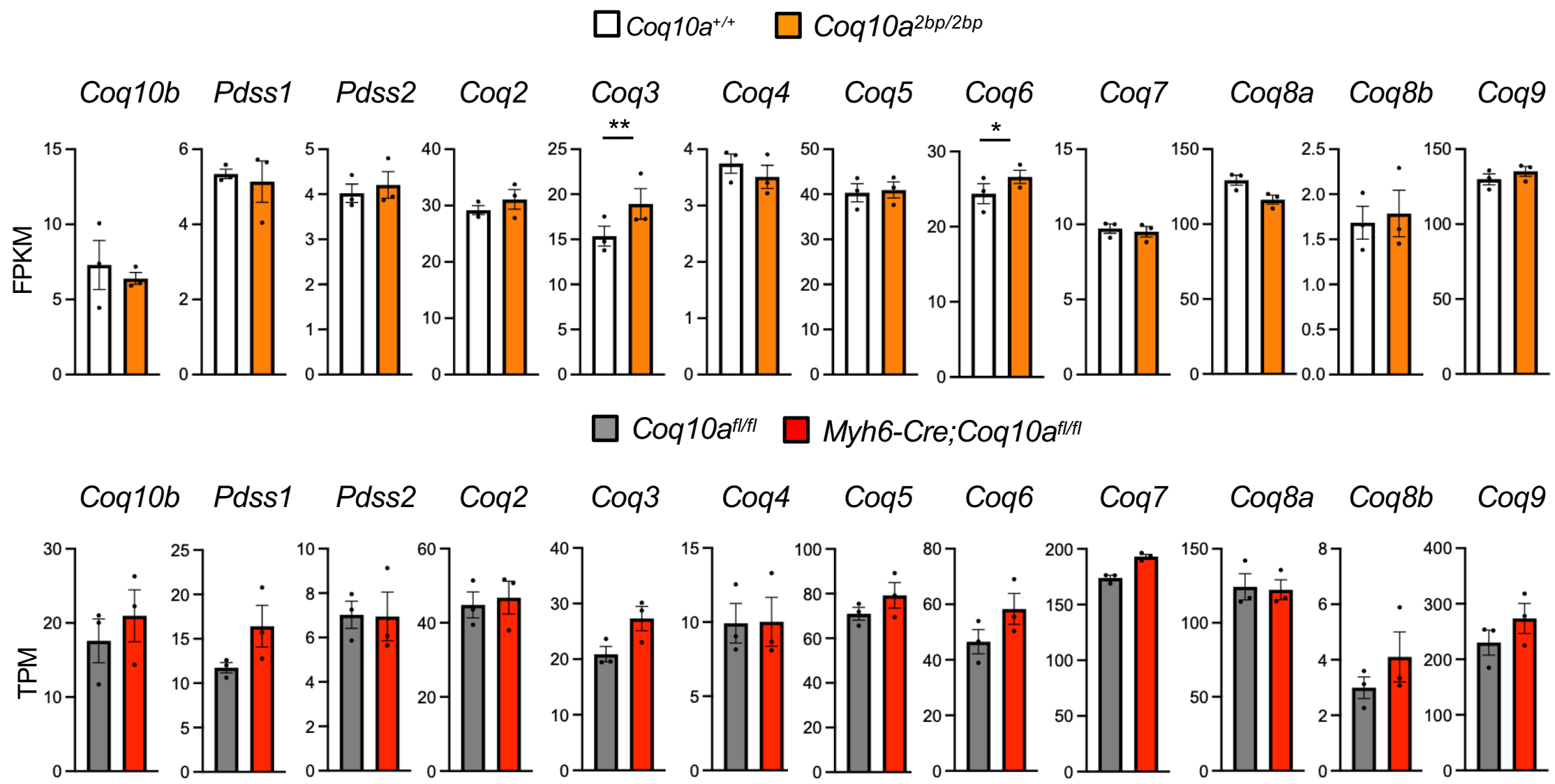

Figure S14. Expression of UQ biosynthesis-related genes in *Coq10a*-deleted hearts at P14. Expression of representative ubiquinone biosynthesis-related genes in P14 *Coq10a*$^{2bp/2bp}$ hearts and cardiomyocyte-specific *Coq10a* knockout hearts (n=3 hearts each group). Expression values are shown as FPKM for the *Coq10a*$^{2bp/2bp}$ dataset and TPM for the *Myh6-Cre;Coq10a*$^{fl/fl}$ dataset. Values are reported as mean ± SEM. *P<0.05, **P<0.01.

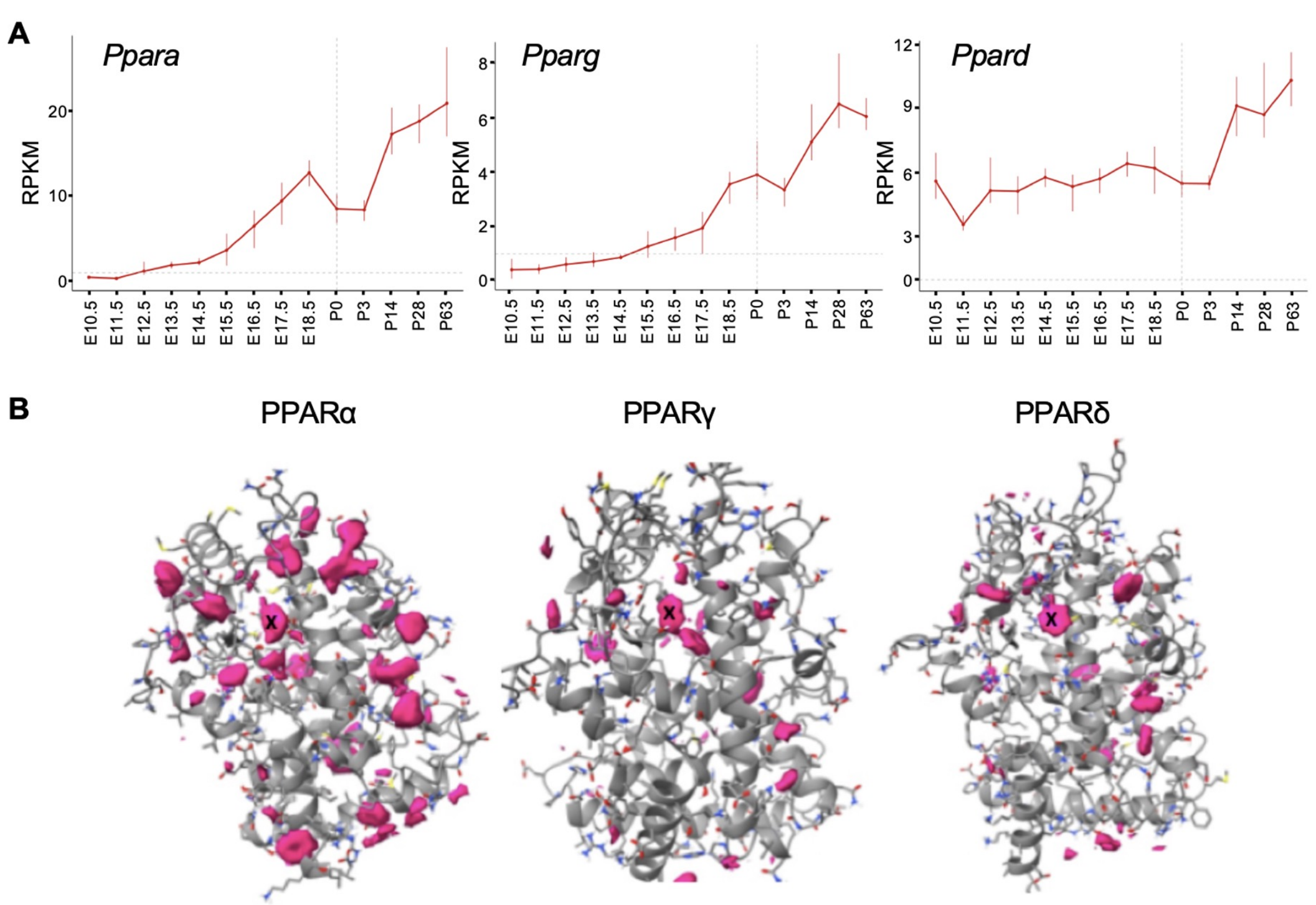

Figure S15. PPAR expression and ligand binding sites for UQ10 and idebenone.
(A) Developmental expression of *Ppara*, *Pparg*, and *Ppard* in the mouse heart.
(B) p-Benzoquinone density maps for PPARα, PPARγ, and PPARδ. A solvent-sequestered density hotspot was selected to define the quinone-headgroup binding site used for docking of UQ10 and idebenone (shown by X).

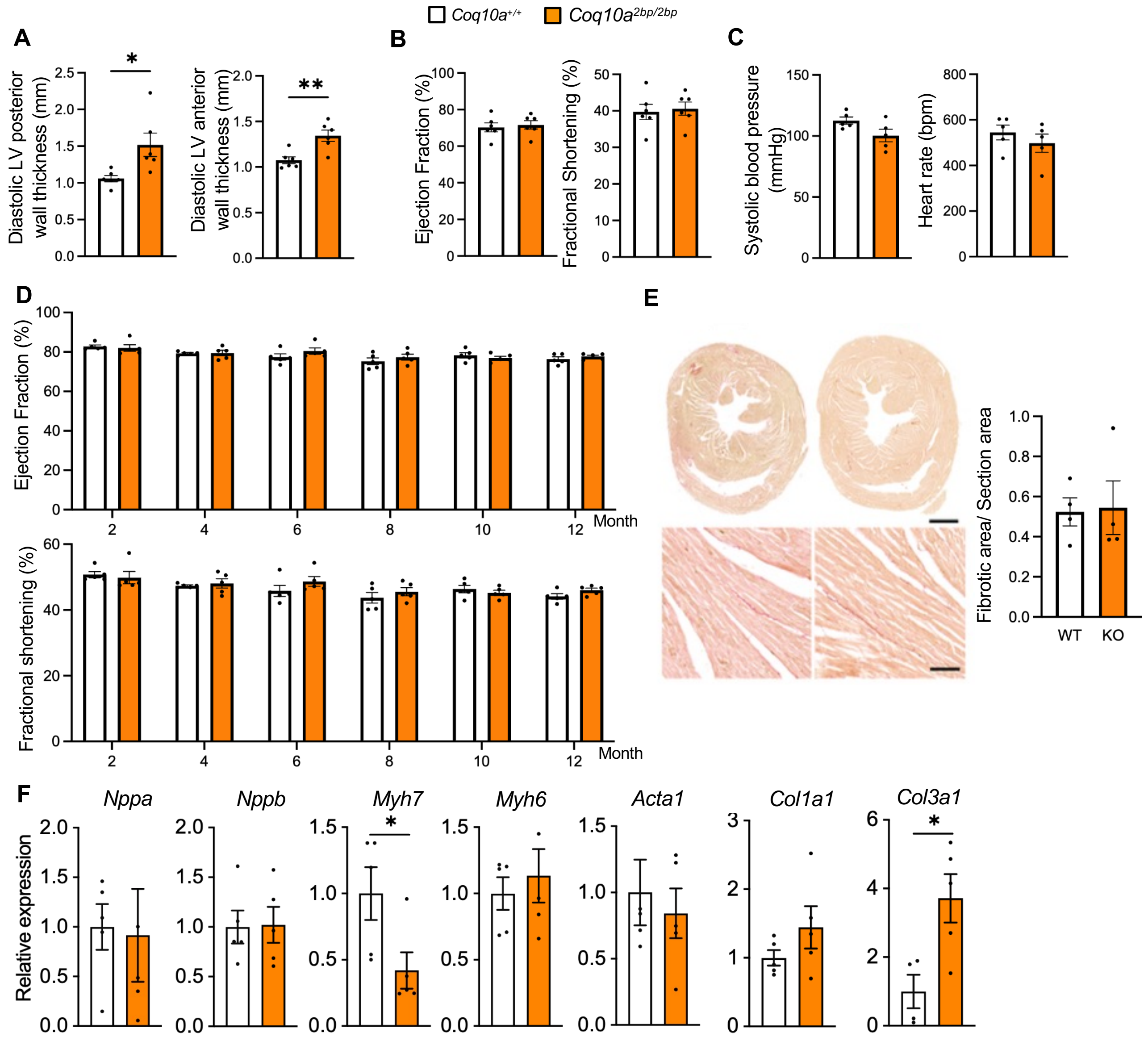

Figure S16. **Cardiac phenotype of *Coq10a* knockout mice in adulthood.**

(A) Diastolic left ventricle (LV) posterior/anterior wall thickness, analyzed by echocardiography in littermate control and *Coq10a*$^{2bp/2bp}$ animals (5-8 months old, n=6 animals per group).

(B) The ejection fraction and fractional shortening data analyzed by echocardiography in the littermate control and *Coq10a*$^{2bp/2bp}$ animals of individual animals (5-8 months old, n=6 animals per group).

(C) Measurements of systolic blood pressure and heart rate (5-8 months old, n=6 animals per group).

(D) Comparable cardiac contractile functions in *Coq10a* knockout and control mice over 12 months (n=5 animals per group).

(E) Fibrosis analysis. Sirius red staining of heart sections in diastole (left). Quantification of fibrotic area per section area (right) (Animals are 5-8 months old, n=4 animals per group)

(F) RT-qPCR analysis of genes associated with pathological cardiac remodeling in adult hearts (n=5 hearts each group, except for *Col3a1* panel, where *Coq10a*$^{+/+}$ hearts, n = 4, and *Coq10a*$^{2bo/2bp}$ hearts, n = 5). Values are reported as mean ± SEM. *P<0.05, **P<0.01.

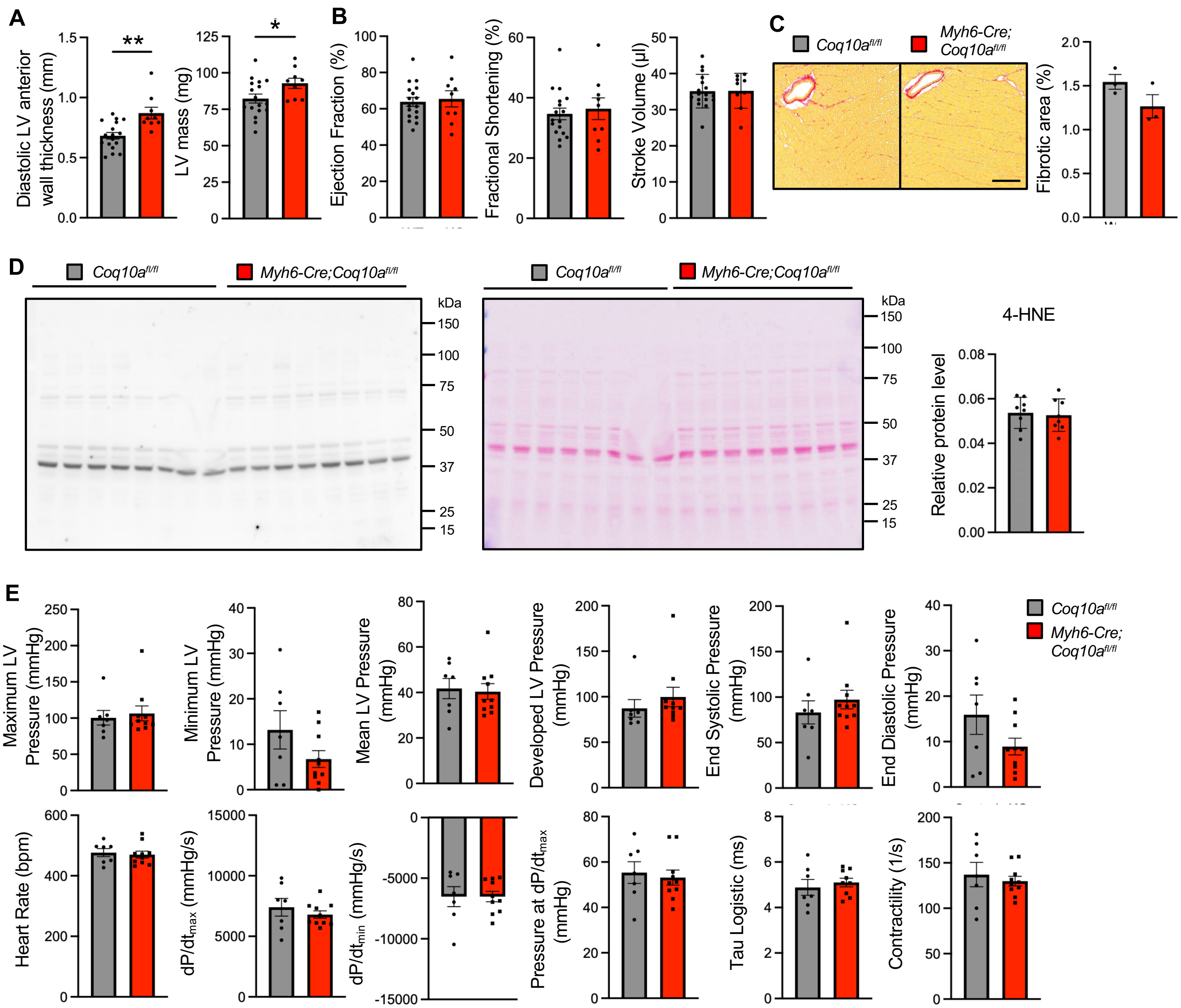

Figure S17. Measurement of cardiac function in *Myh6-Cre;Coq10a^fl/fl* mice.

(A) Diastolic left ventricle (LV) anterior wall thickness, analyzed by echocardiography in littermate *Coq10a^fl/fl* control and *Myh6-Cre;Coq10a^fl/fl* animals (1.5 - 3 months old, *Coq10a^fl/fl;* n=17 animals; *Myh6-Cre;Coq10a^fl/fl;* n=9 animals).

(B) The ejection fraction, fractional shortening data, stroke volume (µl) analyzed by echocardiography in the littermate control and *Myh6-Cre;Coq10a^fl/fl* animals (1.5 - 3 months old, *Coq10a^fl/fl;* n = 17 animals; *Myh6-Cre;Coq10a^fl/fl;* n=9 animals).

(C) Fibrosis analysis. Sirius red staining of heart sections (Left). Quantification of fibrotic area per section area (right). Animals are 1.5–3 months old (n=3 hearts per group).

(D) Western blotting for 4-hydroxynonenal (4-HNE) in ventricular lysates from *Coq10a^fl/fl* and *Myh6-Cre;Coq10a^fl/fl* mice (n=8 hearts per group). Blot image (left) and quantification of 4-HNE signal intensity normalized to Ponceau S intensity (right).

(E) Hemodynamic assessment by left ventricular pressure-volume loop analysis in littermate control and *Myh6-Cre;Coq10a^fl/fl* animals (1.5–3 months old, *Coq10a^fl/fl*; n = 7 animals; *Myh6-Cre;Coq10a^fl/fl*; n=10 animals). Representative pressure-volume loops (left) and quantification of maximum LV pressure, minimum LV pressure, mean LV pressure, developed LV pressure, end-systolic pressure, end-diastolic pressure, heart rate, dP/dt_{max}, dP/dt_{min}, pressure at dP/dt_{max}, Tau Logistic, and contractility index (right). Values are reported as mean ± SEM. *P<0.05, ** P <0.01.

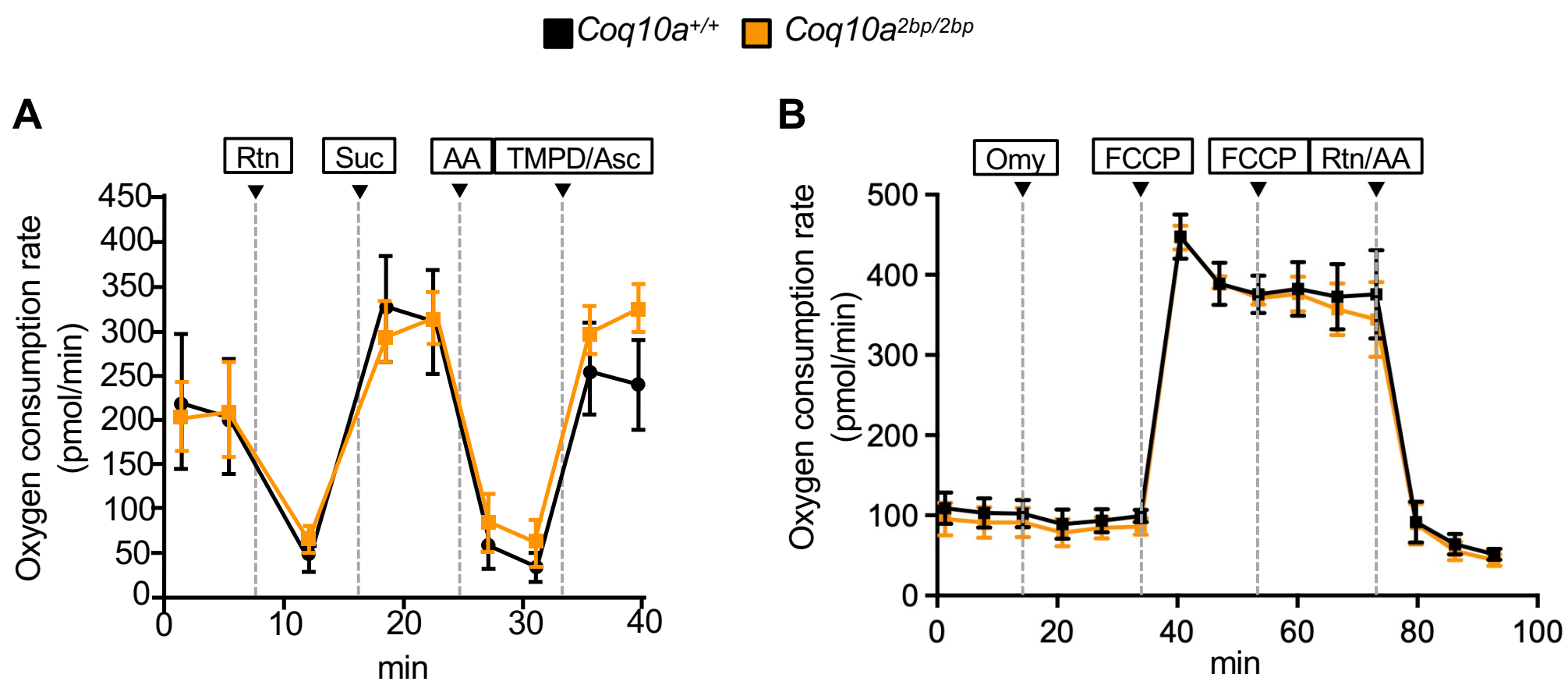

Figure S18. **Mitochondrial respiration in *Coq10a* knockout hearts.**
(A) Quantification of oxygen consumption rate (OCR) in isolated mitochondria (n=4 hearts per group). Arrows indicate the time of drug injections. Rtn, rotenone. Suc, succinate. AA, antimycin A. TMPD/Asc, TMPD/ascorbate.
(B) OCR in isolated adult cardiomyocytes. Cells were incubated for 1 hour prior to measurement in sodium bicarbonate-free DMEM supplemented with 4 mM glutamine, 100 U/ml penicillin, 100 μg/ml streptomycin, 1 mM sodium pyruvate, and 1% insulin–transferrin–selenium (ITS). OCR was measured using an XFe96/XF Pro FluxPak under basal conditions and following sequential injections of oligomycin (Omy, 1 μM), FCCP (0.5 μM), and antimycin A plus rotenone (AA/Rtn, 1 μM each). n = 4 hearts per group. Values are reported as mean ± SEM.

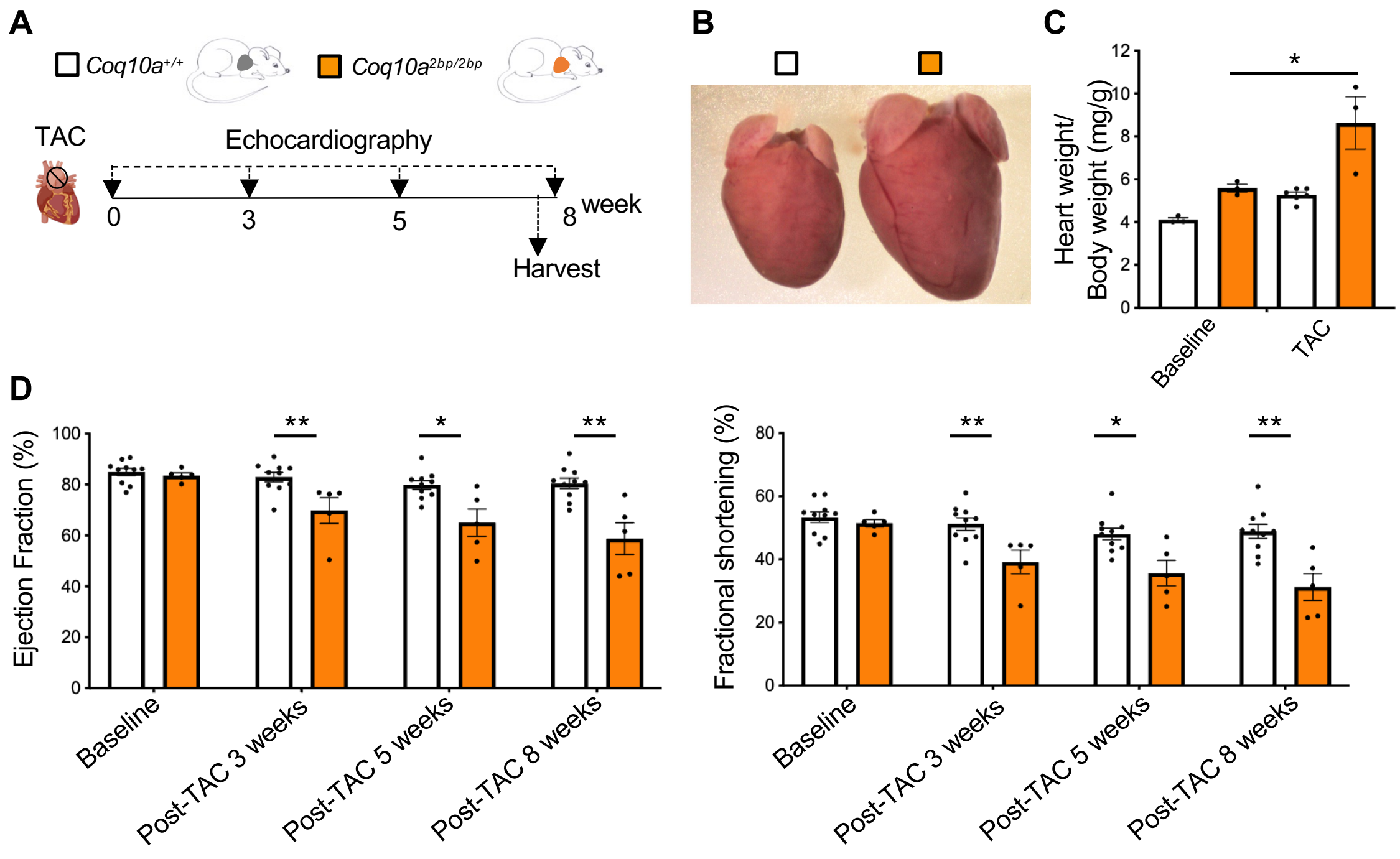

Figure S19. Cardiac phenotype of *Coq10a* knockout mice following transverse aortic constriction (TAC) surgery.

(A) Experimental scheme showing the TAC surgery and related measurements.

(B) Whole-mount images of wild-type (left) and knockout (right) hearts 8 weeks post-TAC surgery

(C) Measurement of heart weight-to-body weight ratio. (*Coq10a*$^{+/+}$, n = 3; *Coq10a*$^{2bp/2bp}$, n = 3; *Coq10a*$^{+/+}$ TAC, n = 6; *Coq10a*$^{2bp/2bp}$ TAC, n = 3)

(D) Measurement of heart function by echocardiography. (*Coq10a*$^{+/+}$, n = 10; *Coq10a*$^{2bp/2bp}$, n = 5). EF, Ejection fraction; FS, Fractional shortening; TAC: Transverse aortic constriction. Values are reported as mean ± SEM. *P<0.05, **P<0.01.

Legends for Dataset S1 to S4

Dataset S1 (separate file): Mouse and human tissue expression of mitochondrial protein-encoding genes.

Dataset S2 (separate file): Tissue-enriched postnatally upregulated mitochondrial protein-encoding genes.

Dataset S3 (separate file) : Differential gene expression analysis of P14 *Coq10a*$^{2bp/2bp}$ hearts.

Dataset S4 (separate file) : Differential gene expression analysis of P14 *Myh6-Cre;Coq10a*$^{fl/fl}$ hearts.
